# The translation initiation complex eIF3 in trypanosomatids and other pathogenic excavates – identification of conserved and divergent features based on orthologue analysis

**DOI:** 10.1186/1471-2164-15-1175

**Published:** 2014-12-23

**Authors:** Antonio M Rezende, Ludmila A Assis, Eduardo C Nunes, Tamara D da Costa Lima, Fabricio K Marchini, Eden R Freire, Christian RS Reis, Osvaldo P de Melo Neto

**Affiliations:** Centro de Pesquisas Aggeu Magalhães, Fundação Oswaldo Cruz, Avenida Professor Moraes Rego s/n, Cidade Universitária, Recife, PE 50670-420 Brazil; Functional Genomics Laboratory, Carlos Chagas Institute, Fiocruz, R. Prof. Algacyr Munhoz Mader, 3775, Curitiba, PR 81350-010 Brazil

**Keywords:** Translation initiation factor, Protein synthesis, eIF3, Protozoa

## Abstract

**Background:**

The initiation of translation in eukaryotes is supported by the action of several eukaryotic Initiation Factors (eIFs). The largest of these is eIF3, comprising of up to thirteen polypeptides (eIF3a through eIF3m), involved in multiple stages of the initiation process. eIF3 has been better characterized from model organisms, but is poorly known from more diverged groups, including unicellular lineages represented by known human pathogens. These include the trypanosomatids (*Trypanosoma* and *Leishmania*) and other protists belonging to the taxonomic supergroup Excavata (*Trichomonas* and *Giardia* sp.).

**Results:**

An in depth bioinformatic search was carried out to recover the full content of eIF3 subunits from the available genomes of *L. major*, *T. brucei*, *T. vaginalis* and *G. duodenalis*. The protein sequences recovered were then submitted to homology analysis and alignments comparing them with orthologues from representative eukaryotes. Eleven putative eIF3 subunits were found from both trypanosomatids whilst only five and four subunits were identified from *T. vaginalis* and *G. duodenalis*, respectively. Only three subunits were found in all eukaryotes investigated, eIF3b, eIF3c and eIF3i. The single subunit found to have a related Archaean homologue was eIF3i, the most conserved of the eIF3 subunits. The sequence alignments revealed several strongly conserved residues/region within various eIF3 subunits of possible functional relevance. Subsequent biochemical characterization of the *Leishmania* eIF3 complex validated the bioinformatic search and yielded a twelfth eIF3 subunit in trypanosomatids, eIF3f (the single unidentified subunit in trypanosomatids was then eIF3m). The biochemical data indicates a lack of association of the eIF3j subunit to the complex whilst highlighting the strong interaction between eIF3 and eIF1.

**Conclusions:**

The presence of most eIF3 subunits in trypanosomatids is consistent with an early evolution of a fully functional complex. Simplified versions in other excavates might indicate a primordial complex or secondary loss of selected subunits, as seen for some fungal lineages. The conservation in eIF3i sequence might indicate critical functions within eIF3 which have been overlooked. The identification of eIF3 subunits from distantly related eukaryotes provides then a basis for the study of conserved/divergent aspects of eIF3 function, leading to a better understanding of eukaryotic translation initiation.

**Electronic supplementary material:**

The online version of this article (doi:10.1186/1471-2164-15-1175) contains supplementary material, which is available to authorized users.

## Background

The initiation stage of protein synthesis in eukaryotes is a complicated process, which involves a great number of different macromolecules and requires the action of multiple eukaryotic Initiation Factors, eIFs. Many eIFs from model organisms such as *Drosophila*, budding yeast, mouse and *Arabidopsis*, have been well characterized and their role in translation initiation described. Some of these are single polypeptide factors whilst others are complexes of multiple subunits which can be very large and perform various roles in translation (more recently reviewed in [1–3]).

eIF3 is the largest of the initiation factors, both in size and in number of subunits, being active during multiple steps of the translation initiation process. In mammals, it is composed of 13 subunits (eIF3a through eIF3m) whilst in the budding yeast *S. cerevisae*, a reduced eIF3 is present composed of five essential subunits (eIF3a, eIF3b, eIF3c, eIF3g and eIF3i) and the non-essential eIF3j (reviewed in [2, 4–6]). The essential *S. cerevisae* subunits defined the core eIF3 complex present in all eukaryotes investigated so far. Nevertheless, *in vitro* reconstitution of mammalian eIF3 have implicated three conserved subunits (eIF3a, eIF3b, eIF3c) and three non-conserved subunits (eIF3e, eIF3f and eIF3h) as being required for eIF3 function, and constituting a functional mammalian eIF3 core, whilst indicating that the two conserved subunits eIF3g and eIF3i might be dispensable [7]. Indeed eIF3a, eIF3b and eIF3c have been proposed to occupy a central position in the overall mammalian eIF3 structure. In the absence of the loosely associated eIF3j, the mammalian complex seems to be organized in three stable modules: the first (module i), formed by the eIF3a, eIF3b, eIF3g and eIF3i subunits, resembling the budding yeast eIF3 core; a second module (ii), formed by subunits eIF3c, eIF3d, eIF3e, eIF3k and eIF3l; and a third smaller module (iii), formed by subunits eIF3f, eIF3h and eIF3m [8, 9]. Cryo-electron microscopic reconstruction of eIF3 has revealed that its subunits are organized in an anthropomorphic shape with five appendages and which shows surface complementarity to the platform of the 40S ribosomal subunit [10].

To accomplish its function, many of the eIF3 subunits participate in direct protein-protein interactions with other eIFs as well as ribosomal proteins, and also bind directly to RNA (reviewed in [2, 5]). Six of the mammalian eIF3 subunits (eIF3a, eIF3c, eIF3e, eIF3k, eIF3l and eIF3m) contain a PCI domain, a hallmark of related protein complexes such as the lid of the 19S regulatory particle of the 26S proteasome and the COP9 signalosome, which is involved in protein-protein interactions ( [11, 12]; reviewed in [13]). Recent evidence has also implicated the PCI domain in the binding of two distinct yeast eIF3 subunits (eIF3a and eIF3c) to RNA [14, 15] although in humans the same subunits were found to bind to RNA (HCV IRES) through independent RNA-binding HLH motifs [16]. Two other eIF3 subunits (eIF3f and eIF3h), containing the MPN domain, are members of a second family of proteins which also include subunits of the 26S proteasome and the signalosome, reflecting a common evolutionary origin for all three complexes ( [11, 12, 17]; also reviewed in [13]). It has been proposed that the six PCI and two MPN containing subunits constitute a structural core which binds to other eIF3 subunits, translation initiation factors and the 40S ribosomal subunit [18, 19]. Indeed a functional reconstitution of the human eIF3 has shown that these subunits can form a stable octameric complex [18], pointing out to a spontaneous assembly pathway for the eIF3 complex which, nevertheless, is compatible with the three modules concept described above.

Most of what is known in regard to eIF3 and its role in translation initiation within eukaryotes is derived from very few organisms, despite the fact that a remarkable diversity within the translation apparatus has been noticed across the different eukaryotic groups [20]. Not many studies, however, have been carried out focusing on excavates, unicellular protists which diverged very early from better studied unicellular and multicellular eukaryotes. These include many pathogenic species of medical and veterinary importance, such as those belonging to the family Trypanosomatidae (genus *Leishmania* and *Trypanosoma*) and even more divergent eukaryotes. The trypanosomatids have been seen to display unique biological characteristics rarely seen in eukaryotes. These include a scarcity of promoters for the protein coding genes, transcription of mRNAs in long polycystronic units, *trans* splicing of these polycystronic messages into monocystronic mature mRNAs and a lack of transcriptional control of their gene expression (recently reviewed in [21–24]). Regarding translation initiation, the study in trypanosomatids of eIF4E and eIF4G, two other initiation factors known to partner with eIF3 in the recruitment of mRNA by the ribosome, has highlighted novel and conserved events when compared to other eukaryotes (reviewed in [25, 26]). However, little is known regarding the eIF3 subunits of trypanosomatids and to what degree they are conserved in sequence or function when compared to better known eukaryotes.

The availability of complete or nearly complete genomic sequences for several selected trypanosomatids [27–29], as well as for other excavates of medical importance, such as *Giardia* sp. [30] and *Trychomonas* sp. [31], has generated new opportunities to investigate basic biological processes in pathogenic organisms which are distantly placed within the eukaryotic lineage. Studying translation initiation in these organisms may not only help define divergent features in specific stages, useful in the identification of potential therapeutical targets, but may also enhance the understanding regarding the evolution and conservation of the whole process throughout the eukaryotic lineages. Here, various bioinformatic tools were applied in order to perform a systematic search of the genomes of selected organisms for homologues of the various eIF3 subunits. The main targets were the two most complete trypanosomatid genomes available, those from *Leishmania major*[27] and *Trypanosoma brucei*[28], but studies were also carried out using the *Giardia duodenalis* (*synonymous* to *G. lamblia* and *G. intestinalis* – [32] and *Trichomonas vaginalis* sequences, as well as those from model eukaryotic and prokaryotic organisms. Sequences identified were validated through different approaches, including the biochemical purification of the *Leishmania* eIF3 complex followed by subsequent proteomic analysis, and used to investigate conserved and divergent features. Our results indicate a substantial degree of conservation of the eIF3 subunits in most eukaryotes whilst highlighting the occurrence of multiple instances of complex simplification. It also suggests a likely central role for the eIF3b, eIF3c and eIF3i subunits in eIF3 function.

## Results

### Bioinformatic identification of eIF3 subunits within selected genome sequences

A detailed *de novo* search was carried out for sequences corresponding to eIF3 subunits present within the proteomes of the four unicellular pathogens chosen for this study (*T. brucei*, *L. major*, *T. vaginalis* and *G. duodenalis*), as well as representative organisms used for comparison (described in the Methods section). Identification of these sequences was validated using as reference the annotated human eIF3 subunits. This validation generated the results shown in Table 1, providing an overview on the conservation of individual subunits throughout representative eukaryotic lineages. The human eIF3 subunits were also used for pair-wise comparisons aiming to evaluate the degree of identity/similarity between the sequences identified from the four target organisms and their mammalian counterparts, summarized in Table 2.

**Table 1:** **Summary of the search data for eIF3 subunits from the twelve organisms selected for this study**

Subunit	***H. sapiens***	***C. elegans***	***A. thaliana***	***A. niger***	***S. pombe***	***S. cerevisae***	***L. major***	***T. brucei***	***T. vaginalis***	***G. duodenalis***	***M. jannaschii***	***E. coli***
**eIF3a**	**X**	**X**	**X**	**X**	**X**	**X**	**X**	**X**				
**eIF3b**	**X**	**X**	**X**	**X**	**X**	**X**	**X**	**X**	**X**	**X**		
**eIF3c**	**X**	**X**	**X**	**X**	**X**	**X**	**X**	**X**	**X**	**X**		
**eIF3d**	**X**	**X**	**X**	**X**	**X**		**X**	**X**	**X**			
**eIF3e**	**X**	**X**	**X**	**X**	**X**		**X**	**X**				
**eIF3f**	**X**	**X**	**X**	**X**	**X**		**X** ^**+**^	**X** ^**+**^				
**eIF3g**	**X**	**X**	**X**	**X**	**X**	**X**	**X**	**X**				
**eIF3h**	**X**	**X**	**X**	**X**	**X**		**X**	**X**	**X**			
**eIF3i**	**X**	**X**	**X**	**X**	**X**	**X**	**X**	**X**	**X**	**X**	**X**	
**eIF3j**	**X**	**X**	**X**	**X**	**X**	**X**	**X**	**X**		**X**		
**eIF3k**	**X**	**X**	**X**	**X**			**X**	**X**				
**eIF3l**	**X**	**X**	**X**	**X**			**X**	**X**				
**eIF3m**	**X**	**X**	**X**	**X**	**X**							

The letter X means presence while empty box means absence in the various species, according to the bioinformatic analysis. **X** ^**+**^ symbol indicates the eIF3f homologues identified only after the biochemical analysis of the *Leishmania* eIF3 complex.

**Table 2:** **General features for each eIF3 subunit found from** ***L. major*** **,** ***T. brucei*** **,** ***T. vaginalis*** **and** ***G. duodenalis***

eIF3 subunit	(TriTrypDB) accession	Size (MW –KDa)	Comparison with human homologue		Gene product description at TriTrypDB
			% Identity (similarity)	% Query coverage	
eIF3a	LmjF.17.0010	774 (87.6)	20 (38)	51	Hypothetical protein, conserved
	Tb927.7.6090	762 (88.2)	22 (43)	61	Hypothetical protein, conserved
eIF3b	LmjF.17.1290	709 (80.8)	25 (42)	64	Translation initiation factor, putative
	Tb927.5.2570	696 (79.8)	22 (39)	91	Translation initiation factor, putative (EIF3B)
	TVAG_333940	608 (68.2)	19 (40)	96	Eukaryotic translation initiation factor 3 subunit, putative
	GL50803_15495	871 (98.5)	19 (37)	55	Hypothetical protein
eIF3c	LmjF.36.6980	731 (82.0)	24 (46)	44	Eukaryotic translation initiation factor 3 subunit 8, putative
	Tb927.10.8270/ Tb927.10.8290	740 (84.3)	24 (44)	74	Eukaryotic translation initiation factor 3 subunit 8, putative
	TVAG_184380	773 (90.0)	26 (47)	48	Hypothetical protein
	GL50803_24279	793 (89.5)	19 (39)	21	Hypothetical protein
eIF3d	LmjF.30.3040	531 (60.6)	24 (40)	90	Eukaryotic translation initiation factor 3 subunit 7-like, putative
	^1^ Tb927.6.4370	506 (57.9)	29 (46)	75	Eukaryotic translation initiation factor 3 subunit 7-like, putative
	TVAG_062640	464 (52.8)	25 (45)	63	Eukaryotic translation initiation factor 3 subunit, putative
eIF3e	LmjF.28.2310	405 (46.4)	28 (47)	93	Eukaryotic translation initiation factor subunit, putative
	Tb927.11.11590	413 (46.7)	27 (47)	72	Eukaryotic translation initiation factor subunit, putative
eIF3f	LmjF.25.1610	326 (36.7)	29 (50)	27	Hypothetical protein, conserved
	Tb927.3.1680	318 (35.1)	^2^~	10	Hypothetical protein, conserved
eIF3g	LmjF.34.2700	255 (28.8)	26 (47)	57	Hypothetical protein, conserved
	Tb927.4.1930	272 (31.3)	23 (42)	86	RNA-binding protein, putative (EIF3D)
eIF3h	LmjF.07.0640	335 (37.9)	21 (45)	65	Hypothetical protein, conserved
	Tb927.8.1170/ Tb927.8.1190	331 (36.4)	22 (40)	50	Hypothetical protein, conserved
	TVAG_105990	329 (38.0)	24 (44)	82	Eukaryotic translation initiation factor 3 subunit, putative
eIF3i	^1^LmjF.36.3880	352 (38.2)	29 (50)	96	Eukaryotic translation initiation factor 3 subunit 2, putative
	Tb927.11.9610	342 (37.7)	38 (57)	95	Eukaryotic translation initiation factor 3 subunit 2, putative (eIF-3 beta)
	TVAG_114460	371 (41.5)	27 (42)	88	Eukaryotic translation initiation factor 3 subunit, putative
	GL50803_13661	350 (38.4)	24 (43)	91	Eukaryotic translation initiation factor 3 subunit 2
eIF3j	LmjF.25.2120	211 (23.5)	22 (37)	79	Hypothetical protein, conserved
	Tb927.3.2220	220 (23.8)	25 (43)	53	Hypothetical protein, conserved
	GL50803_15546	261 (29.2)	23 (40)	98	Hypothetical protein
eIF3k	LmjF.32.2180	233 (26.3)	28 (39)	59	Hypothetical protein, conserved
	Tb927.11.15420	205 (23.1)	25 (46)	90	Hypothetical protein, conserved
eIF3l	LmjF.36.0250	633 (72.6)	25 (45)	65	Eukaryotic translation initiation factor 3 subunit L, putative
	Tb927.10.4640	488 (55.6)	25 (43)	87	Eukaryotic translation initiation factor 3 subunit L, putative

In addition, a comparison against *H. sapiens* eIF3 subunits was performed.

^1^In the TriTrypDB database the AUG from these sequences were likely misidentified since they generate peptides missing from orthologues in related species and were removed here.

^2^The Identity (Similarity) levels for this protein were not significant and were omitted from the table.

Orthologues to all thirteen subunits were found in lineages representing the three major groups of multicellular eukaryotes, metazoans (*Homo sapiens*), plants (*Arabidopsis thaliana*) and filamentous fungi (*Aspergillus niger*), confirming a substantial degree of conservation for the eIF3 complex. Using the bioinformatics approach, eleven orthologues for eIF3 subunits were found in both *L. major* and *T. brucei*, missing one PCI (eIF3m) and one MPN subunit (eIF3f) when compared to higher eukaryotes. A likely eIF3f orthologue was found experimentally in *Leishmania*, as discussed later in the text, and both *L. major* and *T. brucei* orthologues were included in Tables 1 and 2. The presence of a nearly complete eIF3 complex in trypanosomatids suggests that it would be structurally similar to the human eIF3 and contrasts with the substantial reduction in the number of eIF3 subunits found in *T. vaginalis* and *G. duodenalis*. Only five eIF3 subunits were found in *T. vaginalis* including eIF3b, eIF3d and eIF3i plus one subunit each with a PCI (eIF3c) and MPN (eIF3h) domain, comprising subunits from all three described mammalian eIF3 modules [8]. A similar result was also generated from the analysis with the *G. duodenalis* sequences, where only four subunits were identified. A minimal eIF3 then seems to be present in this organism, consisting of subunits eIF3b and eIF3i of the first mammalian eIF3 module plus a single PCI subunit (eIF3c) and, somewhat unexpectedly, an eIF3j orthologue. Another relevant observation is the identification of a putative eIF3i homologue from *M. jannaschii*, with this being the single eIF3 subunit which is found to have an Archaean homologue. Specific results generated in this study from the bioinformatic analysis of each eIF3 subunit are presented separately below.

### eIF3a

**Figure 1:**
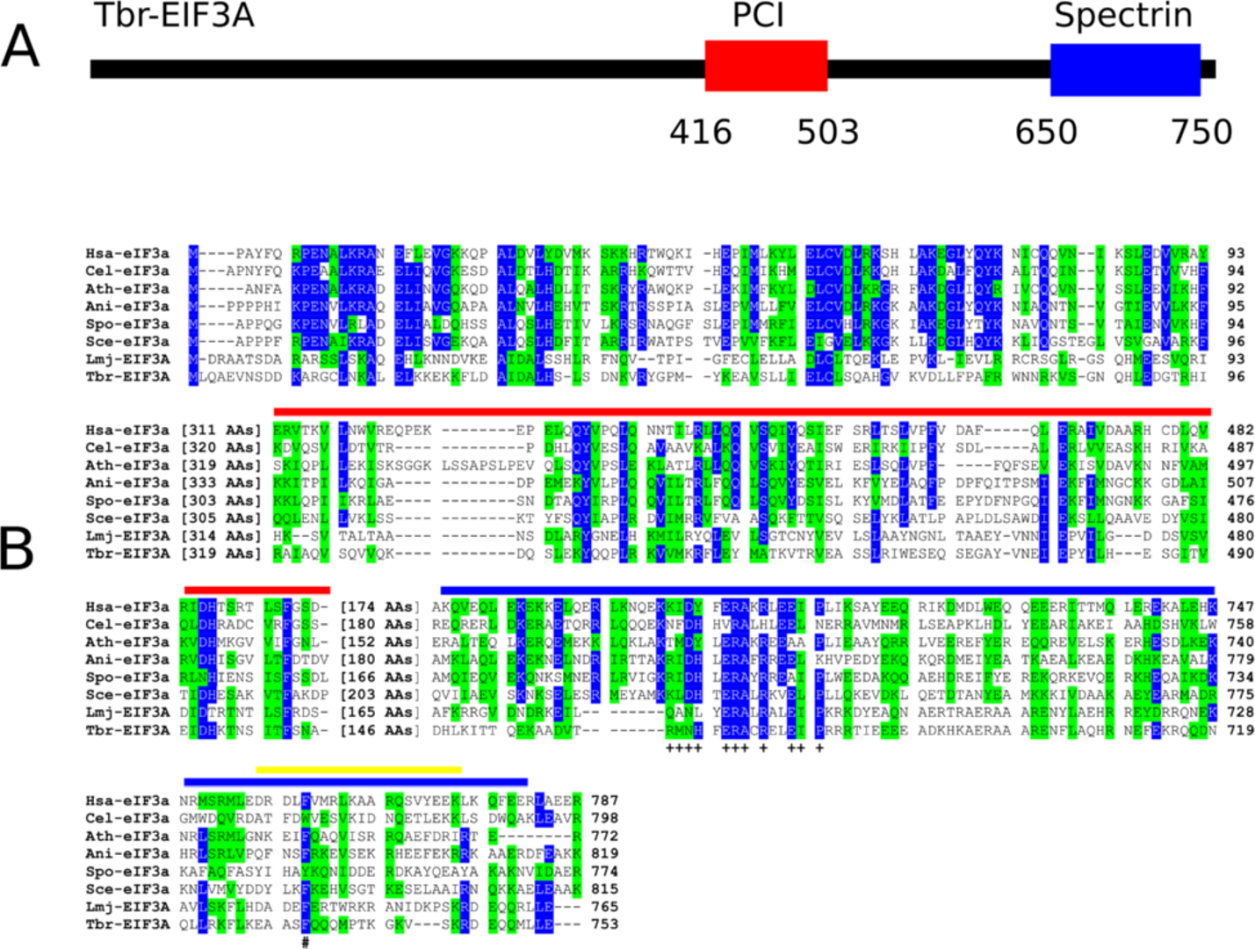
**Conserved and diverged features between the trypanosomatid EIF3As and various eukaryotic orthologues. A** Schematic representation of *T. brucei* EIF3A highlighting the PCI and Spectrin domains (red and blue boxes, respectively). **B** Amino acid sequence alignment comparing the N-terminus and the PCI and Spectrin domains from the various eIF3a homologues selected for this study. Amino acids identical in more than 60% of the sequences are shown with a blue background, while amino acids defined as similar, based on the BLOSUM 62 Matrix, in more than 60% of the sequences are highlighted with a green background. The red and blue lines define the PCI and Spectrin domains, respectively, whilst the yellow line indicates the segment implicated in the binding of human eIF3a to eIF3b and eIF3i [36]. The conserved residues within the Spectrin domain mentioned in the text are marked with “+”, whilst the “#” symbol marks the position of the conserved phenylalanine residue required for the interaction with human eIF3b.

The largest eIF3 subunit, eIF3a (also known as TIF32/RPG1), is characterized by the presence of a centrally localized PCI domain followed by a Spectrin repeat domain, typical of the Spectrin family of Actin-binding proteins (reviewed in [33]). eIF3a orthologues were identified from both *L. major* or *T. brucei* (named EIF3A), although only after searches carried out using the HMMs based approach, with both trypanosomatid polypeptides found being annotated as hypothetical proteins within the TriTrypDB database (Table 1). No equivalent sequences were found from either *G. duodenalis* or *T. vaginalis*, despite extensive searches using different bioinformatics approaches. When compared with the human eIF3a, the putative trypanosomatid orthologues display a low level of overall similarity (Table 2), but when they were included in a multiple alignment with other eIF3a orthologues a number of conserved amino acids, spaced throughout their length, are observed, as well as the characteristic PCI and Spectrin repeat domains (Additional file 1: Figure S1). Noteworthy is the presence of an extended C-terminus in the human protein, which is missing from the trypanosomatid orthologues and is much reduced in eIF3a sequences from other eukaryotes. Overall similarity between the different sequences is greater within their N-terminuses, implying a greater conservation in binding properties to the 40S subunit, eIF3c and eIFs involved in start codon recognition, all of which are associated with the N-terminal half of eIF3a [34, 35]. Conservation within the C-terminal halves, which include the PCI and Spectrin domains, is less pronounced (Figure 1A and B), although one of the longest conserved motifs in the alignment is found within the Spectrin domain, with seven out of ten amino acids strictly conserved between the trypanosomatid and human sequences. This Spectrin domain has recently been shown to mediate binding of human eIF3a to eIF3b and eIF3i [36] but, apart from the single phenylalanine residue (F760 in human eIF3a), shown to participate in the interaction with eIF3b and also found in the two trypanosomatid sequences, the overall similarity within the segment already implicated in binding to eIF3b and eIF3i is very low.

### eIF3b

**Figure 2:**
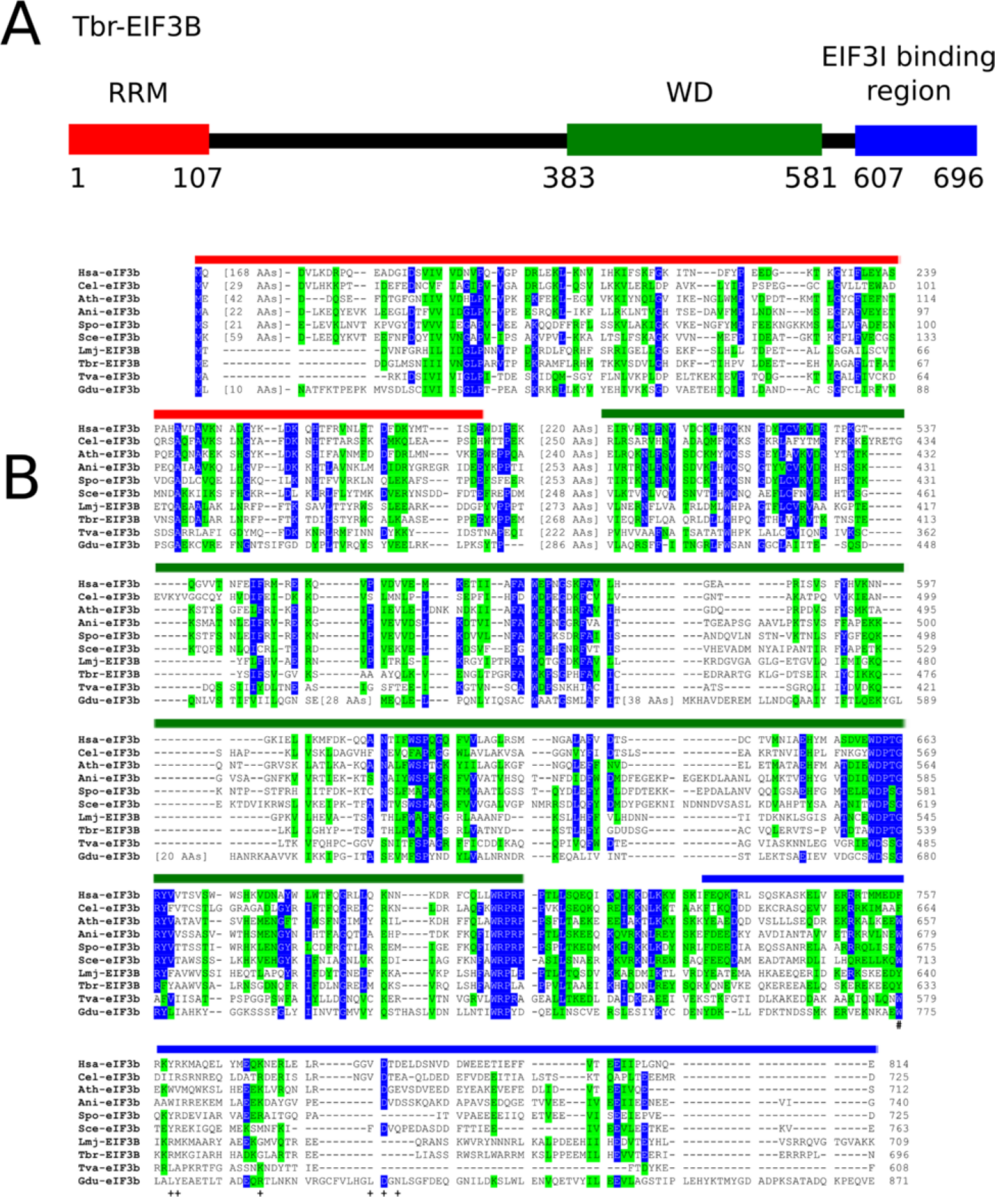
**Conserved and diverged features between eIF3b orthologues from various protozoan and multicellular organisms. A** Schematic representation of *T. brucei* EIF3B. The RRM and WD domains and the putative eIF3i binding region are indicated by red, green and blue boxes, respectively. **B** Amino acid sequence alignment comparing the RRM and WD domains and eIF3i binding region from eIF3b homologue from various protozoan and multicellular eukaryotes. The alignment was carried out as described for Figure 1 and the three segments analyzed are indicated by the red, green and blue lines, respectively. The “#” symbol marks the position of the conserved aromatic residue required for the interaction with yeast eIF3i [44], whilst the position of other residues seen to be involved in the interaction of eIF3b with eIF3i in yeast are indicated by the “+” symbol.

Also known as PRT1, eIF3b (reviewed in [4]) is a large scaffolding protein within the eIF3 complex, characterized by the presence of a non-canonical RRM domain [37]. The C-terminus of eIF3b consists of a very large segment defined as a WD repeat domain, which serves as a platform where proteins interact and participates in the formation of multiprotein complexes [38]. This WD repeat domain assumes a structure which has been recently solved and consists of a nine bladed β-propeller which interacts with the 40S ribosomal subunit [39]. As previously described [40], and in contrast to eIF3a, eIF3b orthologues are clearly identifiable in both *L. major* and *T. brucei*. The HMMs search also returned putative eIF3b orthologues for *T. vaginalis* and *G. duodenalis*, with the two proteins containing both RRM and WD repeat domains, but sharing little overall sequence identity with other homologues (Tables 1 and 2). When these proteins were analyzed by a multiple alignment, the conservation in sequence between the protozoa eIF3b orthologues and those from multicellular eukaryotes (fungi, mammals and plants) is more pronounced at the C-terminal WD repeat domain and within the protein’s center, whilst the conservation within the RRM domain is more restricted to its N-terminal end (Figure 2A and B and Additional file 1: Figure S2). The RRM of eIF3b has been involved in several protein-protein interactions required for eIF3b function, such as binding to eIF3a, eIF3j and eIF3e [37, 41, 42]. In contrast, its extreme C-terminus, including the end of the WD repeat domain, has been shown in *S. cerevisiae* to be involved in binding to eIF3g and eIF3i, with the interaction between eIF3b and eIF3i characterized in more detail [43, 44]. In the various protozoan homologues investigated here, and with the exception of a single aromatic residue implicated in this interaction (W713 in the yeast protein in the Figure) and found to be conserved in all eIF3b orthologues aligned, the C-terminus of eIF3b implicated in the interaction with eIF3i is poorly conserved.

### eIF3c

**Figure 3:**
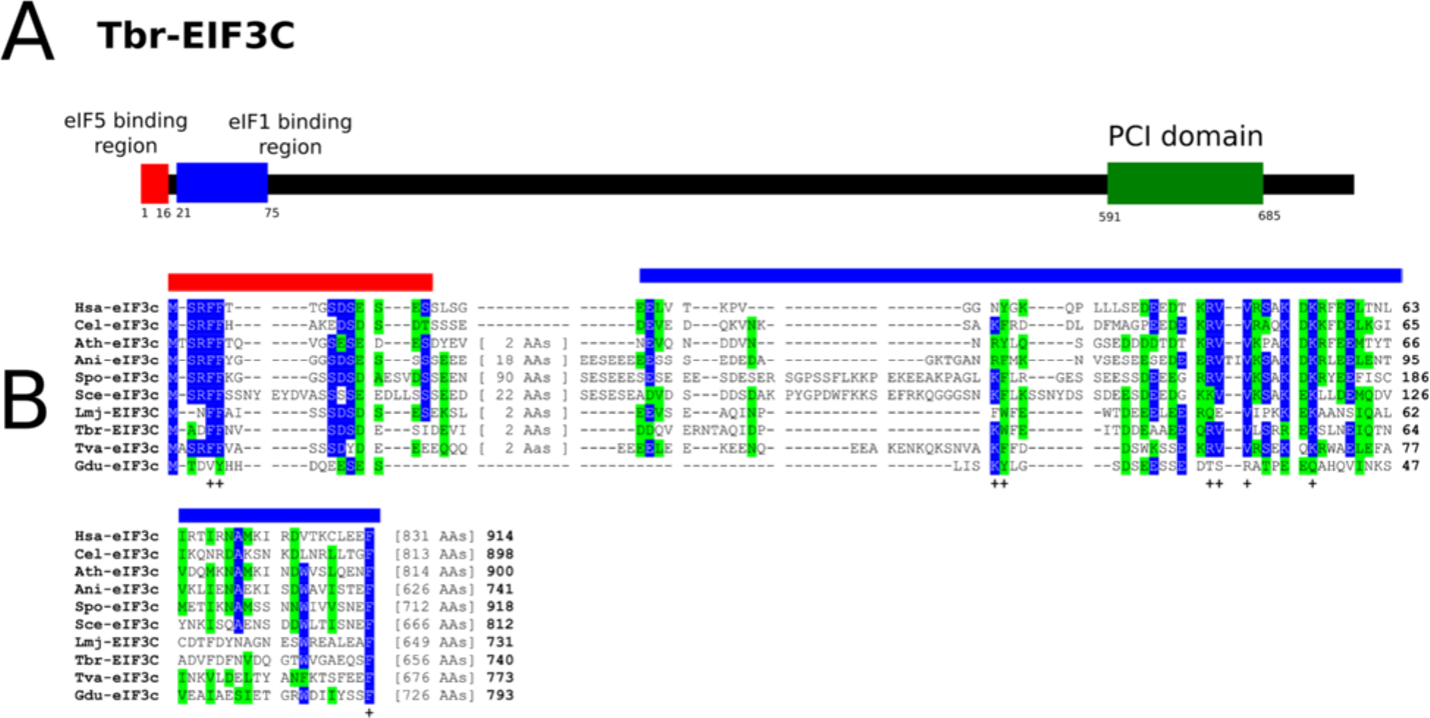
**Conserved elements at the N-terminal ends of eIF3c orthologues. A** Schematic representation of *T. brucei* EIF3C. The regions homologous to the eIF5 and eIF1 binding regions from yeast eIF3c and the PCI domain are indicated by red, blue and green boxes, respectively. **B** Amino acid sequence alignment comparing the putative eIF5 and eIF1 binding regions of eIF3c (according to [46]), carried out as described for Figure 1. The two regions are indicated by red and blue lines, respectively, and conserved positions are marked with the “+” symbol.

The third eIF3 subunit, eIF3c (NIP1), interacts with eIF3a and the two proteins form a dimer which is central to the eIF3 complex, since together they interact with each of the remaining PCI and MPN domain-containing subunits in human eIF3, as well as with the conserved eIF3b/eIF3g/eIF3i subcomplex [18]. eIF3c orthologues were clearly found in both trypanosomatids investigated, with two identical genes observed encoding the *T. brucei* protein. In contrast, putative orthologues for this eIF3 subunit from *T. vaginalis* and *G. duodenalis* were only identified through the HMM search and both are annotated as hypothetical proteins (Tables 1 and 2). When compared with the other two large eIF3 subunits, eIF3c is more conserved than eIF3a but not as much as eIF3b, although the degree of conservation is still low. Through the multiple alignment analysis, overall conservation between the various eIF3c homologues can be seen to be more concentrated at their last third, encompassing the PCI domain (Additional file 1: Figure S3), with little conservation within the segment which in yeast eIF3c has been implicated in the interaction with eIF3a (residues 157–370 of the yeast protein – [34]). A noteworthy feature for both trypanosomatid orthologues is the very glycine rich region at their C-terminuses, present also in plant eIF3c (and also in the human protein but with a reduced number of glycines), but missing from the fungi proteins as well as from the orthologues from *T. vaginalis* and *G. duodenalis*. Also of interest is the conservation of the N-terminal ends of most eIF3c orthologues, with two consecutive phenyalanines strictly conserved in all eIF3c sequences, with the exception of the *G. duodenalis* homologue. In yeast, the eIF3c N-terminus has been implicated in its binding to eIF5 [45, 46], so the conservation observed may highlight residues involved in this interaction. Likewise, conserved residues found within the segment implicated in binding to eIF1 in all eIF3c homologues studied, again with the exception of the *G. duodenalis* homologue, may pinpoint residues involved in this interaction (Figure 3A and B).

### eIF3d

The non-core subunit eIF3d (also called Moe1 or p66), from mammals, was studied very early on and seen to be able to cross-link to 18S RNA [47], with the protein’s segment responsible for the binding to RNA been mapped to within its first 120 residues [17]. Despite its absence in *S. cerevisae*, clear eIF3d orthologues are present in trypanosomatids with overall conservation similar to that observed for the previous subunits (Table 2). In addition a candidate for *T. vaginalis* was found during the HMMs search analysis, although no *G. duodenalis* eIF3d orthologue was identified. An alignment of the various eIF3d sequences reveals the existence of several sets of conserved amino acids distributed throughout their length which are good candidates for studies investigating eIF3d function (Additional file 1: Figure S4). These include various residues at the proteins’ N-terminal ends which, with the exception of the *T. vaginalis* orthologue, are strictly conserved and might be involved in the interaction with RNA, as well as the acidic C-terminus, absent from the *S. pombe* orthologue. eIF3d orthologues have been previously reported from trypanosomatids [40], but those in fact are possibly eIF3g orthologues (see below), containing a RRM domain, and do not align with true eIF3d proteins.

### eIF3e

**Figure 4:**
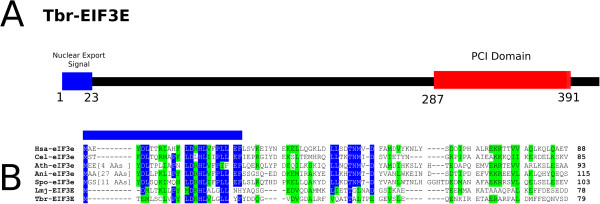
**Conservation of the human Nuclear Export Signal (NES) within various eIF3e orthologues. A** Schematic representation of *T. brucei* EIF3E. The NES and PCI domain are indicated by blue and red boxes, respectively. **B** Amino acid sequence alignment comparing the previously described NES, mapped to the very N-terminus of human eIF3e, and neighboring sequences, with similar sequences from trypanosomatids and other EIF3E orthologues.

eIF3e (p48, Int-6) is another non-core eIF3 subunit, characterized by a carboxi-terminal PCI domain (reviewed in [4, 48]), and which may have a role in regulating eIF3 function ( [49]; reviewed in [50]). It was first identified as the protein product of a gene which is the site of frequent integration of the mouse mammary tumor virus (*int-6*) and only later identified as an eIF3 subunit [51, 52]. eIF3e orthologues were also found in *L. major* and *T. brucei* which are in general more conserved than the previous eIF3 subunits (Table 2), but no orthologues could be found in either *T. vaginalis* or *G. duodenalis*. The comparison between the trypanosomatid sequences with other eukaryotic eIF3e orthologues reveals various conserved elements, within the PCI domain but also in the protein’s N-terminal half (Additional file 1: Figure S5). All orthologues share a very conserved segment which coincides with the N-terminus of the human protein (Figure 4) and where a previously reported Nuclear Export Signal (NES) has been mapped [53].

### eIF3f/eIF3h

The two eIF3 subunits containing the MPN domain, eIF3f and eIF3h, associate as a dimmer and both seem to interact with eIF3m, forming the third stable eIF3 module [8]. These two eIF3 subunits are each more closely related to the two MPN containing proteasome and COP9 signalosome subunits than to each other. eIF3f is more related to the proteasome subunit Rpn8 (also known as Mov34, p40 or subunit 7) and signalosome subunit Csn6, whilst eIF3h is more related to proteasomal subunit Rpn11 (also known as pad1 or p47) and signalosome subunit Csn5 (reviewed in [50]). In *S. cerevisae* these two eIF3 subunits are absent although the equivalent proteasomal subunits can be identified as well as a Csn5 homologue; in *S. pombe*, both eIF3 and proteasomal subunits are present as well as the signalosome Csn5; and in plants and animals both the eIF3 subunits and their two proteasomal and signalosome counterparts are found (also reviewed in [4, 5, 54–56]). BLAST searches of the *L. major* and *T. brucei* genome databases with the sequences of either eIF3f or eIF3h yielded proteins which were annotated as proteasomal subunits, with eIF3f finding as best hit a putative Rpn8 orthologue whilst eIF3h found a candidate Rpn11 orthologue. Nevertheless, through the HMMs search approach applied here, candidate eIF3h orthologues were found from both *L. major* and *T. brucei* (encoded by two neighboring genes), annotated as hypothetical proteins. As described below, the biochemical characterization of the *Leishmania* eIF3 yielded yet another conserved hypothetical protein which, upon blast searches against the non-redundant protein sequence databases from GenBank, displayed similarities against eIF3f orthologues. Despite not having an identifiable MPN domain it was considered a likely eIF3f orthologue and included in the analysis below. HMMs searches carried out for *T. vaginalis* and *G. duodenalis* yielded only two MPN containing homologues from *G. duodenalis*, annotated as potential proteasome subunits, and three from *T. vaginalis*, one of which was identified as a putative eIF3h orthologue.

**Figure 5:**
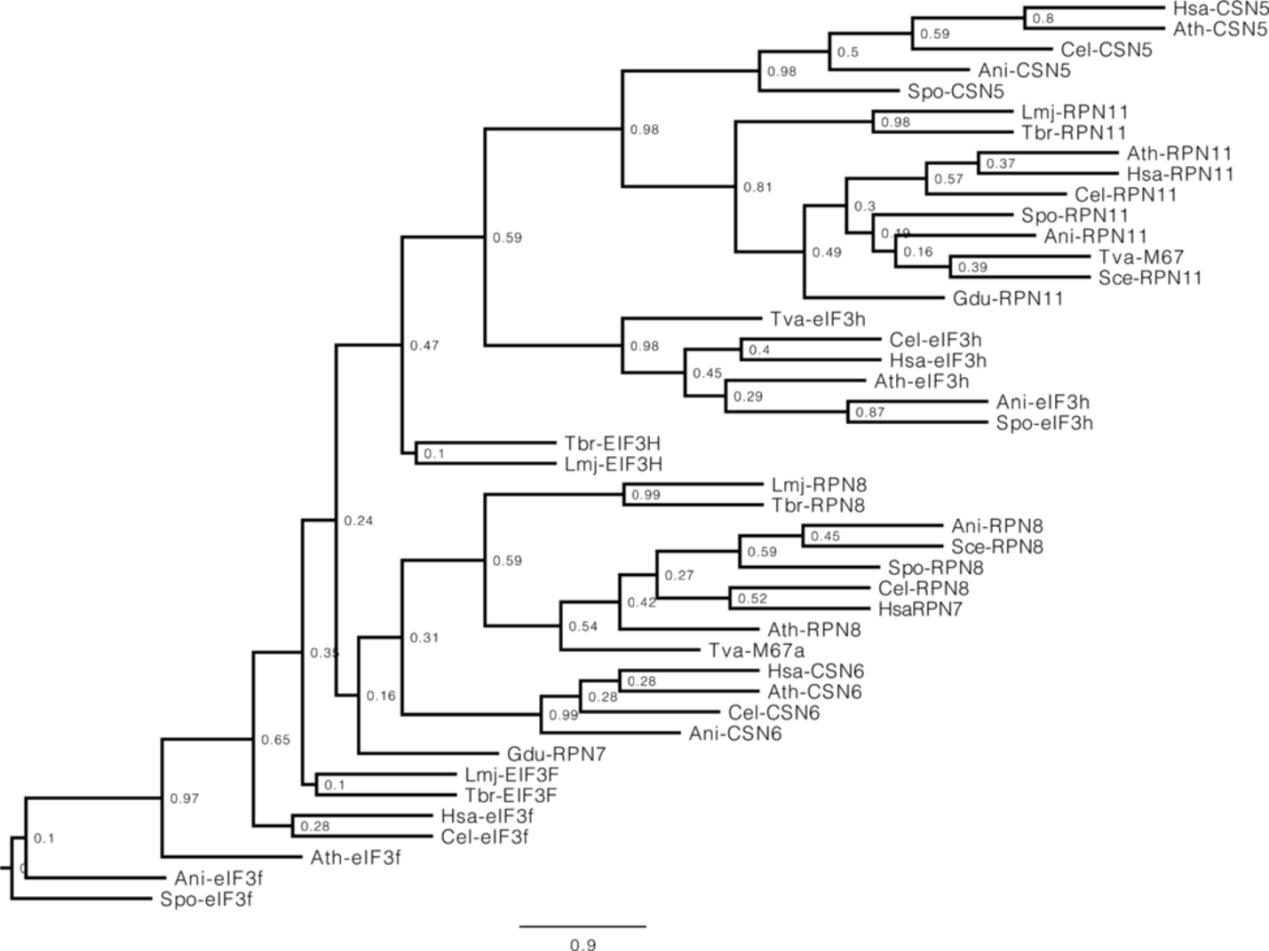
**Evolutionary relationship of the protozoan MPN containing proteins.** Maximum Likelihood tree based on the alignment of the MPN containing subunits from eIF3, proteasome and signalosome complexes from different eukaryotes, and the different MPN containing proteins identified in *L. major*, *T. brucei*, *T. vaginalis* and *G. duodenalis*. Bootstrap values are shown next to the respective branches (1,000 replicates).

Considering the high degree of similarity in sequence between the various MPN proteins, and to better evaluate their true relationships, a phylogenetic tree was built based on the alignment of multiple MPN containing proteins from the organisms chosen for this study. Sequences from both eIF3 subunits were included and compared with their putative trypanosomatid (eIF3f and eIF3h) and *T. vaginalis* (eIF3h only) orthologues, as well as with known orthologues from their counterparts found in the 26S proteasome and COP9 signalosome complexes and less defined MPN-containing proteins from the four protozoan genomes studied here. As shown in Figure 5, most of the eIF3f orthologues, including the putative trypanosomatid proteins, loosely group together as part of a large group which also includes the Rpn8/Rpn7 and Csn6 orthologues from various organisms. The latter proteins seem to be more conserved and form more robust subgroups which include likely Rpn8/Rpn7 orthologues from both trypanosomatids and *T. vaginalis* plus a less conserved MPN-containing homologue from *G. duodenalis* (Gdu-RPN7 in the figure). The various eIF3f sequences seem to be more divergent and their grouping is accompanied by low bootstraps, but an alignment carried out comparing known and putative eIF3f orthologues confirm the presence of conserved elements shared by all proteins and supporting their identification (Additional file 1: Figure S6A). For the eIF3h orthologues, they also group together, as part of a larger group which includes the various known Rpn11 and Csn5 orthologues as well as MPN containing Rpn11 orthologues from all four protozoans. Overall conservation for these proteins is greater than that observed for the eIF3f orthologues and related proteins and the grouping is validated by more robust bootstraps which includes the *T. vaginalis* eIF3h. The trypanosomatid eIF3h orthologues are more divergent and their positioning with the Rpn11/Csn5/eIF3h group has a low bootstrap, but when the various eIF3h sequences were aligned conserved elements were found throughout their lengths which substantiate their identification (Additional file 1: Figure S6B). Based on the evidence presented, Tables 1 and 2 include both trypanosomatid eIF3f and eIF3h orthologues and the *T. vaginalis* eIF3h in the comparisons carried out with the other parasite eIF3 subunits. Additional file 1: Figure S7 also provides a scheme for both *T. brucei* orthologues, highlighting the position of the MPN domain.

### eIF3g

The fourth subunit of the *S. cerevisiae* eIF3 core complex, eIF3g (TIF35 or p44 in humans), is an essential protein in both budding and fission yeasts species which has been implicated as a participant in translation reinitiation (reviewed in [4, 5]) and seen to be required for the scanning phase of the process [57]. Putative eIF3g orthologues were found in *T. brucei* and in *L. major* using the HMM search (Tables 1 and 2 - a schematic representation of the *T. brucei* orthologue is shown in Additional file 1: Figure S7), but none were identified from either *T. vaginalis* or *G. duodenalis*, implying that functional eIF3-like complexes may occur in the absence of this otherwise essential subunit. The two trypanosomatid proteins have been previously reported as possible eIF3d homologues [40] but this was likely a nomenclature error. The alignment with other eukaryotic eIF3g homologues (Additional file 1: Figure S8) shows that the conservation within the trypanosomatid sequences is mainly restricted to their C-terminal RRM domain, the major eIF3g feature, shown to be required for its RNA binding activity and for the protein’s role in mRNA scanning in yeast [57–59]. Nevertheless, minor segments of similarity are also observed within their N-terminal half, previously implicated in binding to eIF3i [60]. Noteworthy is the absence in the trypanosomatid proteins of the region encompassing the Zinc-Finger motif, previously implicated in the ability of plant’s eIF3g to bind to partners such as eIF4B and the viral Transactivator protein (TAV) of caulimoviruses [60, 61].

### eIF3i

**Figure 6:**
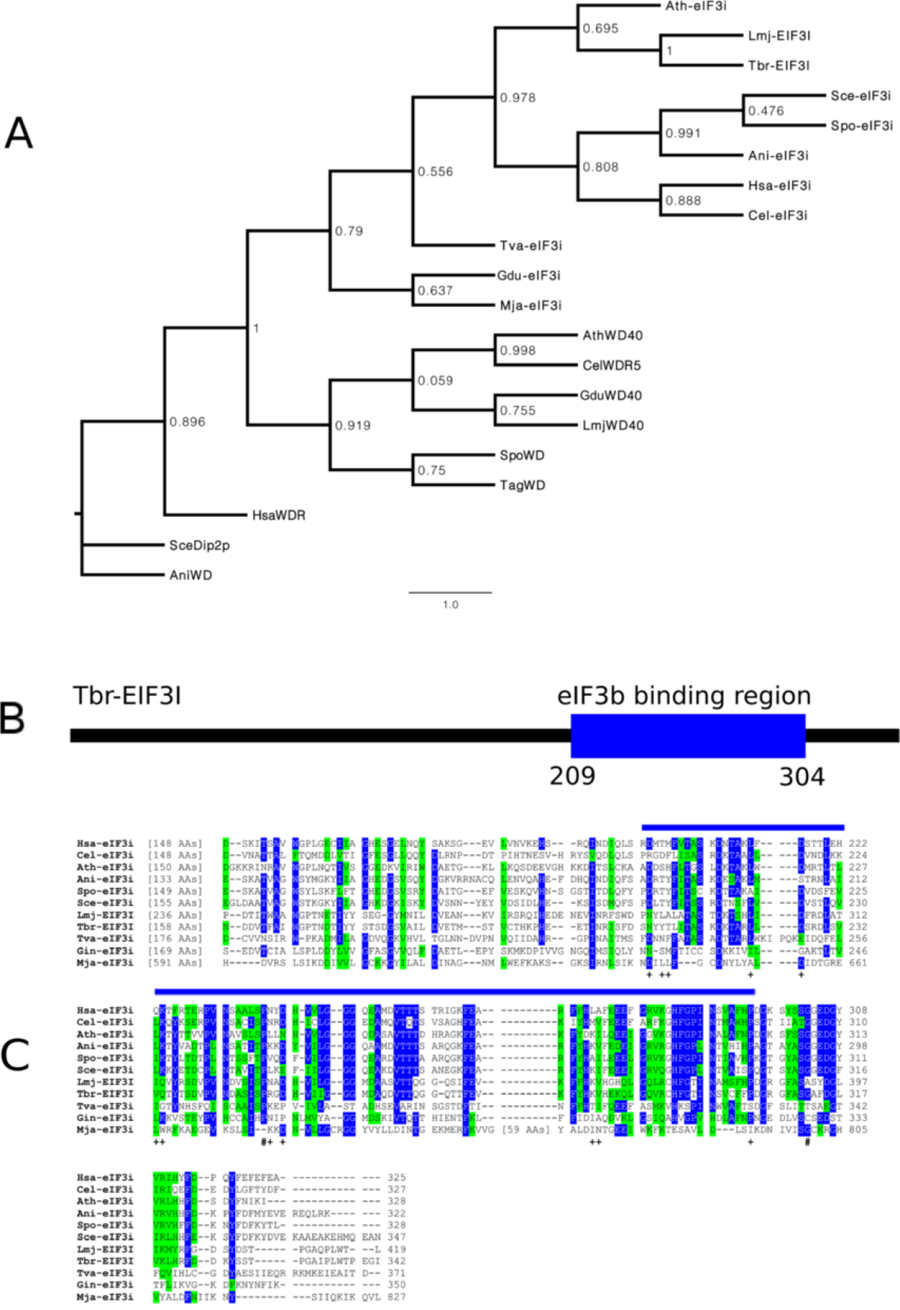
**Evolutionary relationship of selected eIF3i orthologues and conservation of the its eIF3b and eIF3g interacting elements. A** Maximum Likelihood tree based on the alignment of several eIF3i orthologues plus WD containing proteins from various protozoan and multicellular eukaryotes. **B** Schematic representation of *T. brucei* EIF3I. The position of the segment implicated in the binding to eIF3b [44] is indicated by a blue box. **C** Amino acid sequence alignment comparing the C-terminal half of eIF3i orthologues from the organisms selected for this study. The alignment was carried out as described for the previous figures. Residues seen to be involved in the interaction of eIF3i with eIF3b in yeast [44] are indicated by the “+” symbol, whilst residues which, when mutated, interfered in the interaction with eIF3g [43] are marked with “#”.

eIF3i (p36/TRIP1 in humans and TIF34/Sum1 in yeast) is another eIF3 core component conserved between yeast and humans, and thus also essential for translation *in vivo* (reviewed in [2, 5]). It is characterized by the presence of a WD repeat domain (as described for eIF3b), consisting of seven defined WD repeats which cover nearly all of its length and which are mostly conserved in different eukaryotic species [43]. eIF3i orthologues in trypanosomatids are clearly identifiable with size and features similar to other eukaryotic sequences and their similarity with the human protein is the highest among the eIF3 subunits (Table 2). *T. vaginalis* and *G. duodenalis* orthologues were also identified and, by using the HMM search, a putative *M. jannaschii* eIF3i orthologue was also found (Table 1). eIF3i then was the single eIF3 subunit from which a representative orthologue was found for this Archean organism and, with exception of *E. coli*, all other organisms investigated in this study were found to have eIF3i orthologues. The *M. jannaschii* homologue is only found in a restricted number of Archaen species and differs greatly in size and sequence from all other eukaryotic eIF3i orthologues but a phylogenetic tree built comparing these with their nearest WD containing homologues from various organisms shows a grouping with eIF3i (Figure 6A), although it is not clear at this stage if this grouping has any relevant implications regarding its function. Aligning the various eukaryotic eIF3i sequences reveal that they all have similar size and segments of similarities are seen throughout the sequences but the conservation is increased near the proteins’ N- and C-terminal ends (Additional file 1: Figure S9). The various amino acid residues mapped to the C-terminal end of yeast eIF3i and found to mediate the interaction with eIF3b [44] were also investigated (Figure 6B and C). From a total of twelve residues involved in the eIF3i-eIF3b interaction, highlighted in the Figure, eight are conserved in the eIF3i of trypanosomatids, most of which are not found in either of the *T. vaginalis* or *G. duodenalis* orthologues. Likewise, two residues implicated in the interaction with eIF3g [43] are also conserved in the trypanosomatid eIF3i orthologues.

### eIF3j

eIF3j (also called p35 in humans and HCR1 in *S. cerevisiae*) is the only non-core, non-essential eIF3 subunit found in *S. cerevisiae*. It is a highly conserved subunit which is loosely associated to the eIF3 complex and might play a role in mediating its binding to the 40S ribosomal subunit (reviewed in [2, 4, 5]). More recently eIF3j has been confirmed as non-essential in human cells [9] and has been implicated in events associated with control of translation termination and stop codon read-through in yeast [62]. Putative eIF3j homologues were found not only in both trypanosomatids investigated (annotated as hypothetical proteins) but also in *G. duodenalis* although no homologue was identified from *T. vaginalis* (Table 1). These eIF3j orthologues, however, display a very low level of identity when compared with better characterized eukaryotic eiF3j orthologues from mammals and yeast (Additional file 1: Figure S10), and they are all annotated as hypothetical proteins. A distinctive feature is the much conserved, negatively charged, C-terminal end in all proteins, which is somewhat more diverged in the *L. major* orthologue. The presence of eIF3j homologues in divergent eukaryotes highlight the relevant role it plays in translation initiation despite its non-essential nature in *S. cerevisae*.

### eIF3k, eIF3l and eIF3m

These are the most recently characterized of the eIF3 subunits, all three harboring a PCI domain and found in animal, plant and filamentous fungi species, but with two of them (eIF3l and eIF3k) absent from *S. cerevisae* and *S. pombe*[63] and the third, eIF3m (first called GA-17 in humans and Csn7b in *S. pombe*), missing from *S. cerevisae* but otherwise essential for translation in fission yeast [64]. eIF3k, the smallest non-core eIF3 subunit, is found in both trypanosomatid species studied, with sizes similar to those observed for this protein from other organisms, although no candidate *T. vaginalis* and *G. duodenalis* eIF3k sequences were identified (Tables 1 and 2). The conservation between the protozoan eIF3k orthologues and the human protein is very low but the sequences are conserved within the trypanosomatid family, encompassing its PCI domain, and the alignment with other eukaryotic proteins confirms the low similarity between the sequences (Additional file 1: Figures S7 and S11A). Clear orthologues in both *L. major* and *T. brucei* were also found for eIF3l (originally called HSPC021 in humans) with sizes and features similar to those present in other eukaryotes and overall conservation comparable to those observed for other eIF3 subunits (Tables 1 and 2). As for eIF3k, no likely orthologues were found in either *T. vaginalis* or *G. duodenalis* but the alignment of the trypanosomatid proteins with other eIF3l sequences reveals conserved elements throughout their length (Additional file 1: Figures S7 and S11B), including the previously described tetratricopeptide (TPR) repeat and PCI domain regions [65]. In contrast to the other PCI containing eIF3 subunits, no clear eIF3m orthologues were found within the trypanosomatid sequences and no *T. vaginalis* or *G. duodenalis* orthologues were found either. Using the HMM search, a single eIF3m homologue for both *T. brucei* and *L. major* was found (Tb927.10.15720 and LmjF.19.1120, respectively), which, however, clustered with 26S proteasome subunits upon sequence alignment and phylogenetic tree building with related sequences (data not shown).

### Biochemical characterization of the *Leishmania*eIF3 complex

**Figure 7:**
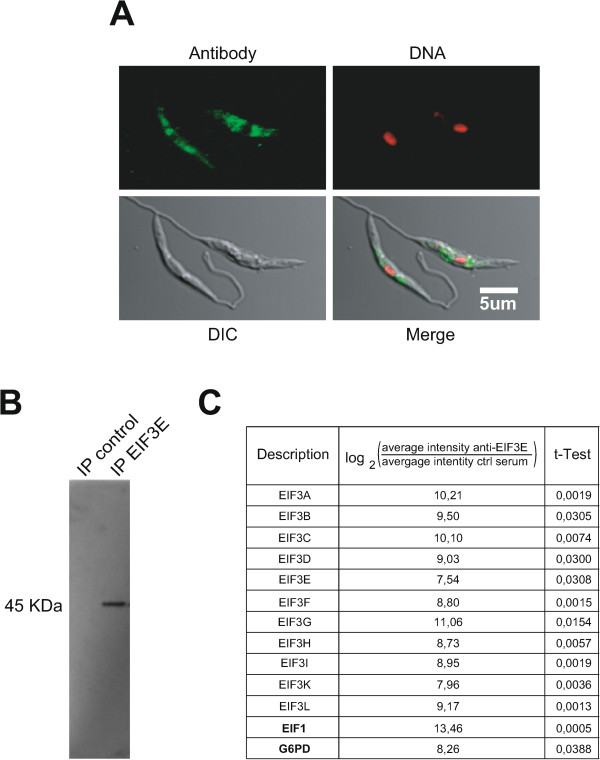
**Biochemical characterization of** ***Leishmania*** **EIF3. A** Subcellular localization of *Leishmania* EIF3E. The experiment was carried out through indirect immunofluorescence using the affinity purified anti-EIF3E antibodies. Where indicated, the cells were counterstained with TOTO-3 to locate the nuclear and kinetoplast DNA. **B** Immunoprecipitation (IP) of native EIF3E. IP reactions were set up using total *L. infantum* cytoplasmic extract and the affinity purified antibodies directed against *Leishmania* EIF3E, as well as the respective pre-immune control serum (IP Control). Precipitated immunocomplexes were then used in Western blot assays with the same anti-EIF3E antibodies used for the IP. **C** Summary of the mass-spectrometry analysis of the precipitated samples. A total of 13 polypeptides are shown which were specifically co-precipitated with the anti-EIF3E antibodies. The parameters shown in the table used to determine specificity for the IP reactions using the anti-EIF3E antibodies, always compared with the control IPs, are described in the Methods section. EIF1: translation initiation factor sui1. G6PD: Glucose-6-Phosphate 1-Dehydrogenase.

To validate the bioinformatic characterization of the various trypanosomatid eIF3 subunits, the sequence encoding the *L. major* EIF3E was amplified, cloned and expressed as an N-terminally his-tagged protein in *E. coli*. The resultant recombinant protein was then used to immunize rabbits and produce a specific polyclonal anti-serum which recognizes a single band of ~45 kDa in whole protein extracts of *L. major* and also *L. infantum* (not shown). Considering the high degree of conservation within the N-terminus of the various eukaryotic eIF3e orthologues (see Figure 4), and the postulated role for this segment in mediating the nuclear localization of the human protein [53], this anti-serum was first used to confirm the subcelullar localization of its orthologue in *L. major* promastigotas. As shown in Figure 7A, the *Leishmania* protein was found to strictly localize to the cellular cytoplasm, ruling out any nuclear localization but compatible with its role in translation initiation. The anti-serum was then used in immunoprecipitation assays, this time using total cell extracts from *L. infantum*, aiming to purify the whole eIF3 complex. An analysis of the precipitated samples through western-blots with the same serum confirmed the efficiency of the procedure for the eIF3e orthologue with none of it coming down in a control immunoprecipitation carried out with the pre-immune serum (Figure 7B). These samples were then submitted to mass spectrometry analysis in order to confirm the subunit composition of the *Leishmania* eIF3. Figure 7C summarizes the results derived from the mass spectrometry analysis of three sets of replicates comparing the anti-EIF3E antibodies with the pre-immune control. These results confirm the presence of 10 of the eIF3 subunits identified by the bioinformatic analysis and indicate the presence of a candidate eIF3f orthologue which cannot be identified by the HMMs based searches. They also highlight the strong association between the eIF3 complex and eIF1, the single other translation initiation factor which co-precipitated in this assay, whilst confirming the lack of association of the putative eIF3j orthologue with the trypanosomatid eIF3 complex.

## Discussion

The results from both *Leishmania* and *Trypanosoma* species are consistent with an early appearance of a fully functional eIF3 complex during the evolution of the eukaryotic lineages. The lack of identifiable orthologues to selected eIF3 subunits from *T. vaginalis* and *G. duodenalis* might be, at least in some cases, a consequence of too much divergence in sequence which prevented their proper identification purely through bioinformatic analysis. Indeed the identification, through biochemical approaches, of a putative eIF3f orthologue in trypanosomatids which was overlooked by the bioinformatic search supports this hypothesis. When compared with their *Leishmania* and *Trypanosoma* orthologues, the sequences of the eIF3 subunits found for *T. vaginalis* and *G. duodenalis* are in general less conserved, which is consistent with an earlier divergence from the main line of eukaryotic evolution or a faster evolution divergence rate for these organisms, as indicated by their classification within the supergroup Excavata [66]. Nevertheless, considering the lack of evidence for some of the most conserved eIF3 subunits (such as eIF3e or eIF3g) in both *T. vaginalis* and *G. duodenalis*, possibly a consequence of a secondary loss of subunits rather than reflecting an earlier evolutionary stage, the evidence presented indicates a much simplified eIF3 complex for these organisms. The absence of an eIF3a orthologue in these two protists is nonetheless striking, considering that it is the largest of the eIF3 subunits and the large number of interactions in which it is involved and which are critical for eIF3 function. Since an eIF3j orthologue is found in *G. duodenalis*, and considering the limited but consistent homology seen between the entire length of eIF3j and part of eIF3a [67], plus some functional overlap observed between the two proteins [68], a possible explanation would be for the *G. duodenalis* eIF3j to perform some or most of the functions carried out by the eIF3a subunit in other eukaryotes. For this organism at least, a simplification in some roles has been seen for other biological processes [69] and the data shown here is consistent with a simplified eIF3 complex based mainly on the eIF3b, eIF3c and eIF3i subunits.

A secondary loss of selected subunits from the eIF3 complex definitely seems to have happened for the budding yeast *S. cerevisae*, which lacks several subunits found in more primitive organisms (eIF3d, eIF3e, eIF3h, eIF3k and eIF3l) and appears to have suffered a drastic reduction in complexity during its evolution. Even the eIF3 from the fission yeast *S. pombe* seems to be in an intermediate situation between the *S. cerevisae* and filamentous fungi, since it includes five subunits (eIF3d, eIF3e, eIF3f, eIF3h and eIF3m) absent from the budding yeast eIF3 but is missing two subunits (eIF3k and eIF3l) which are present in most eukaryotes, including *A. niger* and both trypanosomatid lineages. Critical differences in eIF3 function between the *S. cerevisae* and human complexes have already been seen in the way it interacts with its eIF4G partner, an interaction critical for the mRNA recruitment by the ribosome (reviewed in [2]). In humans this interaction is mediated by a direct binding between eIF4G and three eIF3 subunits (eIF3c, eIF3d and eIF3e) [70] whilst in yeast this is done indirectly, through eIF5 [71]. Overall these results indicate a substantial flexibility in eIF3 function which has been differentially exploited by different organisms.

eIF3i has been shown to localize to the periphery of the eIF3 complex [8, 72], but is the most conserved eIF3 subunit, indicating both an ancient role in translation initiation (and an origin which may precede the evolution of early eukaryotes) and also a conservation of critical functions as part of the eIF3 complex. The presence of putative eIF3b and eIF3c orthologues in all eukaryotic organisms investigated thus far also highlights the central role that these proteins are likely to have within the eIF3 complex. In yeast, eIF3b-eIF3i-eIF3g form a ternary complex that interacts with eIF3c and eIF3a [73] and which has also been confirmed in mammalian cells [8, 36]. The ternary complex lacks any of the proteins belonging to the PCI/MPN octamer core of eIF3 but with eIF3a it forms the mammalian module i of eIF3, capable of maintaining on its own the ability to promote mRNA recruitment to the ribosomes [9]. Within this module, eIF3a seems to be critical for the formation and stability of the remaining eIF3 subunits and its function would have seem to be essential for any eIF3 function. Nevertheless, provided that it is indeed missing from *G. duodenalis* or *T. vaginalis*, the resulting eIF3 complex in these organisms would have to be stabilized solely by direct interactions between eIF3b and eIF3i and between eIF3b and eIF3c. Indeed the lack of conservation of residues in eIF3b which are involved in its interaction with eIF3i (despite a conservation of the eIF3b binding residues in eIF3i) might indicate significant divergence in this interaction in different organisms. Under this scenario, it might be possible that during its evolution the eIF3 complex started with a single PCI subunit (eIF3c) and no subunit containing a MPN domain. Other proteins with these specific domains were then acquired, with some at least deriving from the proteasome lid or signalsome subunits since four eIF3 subunits (eIF3f, eIF3h, eIF3k, eIF3l) have clear counterparts within these two complexes [13]. This model implies a significant contribution of eIF3i for the function of the whole eIF3 complex, at least in more primitive eukaryotes, however it is in disagreement with early evidence indicating that eIF3i and eIF3g are dispensable for several key functions of eIF3 in translation initiation in yeast [74] and that neither subunit is required for active complex formation in mammals [7], a discrepancy which needs to be resolved.

## Conclusion

The *in silico* and experimental data presented here highlight very relevant features regarding the eIF3 complex and its conservation in most, if not all eukaryotic lineages. The systematic approach carried out aiming to properly identify the different eIF3 subunits in two distinct trypanosomatid lineages, and also in *T. vaginalis* and *G. duodenalis*, provides a framework for future studies focusing on unique aspects of the translation machinery in these pathogens. Considering the lack of information regarding eIF3 structure and function in more divergent eukaryotes, the sequence analysis performed here can also help pinpoint conserved elements, in different subunits, so far overlooked but which might have relevant functional roles and are in need of a proper investigation. Subsequent experimental approaches to investigate and validate relevant differences found in comparison with other eukaryotes will be very useful not only to understand unique aspects of translation initiation, and its regulation, in these divergent eukaryotes but also may contribute to the understanding of the whole process, and how it can vary between different groups.

## Methods

### Organisms and sequences

In order to identify orthologues to subunits of the eIF3 complex in Trypanosomatids and lower eukaryotes, a range of predicted proteomes were downloaded for twelve organisms in February 25, 2013 including: *Homo sapiens* [taxid: 9606], *Caenorhabditis elegans* [taxid: 6239], *Arabidopsis thaliana* [taxid: 3702], *Aspergilus niger* [taxid: 425011], *Schizosaccharomyces pombe* [taxid: 284812], *Saccharomyces cerevisiae* [taxid: 559292], *Leishmania major* [taxid: 347515], *Trypanosoma brucei* [taxid: 185431], *Trichomonas vaginalis* [taxid: 412133], *Giardia duodenalis* [taxid: 5741], *Methanocaldococcus jannaschii* [taxid: 2190] and *Escherichia coli* [taxid: 1010810]. Within these organisms, four of them are excavates, two trypanosomatids (*L. major* and *T. brucei*) plus *T. vaginalis* and *G. duodenalis*. An Archea (*M. jannaschii*) and a bacterium (*E. coli*) species were also included, with the bacterium to be used as negative controls. Both trypanosomatid proteomes were downloaded from TritrypDB, whilst the *T. vaginalis* and *G. duodenalis* proteomes were downloaded from TrichDB and GiardiaDB, respectively, and all other proteomes were downloaded from NCBI ftp site. All accession numbers for the sequences included in the alignments and in the phylogenetic analysis described below (including not only the various orthologues to the eIF3 subunits but also other relevant sequences) are discriminated in Additional file 2: Table S1.

All likely eIF3 subunits from trypanosomatid species were named in capital letters following the proposed nomenclature for trypanosomatid proteins [75]. Abbreviations for the various organisms investigated are as follows: Hsa – *Homo sapiens*; Cel – *Caenorhabditis elegans*; Ath – *Arabidopsis thaliana*; Ani – *Aspergillus niger*; Spo – *Schizosaccharomyces pombe*; Sce – *Saccharomyces cerevisae*; Lmj – *Leishmania major*; Tbr – *Trypanosoma brucei*, Tva – *Trichomonas vaginalis*; Gdu– *Giardia duodenalis*, Mje – *Methanocaldococcus jannaschii*.

### Search for eIF3 subunits using Hidden Markov Models

To identify the eIF3 subunits within the proteomes of the selected organisms, all proteins derived from each chosen organism were clustered into orthologue groups. The OrthoMCL program [76], which uses the MCL (Markov Cluster) algorithm [77], was employed for this task. The human (*H. sapiens*) predicted proteome was then used as reference to search for the thirteen subunits of human eIF3 complex, and these proteins were used to recover the orthologue groups defined by the OrthoMCL tool. From a total of 213,686 protein sequences derived from the selected genome sequences, 23,271 orthologue groups were predicted based on OrthMCL analysis. When a search was made for subunits of the human eIF3 complex, 20 proteins were returned, and they were distributed into 13 orthologue groups.

All protein sequences present in each orthologue group were extracted, and these were used as input for the multiple alignment tool called MAFFT [78] (default settings). The alignments were manually inspected, and proteins, which were decreasing the alignment quality, were taken off from the data. Finally a multiple alignment of each orthologue group was used as input to *hmmbuild*, a program from the HMMER package, version 3.0 [79]. HMMER was used to build Hidden Markov Models (HMMs) based on multiple alignments, and the HMMs used to search for distantly related proteins within the various organisms’ proteomes using the *hmmsearch* tool (part of the HMMER package). A cutoff of 0.001 for hit significance (*e-value* < = 0.001) was used.

### Phylogenetic analysis of eIF3 subunits

In order to define how the identified orthologues for selected subunits (eIF3f, eIF3h and eIF3i) relate to each other as well as to closely related homologues, proteins recovered by the HMM searches were aligned by MAFFT (default settings). The alignments were automatically edited by Trimal [80] to keep just phylogenetically informative sites. The evolutionary model which best fits for each alignment was predicted by ProtTest [81]. Subsequently, the phylogenetic trees were built with PhyML tool using the Maximum Likelihood method [82]. The branch support for each tree was given by non-parametric bootstrap analysis using 1000 replicates.

### Cloning and protein expression methods

The *L. major* DNA fragment coding for its eIF3e orthologue was amplified by PCR from total genomic DNA flanked by restriction sites for the enzymes *BamH* I and *Xho* I (5′ primer - GTG *GGA TCC* ATG GAC ATG CTA ACG AAG CTG; 3′ primer - TG*C TCG AG*T TAA CGC ATA ACG GTG TCT AGC TT; restriction sites in italic) and cloned into the same sites of the expression plasmid pRSETa (Life Technologies®). Recombinant *L. major* EIF3E was expressed with an N-terminal histidine tag in *Escherichia coli* BL-21star cells (Life Technologies®) followed by purification with Ni-NTA Agarose beads (QIAGEN®) and quantification as previously described [83].

### Serum production and immunological procedures

The rabbit anti-serum generated against *L. major* EIF3E was produced through the immunization of a New Zealand white rabbit with the recombinant his-tagged protein using standard procedures. All the experimental procedures required for this immunization were approved by the “Ethics Committee for the Use of Animals on Research” from the Fundacao Oswaldo Cruz (CEUA-FIOCRUZ), license number L-053/08 to work with *Oryctolagus cuniculus*, and follow the ethical principles on animal experimentation defined by the Brazilian College of Animal Experimentation (COBEA). The serum generated was tested against both the recombinant protein and native *Leishmania* extracts through western-blotting and validated by comparison with the pre-immune serum. Antibodies derived from this serum, affinity purified against recombinant EIF3E, were then used to perform indirect immunofluorescence assays. These were carried out using the anti-rabbit IgG Alexa Fluor 488 as secondary antibody (Life Technologies®) and exponentially grown *L. major* promastigote cells, as described [84].

### Immunoprecipitation and proteomic analysis of *Leishmania*eIF3 subunits

Cytoplasmic extracts prepared from exponentially grown *Leishmania infantum* promastigotes were used in immunoprecipitation (IP) assays carried out with the affinity purified anti-EIF3E antibodies and the pre-immune serum used as negative control. Extract preparation and IPs were essentially performed as previously described [84], using 30 μl of protein A sepharose pre-incubated with either anti-EIF3E antibodies or the pre-immune serum (roughly 70 μg of total IgG in both), prior to the incubation with 200 μl (0.5 to 1.0 ODs at 260 nm) of the cytoplasmic extract. Proteins bound to the beads were eluted in SDS-PAGE and an aliquot validated for the presence/absence of *Leishmania* EIF3E through western blotting using the anti-EIF3E serum. For mass spectrometry (MS) both sets of eluted proteins were loaded unto 15% SDS-PAGE gels and allowed to migrate into the resolving gel, when the electrophoresis was interrupted. Gel slices containing the whole protein content loaded were excised and submitted to an in-gel tryptic digestion, followed by peptide elution and desalting at a homemade C18 stage-tip [85]. The peptides were analyzed by electrospray tandem mass spectrometry (ESI MS/MS), performed with an EASY nLC 1000 (Thermo Scientific), using a 15-cm fused silica emitter (75 μm inner diameter) in-house packed with reversed-phase ReproSil-Pur C18-AQ 3 μm resin (Dr. Maisch GmbH), connected to a LTQ Orbitrap XL ETD (Thermo Scientific) (mass spectrometry facility RPT02H PDTIS/Carlos Chagas Institute - Fiocruz Parana) mass spectrometer equipped with a nanoelectrospray ion source (Phoenix S&T). For data analyses, the MaxQuant platform (version 1.4.1.2) [86] was used for peak list picking, protein identification and validation. Protein identification was based on the *L. infantum* protein sequence databases (*L. infantum* JPCM5, version 6 from September 11, 2013 available at TriTrypDB). For validation, a minimum of six amino acids for peptide length and two peptides per protein were required. In addition, a false discovery rate (FDR) threshold of 0.01 (using the decoy database approach) was applied at both peptide and protein levels. To confirm the specificity of the IP assays, for each polypeptide, the ratio between the average intensities (from three independent experiments) generated for the anti-EIF3E and control IPs was first determined. The base 2 logarithms of the values produced were then calculated and only those >5 were considered. In addition, in order to have a statistical support for the results found, a t-Test was performed comparing the natural logarithm of the signal intensities from the triplicate experiments for the anti-EIF3E and control IPs and only those polypeptides having p-values <0.05 were considered.

### Availability of supporting data

The data sets supporting the results from this article are included within the main manuscript and within its two additional file(s).

## Electronic supplementary material

Additional file 1: Figure S1: Protein sequence alignment of eIF3a orthologues. Red and blue boxes represent the PCI and Spectrin domains, respectively. **Figure S2.** Protein sequence alignment of eIF3b orthologues. Red, green and blue boxes represent the RRM and WD domains and the eIF3i binding region, respectively. **Figure S3.** Protein sequence alignment of eIF3c orthologues. Red, blue and green boxes represent the eIF5 and eIF1 binding regions and the PCI domain, respectively. **Figure S4.** Protein sequence alignment of eIF3d orthologues. **Figure S5.** Protein sequence alignment of eIF3e orthologues. Blue and red boxes represent the NES and PCI domain, respectively. **Figure S6.** Protein sequence alignment of eIF3f and eIF3h orthologues. (A) Alignment of eIF3f orthologues. (B) Alignment of eIF3h orthologues. The red box represents the MPN domain. **Figure S7.** Schematic representation of the T. brucei EIF3F, EIF3G, EIF3H, EIF3K and EIF3L subunits. The domains/motifs are boxed and colored blue (EIF4F and EIF3H MPN domains), yellow (EIF3G RRM domain), red (EIF3K and EIF3L PCI domains) and brown (EIF3L TPR region). **Figure S8.** Protein sequence alignment of eIF3g orthologues. The red box represents the RRM domain. The Zinc Finger motif, when found, is highlighted (blue box). **Figure S9.** Protein sequence alignment of eIF3i orthologues. The red box represents the eIF3b binding region. **Figure S10.** Protein sequence alignment of eIF3j orthologues. **Figure S11.** Protein sequence alignment of eIF3k and eIF3l orthologues. (A) Alignment of the eIF3k orthologues. (B) Alignment of the eIF3l orthologues. The conserved TPR region and PCI domains are also highlighted (blue and red boxes, respectively). (DOCX 570 KB)

Additional file 2: Table S1: Accession numbers for the various protein sequences used. (DOCX 27 KB)
