# Supplementary material for: The translation initiation complex eIF3 in trypanosomatids and other pathogenic excavates – identification of conserved and divergent features based on orthologue analysis
Source: BMC Genomics. 2014 Dec 23;15(1):1175. doi: 10.1186/1471-2164-15-1175 (PMC4320536; doi:10.1186/1471-2164-15-1175)
Supplement: Supplementary file 1 — Additional file 1: Figure S1: Protein sequence alignment of eIF3a orthologues. Red and blue boxes represent the PCI and Spectrin domains, respectively. Figure S2. Protein sequence alignment of eIF3b orthologues. Red, green and blue boxes represent the RRM and WD domains and the eIF3i binding region, respectively. Figure S3. Protein sequence alignment of eIF3c orthologues. Red, blue and green boxes represent the eIF5 and eIF1 binding regions and the PCI domain, respectively. Figure S4. Protein sequence alignment of eIF3d orthologues. Figure S5. Protein sequence alignment of eIF3e orthologues. Blue and red boxes represent the NES and PCI domain, respectively. Figure S6. Protein sequence alignment of eIF3f and eIF3h orthologues. (A) Alignment of eIF3f orthologues. (B) Alignment of eIF3h orthologues. The red box represents the MPN domain. Figure S7. Schematic representation of the T. brucei EIF3F, EIF3G, EIF3H, EIF3K and EIF3L subunits. The domains/motifs are boxed and colored blue (EIF4F and EIF3H MPN domains), yellow (EIF3G RRM domain), red (EIF3K and EIF3L PCI domains) and brown (EIF3L TPR region). Figure S8. Protein sequence alignment of eIF3g orthologues. The red box represents the RRM domain. The Zinc Finger motif, when found, is highlighted (blue box). Figure S9. Protein sequence alignment of eIF3i orthologues. The red box represents the eIF3b binding region. Figure S10. Protein sequence alignment of eIF3j orthologues. Figure S11. Protein sequence alignment of eIF3k and eIF3l orthologues. (A) Alignment of the eIF3k orthologues. (B) Alignment of the eIF3l orthologues. The conserved TPR region and PCI domains are also highlighted (blue and red boxes, respectively). (DOCX 570 KB) [file 12864_2014_6982_MOESM1_ESM.docx]

Rezende et al.– Figure S1

**Hsa-eIF3a** M----PAYFQ RPENALKRAN EFLEVGKKQP ALDVLYDVMK SKKHRTWQKI -HEPIMLKYL ELCVDLRKSH LAKEGLYQYK NICQQVN--I KSLEDVVRAY 93

**Cel-eIF3a** M---APNYFQ KPEAALKRAE ELIQVGKESD ALDTLHDTIK ARRHKQWTTV -HEQIMIKHM ELCVDLKKQH LAKDALFQYK ALTQQIN--V KSLETVVVHF 94

**Ath-eIF3a** M-----ANFA KPENALKRAD ELINVGQKQD ALQALHDLIT SKRYRAWQKP -LEKIMFKYL DLCVDLKRGR FAKDGLIQYR IVCQQVN--V SSLEEVIKHF 92

**Ani-eIF3a** M---PPPPHI KPENVLKRAQ ELIAVGQAPA ALNVLHEHVT SKRTRSSPIA SLEPVMLLFV ELCVDLRKGK AAKDGLYQYK NIAQNTN--V GTIEVVLKKF 95

**Spo-eIF3a** M----APPQG KPENVLRLAD ELIALDQHSS ALQSLHETIV LKRSRNAQGF SLEPIMMRFI ELCVHLRKGK IAKEGLYTYK NAVQNTS--V TAIENVVKHF 94

**Sce-eIF3a** M----APPPF RPENAIKRAD ELISVGEKQA ALQSLHDFIT ARRIRWATPS TVEPVVFKFL EIGVELKKGK LLKDGLHQYK KLIQGSTEGL VSVGAVARKF 96

**Lmj-EIF3A** M-DRAATSDA RARSSLSKAQ EHLKNNDVKE AIDALSSHLR FNQV--TPI- -GFECLELLA DLCLTQEKLE PVKL-IEVLR RCRSGLR-GS QHMEESVQRI 93

**Tbr-EIF3A** MLQAEVNSDD KARGCLNKAL ELKKEKKFLD AIDALHS-LS DNKVRYGPM- -YKEAVSLLI ELCLSQAHGV KVDLLFPAFR WNNRKVS-GN QHLEDGTRHI 96

**Hsa-eIF3a** LKMAEEKTEA AKEESQQMVL ---------D IEDLDNIQTP ESVLLSAVSG EDTQDRTDRL LLTPWVKFLW ESYRQCL--D LLRNNSRVER LYHDIAQQAF 182

**Cel-eIF3a** LKLAEQRTED AQKQSIEKVE ---------E IGDLDQGDVP ERLLLAVVSG AAAQDRMDRT VLAPWLRFLW DSYRNCL--E LLRNNAQVEQ LYHTISRHSF 183

**Ath-eIF3a** LHLATDKAEQ ARSQADALEE AL-------D VDDLEADRKP EDLQLSIVSG EKGKDRSDRE LVTPWFKFLW ETYRTVL--E ILRNNSKLEA LYAMTAHKAF 183

**Ani-eIF3a** IELAEKKVTE AQAKADEIQS SLESAAPSSN VDDLEAIETP ETILLATVSG EQSRDRTDRA VVTPWLKFLW ETYRTVL--E ILKNNARLEV MYQTTALQAF 193

**Spo-eIF3a** IELANKRVQE AQEKADKISV EY-------- VDDLEATETP ESIMMSLVSG DLSKSRTDRA LVTPWLKFLW DAYRTVL--D ILRNNARLEV MYQLIANSAF 184

**Sce-eIF3a** IDLVESKIAS EQTRADELQK QE-------I DDDLEGGVTP ENLLISVYES DQSVAGFNDE AITSWLRFTW ESYRAVL--D LLRNNALLEI TYSGVVKKTM 187

**Lmj-EIF3A** VDSVMIRIRQ MCAKAKE--- ---------R AEAMQVNATE GDRMLAALNG ISLAALAEKR HIIPCHAALR RLIRHFCGNN TIGFAKKLTF IYNKTVRQFL 181

**Tbr-EIF3A** VNTTLDHLDK RCKWAYD--- ---------K VDETKAKRSE CDLILSSLSG ISIDQRVKDL YLVPAEKIVG EVAREMLTFN VIGHSGKLLP IYLETTEKLI 184

**Hsa-eIF3a** KFCLQYTRKA EFRKLCDNL- ----RMHL-- SQIQRHHNQS T----AIN-- ---------- LNNPESQSMH LETRLVQLDS AISMELWQEA FKAVEDIHGL 259

**Cel-eIF3a** TFCLRYQRRT EFRKLCDLL- ----RMHL-- NQIQKHQYAP NVNSFRVK-- ---------- LTSPESLGLM QDTRLIQLDT AIQMELWQEA YKSAEDVHGM 264

**Ath-eIF3a** QFCKQYKRTT EFRRLCEII- ----RNHL-- ANLNKYRDQR D----RPD-- ---------- LSAPESLQLY LDTRFDQLKV ATELGLWQEA FRSVEDIYGL 260

**Ani-eIF3a** QFCLKYTRKT EFRRLCELL- ----RNHV-- QNAAKYSAQM H----AIN-- ---------- LSDPDTLQRH LDTRFQQLNV AVELELWQEA FRSIEDIHTL 270

**Spo-eIF3a** QFCLKYQRKT EFRRLCELL- ----RSHL-- GNASKFSNAP H----SIN-- ---------- LNDAETMQRH LDMRFSQLNV AVELELWQEA FRSIEDIHSL 261

**Sce-eIF3a** HFCLKYQRKN EFKRLAEML- ----RQHLDA ANYQQSKSGN N----LVD-- ---------- LSDADTLQRY LDQRFQQVDV SVKLELWHEA YRSIEDVFHL 266

**Lmj-EIF3A** YMCKEFNAPA CVLDISSAVS RAVSHMAL-- DHLHDHDDAA -----SRNAV QELLKERAAF LTSTSAVTET VETLCCLLND ICSLRQYGGA FDVLSCLKRV 274

**Tbr-EIF3A** ELCRTYKFRA AIGHVADSFV RFFLRFLL-- YPIRPKTNKA Y----STRAA DALKIDRESF HRDVTAAQKT VSVFCQLLEA LIAVSNWQGA WRTLECFTKV 278

**Hsa-eIF3a** FSLS----KK PPK-PQLMAN YYNKVSTVFW KSGNALFHAS TLHRLYHLSR EMRK------ -------NLT QDEMQRMSTR VLLATLSIPI TPER-TDIAR 340

**Cel-eIF3a** MQLSKDKDKR TVK-PASYVN YYDKLALVFW KAGNSLFHAA ALLQKFIIYK DMKK------ -------SFT QDEAQEQATR VLLATLSIPE GSDSPSDLSR 350

**Ath-eIF3a** MCMV----KK TPK-SSLLMV YYSKLTEIFW ISSSHLYHAY AWFKLFSLQK NFNK------ -------NLS QKDLQLIASS VVLAALSIPP FDRA--QSAS 340

**Ani-eIF3a** LSLS----KR PAK-NVMMAN YYEKLARIFL VSENYLFHAA AFSRYYNLLR QSAAALAAGQ GTKKENPSVT EADMTKAASF VLLSALSIPV ISTS-RSRGA 364

**Spo-eIF3a** LTFS----KR APA-AVMLGN YYRKLIKIFL VCDNYLLHAA AWNRYF---- ---------- -------TFT NVQKPATANF VILSALSIPI IDAN-KLSGP 334

**Sce-eIF3a** MKIS----KR APK-PSTLAN YYENLVKVFF VSGDPLLHTT AWKKFYKLYS TNPR------ --------AT EEEFKTYSST IFLSAIST-- ---------Q 336

**Lmj-EIF3A** CDKV----QR NEEFVDVVSG AYETMSDIFW TCSVYNYHAY CLSVAASMKR QEKR------ ---------- ----GPLAAR AVLASLCVAD GDME------ 344

**Tbr-EIF3A** LAKT----KQ HEDFRKSQSD AYLVMATLFW ECSCYSFHAH CLLSAAFLAD DERK------ ---------- ----ESLLSR AVLAALCVPN IKGR-ESFAR 353

**410 420 430 440 450 460 470 480 490 500**

**....|....| ....|....| ....|....| ....|....| ....|....| ....|....| ....|....| ....|....| ....|....| ....|....|**

**Hsa-eIF3a** LLDMDGIIVE KQRRLATLLG LQAPP----- ---TRIGLIN DMV-RFNVLQ YVVPEVKDLY NWLEVE-FNP LKLCERVTKV LNWVREQPEK --------EP 422

**Cel-eIF3a** NLDIEDQHVA NMRLLSNLLR LPIAP----- ---TKNGILK EAA-RIGVPE AAGQTAKDLY KLLESN-FSP LKVAKDVQSV LDTVTR---- ---------P 427

**Ath-eIF3a** HMELEN-EKE RNLRMANLIG FNLEPKFEGK DMLSRSALLS ELV-SKGVLS CASQEVKDLF HVLEHE-FHP LDLGSKIQPL LEKISKSGGK LSSAPSLPEV 437

**Ani-eIF3a** LVDVDEVRKN KNTRLTNLLG MASPP----- ---TRAVLFK DAL-NKGLLK RARPEIRDLY NILEVD-FHP LSICKKITPI LKQIGA---- --------DP 442

**Spo-eIF3a** SIEAED-AKS KNARLALLLN LSKTP----- ---TRETLIK DAI-SRGVLS FCDQAIRDLY QILEVE-FHP LSICKKLQPI IKRLAE---- --------SN 411

**Sce-eIF3a** LDEIPSIGYD PHLRMYRLLN LDAKP----- ---TRKEMLQ SIIEDESIYG KVDEELKELY DIIEVN-FDV DTVKQQLENL LVKLSS---- --------KT 415

**Lmj-EIF3A** -LDPFGRYRE KNARIDELF- --ESP----- -ITTKTSLIA NLK-TKGVHT LASPDIVKIA ANLEDPNFSD FTGLHK--SV TALTAA---- --------NS 419

**Tbr-EIF3A** GSDS---FFQ KNEQIAKLLD LKEAP----- ---SRNFLVQ RMQ-QMQLLQ AAPKGVVAVV ELLRNEVFDG EASSRAIAQV SQVVQK---- --------DQ 429

**PCI DOMAIN**

**Hsa-eIF3a** ELQQYVPQLQ NNTILRLLQQ VSQIYQSIEF SRLTSLVPFV DAF-----QL ERAIVDAARH CDLQVRIDHT SRTLSFGSD- ---------- -LNYATREDA 505

**Cel-eIF3a** DHLQYVESLQ AVAAVKALKQ VSVIYEAISW ERIRKIIPFY SDL-----AL ERLVVEASKH RIVKAQLDHR ADCVRFGSS- ----DATLAG GVDECDNNEG 517

**Ath-eIF3a** QLSQYVPSLE KLATLRLLQQ VSKIYQTIRI ESLSQLVPF- ----FQFSEV EKISVDAVKN NFVAMKVDHM KGVVIFGNL- ---------- ---------- 511

**Ani-eIF3a** EMEKYVLPLQ QVILTRLFQQ LSQVYESVEL KFVYELAQFP DPFQITPSMI EKFIMNGCKK GDLAIRVDHI SGVLTFDTDV FS--SAKALH PGSAAGSAES 540

**Spo-eIF3a** DTAQYIRPLQ QVILTRLFQQ LSQVYDSISL KYVMDLATFE EPYDFNPGQI EKFIMNGNKK GAFSIRLNHI ENSISFSSDL FS-------N PIKSSDSVS- 503

**Sce-eIF3a** YFSQYIAPLR DVIMRRVFVA ASQKFTTVSQ SELYKLATLP APLDLSAWDI EKSLLQAAVE DYVSITIDHE SAKVTFAKDP FDIFASTASK EVSEEENTEP 515

**Lmj-EIF3A** DLARYGNELH KMILRYQLEV LSGTCNYVEV LSLAAYNGNL TAAEY-VNNI EPVILG---D DSVSVDIDTR TNTLSFRDS- ---------- ---------- 494

**Tbr-EIF3A** SLEKYQQPLR KVVMKRFLEY MATKVTRVEA SSLRIWESEQ SEGAY-VNEI EPYILH---E SGITVEIDHK TNSITFSNA- ---------- ---------- 504

**PCI DOMAIN**

**Hsa-eIF3a** PI-------- ---------- --GPHLQSMP S----EQIRN QLTAMSSVLA KALEVIKPAH ILQEKEEQHQ LAVTAYLKNS RKEHQRILA- RRQTIEERKE 580

**Cel-eIF3a** FT-------- ---------- -------GDD TQLGVEGVRN HLEAMYTRLR GLVEGLDAEK RRKEILKKIE GQVTSYEKNR PTEIERIHR- RKKMLENYKE 591

**Ath-eIF3a** ---------- ---------- -------GIE S----DGLRD HLAVFAESLS KVRAMLYP-- -VPSKASKLA GVIPNLADTV EKEHKRLLA- RKSIIEKRKE 576

**Ani-eIF3a** EV-------- ---------- GSVQRLQNTP A----EIARL QLTRLAKTLH VTCMYVDPSY -NEARLQAKR AALARAEAGA AKEHEETLA- RRVIIEKKKE 616

**Spo-eIF3a** ---------- ---------- -----LQSTP S----ELITS QLTRIAKSLS SVLMRFDTDF -CLLRKQQAE AAYERAQAGV EQERKAVIA- QRSLLELRRG 572

**Sce-eIF3a** EVQEEKEETD EALGPQETED GEEKEEESDP VIIRNSYIHN KLLELSNVLH DVDSFNNASY -MEKVRIARE TLIKKNKDDL EKISKIVDER VKRSQEQKQK 614

**Lmj-EIF3A** ---------- SKAKMLSYFN KIVANVDTVP A--------- -----SSLAT TSGRYSDFTH VAASSSKREV MPLAADLDAA RARSTSIYAL QKACMNSKAE 570

**Tbr-EIF3A** ---------- TKIKVLEAFD TLAQHVQLQP A--------- ---------- ---------- --ASRRKLDI KP--DHLRLV HERTRNLYNQ QQSCEEAAEQ 561

**Hsa-eIF3a** RLESLNIQRE KEELEQREAE LQKVRKAEEE RLRQEAKERE KERILQEHEQ IKKKTVRERL EQIKKTELG- -AK-AFKDID IEDLEELDPD FIMAKQVEQL 677

**Cel-eIF3a** NWERVKAEKT AAAATEQAKR EEAARAEEMK RLDEQNKESE RKRKQAEQDE IQKKIKQDQL YKMQQNAI-- -YQEIIKEKG LEQFRDMDPE QVLREQRERL 688

**Ath-eIF3a** DQERQQLEME REEEQKRLKL QKLTEEAEQK RLAAELAERR KQRILREIEE KELEEAQALL EETEK----- -RMKKGKKKP LLDGEKVTKQ SVKERALTEQ 670

**Ani-eIF3a** AATDALQRKQ REEETRKRIR TQQLQEAEKQ RLLDEHRERE KKRIKDEQDR IRQQELKKQL EELK------ -TGVKGIDIS ELDLNELDAN RLRAMKLAQL 709

**Spo-eIF3a** QADTLATQRE AELAAQRALK QKQESEAESL RVQEEINKRN AERIRREKEA IRINEAKKLA EELK------ -AK-GGLEVN AEDLEHLDAD KLRAMQIEQV 664

**Sce-eIF3a** HMEHAALHAE QDA-EVRQQR ILEEKAAIEA KLEEEAHRRL IEKKKREFEA IKEREITKMI TEVN------ -AK-GHVYID PNEAKSLDLD TIKQVIIAEV 705

**Lmj-EIF3A** RIE----EAK KSEDKKRERA VAERREQEEA KKEETQKAYI RKLYAEYNER QRQERGKEVL RKLRIKYPGF KVDESIVYRS AAGFEDELTR LLAAFKRRGV 666

**Tbr-EIF3A** RRK----DAK LREREKRSKE RAERIENEKK KKEAADLAKE SQGIAKYNEY VNQERRKLLL RRLREKYKGF LIKDIIAQKN SNDFVQEVTK LLADHLKITT 657

**SPECTRIN DOMAIN**

**Hsa-eIF3a** EKEKKELQER LKNQEKKIDY FERAKRLEEI PLIKSAYEEQ RIKDMDLWEQ QEEERITTMQ LEREKALEHK NRMSRMLEDR DLFVMRLKAA RQSVYEEKLK 777

**Cel-eIF3a** DKERAETQRR LQQQEKNFDH HVRALHLEEL NERRAVMNMR LSEAPKLHDL YEEARIAKEI AAHDSHVKLW GMWDQVRDAT FDWVESVKID NQETLEKKLS 788

**Ath-eIF3a** LKERQEMEKK LQKLAKTMDY LERAKREEAA PLIEAAYQRR LVEEREFYER EQQREVELSK ERHESDLKEK NRLSRMLGNK EIFQAQVISR RQAEFDRIRT 770

**Ani-eIF3a** EKEKNELNDR IRTTAKRIDH LERAFRREEL KHVPEDYEKQ KQRDMEIYEA TKAEALKEAE DKHKEAVALK HRLSRLVPQF NSFRKEVSEK RHEEFEKRRK 809

**Spo-eIF3a** EKQNKSMNER LRVIGKRIDH LERAYRREAI PLWEEDAKQQ AEHDREIFYE REKQRKEVQE RKHEQAIKDK KAFAQFASYI HAYKQNIDDE RDKAYQEAYA 764

**Sce-eIF3a** SKNKSELESR MEYAMKKLDH TERALRKVEL PLLQKEVDKL QETDTANYEA MKKKIVDAAK AEYEARMADR KNLVMVYDDY LKFKEHVSGT KESELAAIRN 805

**Lmj-EIF3A** DNDRKEIL-- ------QANL YERALRALEI PKRKDYEAQN AERTRAERAA ARENYLAEHR REYDRRQNEK AVLSKFLHDA DEFERTWRKR ANIDKPSKRD 758

**Tbr-EIF3A** QEKAADVT-- ------RMNH FERACRELEI PRRRTIEEEE ADKHKAERAA ARENFLAQHR NEFEKRQQDN QLLRKFLKEA ASFQQQMPTK GKV---SKRD 746

**SPECTRIN DOMAIN**

**Hsa-eIF3a** QFEERLAEER HNRLEE---- RKRQRKEERR ITYYREKEEE EQRRAEEQML KEREERERAE RAKREEELRE [ 4 AAs] -VKKLEEVER KKRQRELEIE [517 AAs] 1382

**Cel-eIF3a** DWQAKLEAVR NNRLAE---- RAEDRKKKRK EDAIQAKIAE ERKKREE--- ------EERA RLQVIE---- [ 3 AAs] -RQHNDGRGR REMENSVAMQ [274 AAs] 1076

**Ath-eIF3a** E--------R EERISKIIRE KKQERDIKRK QIYYLKIEEE RIRKLQEEEE ARKQ--EEAE RLKKVE---- [ 3 AAs] -KANLDKAFE KQRQREIELE [139 AAs] 987

**Ani-eIF3a** AAERDFEAKK MQRIKEVQER RRRERAEREE EERRRKEEEE RIRREEEERT AKE---EERR RVLAEEKAKR [ 4 AAs] --KRLDELAA KQKQREEEAE [144 AAs] 1052

**Spo-eIF3a** KAKNVIDAER ERQRKEIFEQ KLAEAIREAE EEAARAAEEE ANRELHEQEE AQKRAIEERT RAAREAKERE [ 4 AAs] -AEKLERQRR IQQERDEEI- [ 76 AAs] 932

**Sce-eIF3a** QKKAELEAAK KARIEEVRKR RYEEAIARRK EEIANAERQK RAQELAEATR KQREI-EEAA AKKSTPYSFR [ 20 AAs] DKAKLDMIAQ KQREMEEAIE [ 50 AAs] 964

**Lmj-EIF3A** EQQRLLE--- ---------- ---------- ---------- ---------- ------EEMR RLGGE----- --------- ---------- ---------- --------- 774

**Tbr-EIF3A** EQQMLLE--- ---------- ---------- ---------- ---------- ------MEKE RLQGK----- --------- ---------- ---------- --------- 762

**SPECTRIN DOMAIN**

Rezende et al.– Figure S2

**Hsa-eIF3b** MQ [167AAs] EADGIDSVIV VDNVPQ-VGP DRLEKL-KNV IHKIFSKFGK ITN---DFYP EEDG----KT KGYIFLEYAS PAHAVDAVKN ADGYK--LDK 257

**Cel-eIF3b** MV [38 AAs] IDEFEDNCVF IAGIPV-VGA DRLGKL-QSV LKKVLERLDP AVK---LYIP PSPEG---GC LGVLLTEWAD QRSAQFAVKS LNGYA--FDK 119

**Ath-eIF3b** ME [49 AAs] FDTGFGNIIV VDHLPV-VPK EKFEKL-EGV VKKIYNQLGV IKE-NGLWMP VDPDT--KMT LGYCFIEFNT PQEAQNAKEK SHGYK--LDK 132

**Ani-eIF3b** MA [32 AAs] LEEGLDTFVV IDGLPV-VPE ESRQKL-IKF LLRKLNTVGH TSE-DAVFMP LNDKN---MS EGFAFVEYET PEQAIAAVKQ LHGVP--LDK 115

**Spo-eIF3b** MS [31 AAs] KPVGYDTVVV IEGAPV-VEE AKQQDFFRFL SSKVLAKIGK VKE-NGFYMP FEEKNGKKMS LGLVFADFEN VDGADLCVQE LDGKQ--ILK 118

**Sce-eIF3b** MK [69 AAs] EEFNFDQYIV VNGAPV-IPS AKVPVL-KKA LTSLFSKAGK VVN---MEFP IDEAT--GKT KGFLFVECGS MNDAKKIIKS FHGKR--LDL 151

**Lmj-EIF3B** MT -------- -DVNFGRHIL IDGLPNNVTP DKRDLFQRHF SRRIGELLGG EKF--SLHLL TDPET---AL LSGAILSCVT ETQAEAALAK LNRFP--FTK 84

**Tbr-EIF3B** ME -------- DDGLMSNIII VNGLPARVTP EKRAMFLRHM TKKVSDVLGH DKF--TIHPV LDEET---EH VAGAFLTFAT VNSAEDALAR LNRFP--FTK 85

**Tva-eIF3b** MA -------- --RKIDSIVI VIGLPI-TDE SKIDQM-SGY FLNLVKPLDP ELTKEKIEVP TQDG----KT IGALFIVCKD SDSARRLAFI GDYMQ--FDK 82

**Gdu-eIF3b** ML [21 AAs] MVSDLSCIVI ISGLPT-PEA SKRKRLLKYV YEHIVKKSGD VAETEHIQIP LDAND---AC SGFCLIRFVN PSGAEKCVRE FNGNTSIFGD 105

**RRM DOMAIN**

**Hsa-eIF3b** QHTFRVNLFT DFDKYMT--- ISDEWDIPEK QPFKD---LG NLRYWLEEAE CRDQYSVIFE SGD----RTS IFW---NDV- -KDPV----- SIEER---AR 334

**Cel-eIF3b** NHTFTARSFK DMKQLEA--- PSDHWTTPEK QAYND---VG DLWWWLQNER CRDQ--FAIS HDKLGVPTVG VFT---NMK- GNDPELAGDA DKAER---AN 204

**Ath-eIF3b** SHIFAVNMFD DFDRLMN--- VKEEWEPPQA RPYVP---GE NLQKWLTDEK ARDQ--LVIR SGP----DTE VFW---NDTR QKAPE----- PVHKR---PY 209

**Ani-eIF3b** KHTLAVNKLM DIDRYGREGR IDEEYKPPTI EPFKE---KE HLRSWLGDAN ARDQ--FALY RGD----KVG VFW---NNK- SNPPE----- NVVDR---AH 194

**Spo-eIF3b** NHTFVVRKLN QLEKAFS--- TPDEFSFEER E-FKE---RE HLRSWLTDYY GRDQ--FISY YGN----RVS VNW---NRK- SDVPE----- QIVDR---EN 193

**Sce-eIF3b** KHRLFLYTMK DVERYNSDD- FDTEFREPDM PTFVP---SS SLKSWLMDDK VRDQ--FVLQ DDV----KTS VFW---NSMF NEEDS----- LVESR---EN 230

**Lmj-EIF3B** SAVLTTYRWS SLEEARK--- DDGPYVPPPT ANDDDEEEAE LVHNMAEDPD ARPQ--FLIK SGV----SFD CDWYWFNWE- KNEPDLYRRR KISKDDPLCR 174

**Tbr-EIF3B** TDILSTYRWC ALKAASE--- PPEEYKPPEM EQDTD---AD FAHTMAEDSM ARPQ--FFIK QGE----SFD VEWYWFNYT- TLKAELYRKP RPLKTDSVGQ 172

**Tva-eIF3b** KNRLRMFINN DYKKY----- IDSTNAPEQI AVAKPPSTPV EFSWYYTQPD MFDQ--IVYS AAG----FPH VCWFNHNTAK LQQIELPQYI QKSND----- 166

**Gdu-eIF3b** DYPLTVRQYS YVEELRK--- LPKSYTP--- QPYTPIDCSE RHRRLYFPLS GRIDHEFLLL T------KTN VSRCAFNGEN ISIINVQPCA DKLLD----- 191

**RRM DOMAIN**

**Hsa-eIF3b** WTET------ ---------- -----YVRWS PKGTYLATFH QRGIALWGGE ------KFKQ IQRFSHQGVQ LIDFSPCERY LVTFSPL--- [ 6 AAs ] 400

**Cel-eIF3b** WTET------ ---------- -----VFTWS PHGSYLSTIH KQGIILWGGK ------DYAR AHRFAHTNVQ YIDFSPWETY LVTYAAP--- [ 10 AAs ] 274

**Ath-eIF3b** WTES------ ---------- -----YVQWS PLGTYLVTLH KQGAAVWGGA D-----TFTR LMRYQHSMVK LVDFSPGEKY LVTYHSQ--- [ 9 AAs ] 279

**Ani-eIF3b** WTQL------ ---------- -----FVQWS PKGTYLASVH PQGVQLWGGP ------AFSK QKQFPHPFVQ LVEFSPGESY LTTWSARPIQ [ 13 AAs ] 270

**Spo-eIF3b** WTET------ ---------- -----YVQWS PMGTYLVSLH LRGIQLWGGE ------SWGM CARFLHPYVK FVDFSPNEKY LVSWSYEPVR [ 19 AAs ] 275

**Sce-eIF3b** WSTN------ ---------- -----YVRFS PKGTYLFSYH QQGVTAWGGP ------NFDR LRRFYHPDVR NSSVSPNEKY LVTFSTEPII [ 15 AAs ] 308

**Lmj-EIF3B** WSEVDRDNKK LVSGMVCSAL PVSRPLPVWS TYGSMVISQH EKGLRVWAGR ------SMRL HFEIT-MDIN AFMVSPCEKY IIVQT----- ---------- 252

**Tbr-EIF3B** WTEMDRRQKR LDPGLVYGAL TSVRPMPAWS TFGRIMVSQH MGGLKLWGGR ------KMHM LFEVTELDIK AFYISPQEKY LVVKS----- ---------- 251

**Tva-eIF3b** ---------- ---------- ------VFFT NDGSFFVTYS GNKLNFYAGE ------QWTH FTTLEFPDLR DYLNSPCGRF MLCKSLIPVE [ 6 AAs ] 230

**Gdu-eIF3b** ---------- ---------- ------VSYS PMGTYFIYHY EESIVLVHDN GTTITISHES DSQDTHTFIR SFKVSPCERF LLTFSYTANV [ 30 AAs ] 285

**Hsa-eIF3b** PQAI--IIWD ILTGHKKRGF [ 5 AAs ] AHWPIFK--W S-----HDGK FFA------- -----RMTL- DTLSIYE-TP SMGLLDKK-- ------SLKI 464

**Cel-eIF3b** KDSL--RIWD VRTGELKKAF [ 5 AAs ] PTWPFFR--W S-----FDEK YFACLKAPEK DKLEREQKI- NGISIFE-SE KFELYEGR-- ------PVNI 356

**Ath-eIF3b** KVEI--KVFD VRTGRMMRDF [ 17 AAs ] ASWPVFR--W AG---GKDDK YFA------- -----KLSK- NTISVYE-TE TFSLIDKK-- ------SMKV 357

**Ani-eIF3b** GKNI--IIWD IVTGKPLRSF [ 18 AAs ] VQWPAFK--W S-----ADEK YVA------- -----RMQQH QSISIYE-LP RMNLLGKT-- ------SVKI 348

**Spo-eIF3b** GKHC--FVWD IASGRILRSF [ 14 AAs ] VIWPIFK--W S-----ADDK YLA------- -----RVTVG QSISVYE-TP SLALVDKK-- ------TIKI 349

**Sce-eIF3b** GHQL--CIWD IASGLLMATF [ 7 AAs ] LKWPLVR--W S-----YNDK YCA------- -----RMVG- DSLIVHDATK NFMPLEAK-- ------ALKP 375

**Lmj-EIF3B** PKDI--SIIN LRTAKKIRTI [ 8 AAS ] DLWPIMR--F S-----ADDS LVVVCKTGYR PMDSAEVPE- GHLNIYV-SE TMKLLKGRGS SG---HSFAI 336

**Tbr-EIF3B** PKEV--SVWN IRLSKKIRVL [ 8 AAS ] DKWPIAR--Y N-----AEDE LVAISHACLE PMG-----Q- GKLFLYR-AE TMRALQVESN SETPVHSLVI 333

**Tva-eIF3b** PNGA--CIYD ILTGKKLVRI ---------- ---PLNRADF NQIMFGAGSH LIA------- ------MIE- KKVMLYK-AR EFKEVIEL-- ---------C 289

**Gdu-eIF3b** SKHITVSVWS IIERRILSTL [ 14 AAs ] LVRPEDRVLF S-----STGN LIL------- -----LMSN- EQISVYK--- --VQLTES-- ------AIYV 358

**Hsa-eIF3b** SGIKDFSWSP GG-------- -----NIIAF WVPED--KDI PARVTLMQ-- -----LPTRQ EIRVRNLFNV VDCKLHWQKN GDYLCVKVDR TPKGT----- 537

**Cel-eIF3b** ENIKQFEWSP TS-------- -----TVLAY YSECT--DAV PAEFGLLQ-- -----VPSMQ RLRSARVHNV ADAQMFWQKS GKRLAFYTMR FKKKEYRETG 434

**Ath-eIF3b** DNVVDICWSP TD-------- -----SILSL FVPEQGGGNQ PAKVALVQ-- -----IPSKV ELRQKNLFSV SDCKMYWQSS GEYLAVKVDR YTKTK----- 432

**Ani-eIF3b** DGVMDFEWSP ATVVRE---G VKQYEQLLCF WTPEI--GSN PARVALMS-- -----VPSKE IVRTRNLFNV SDVKLHWQSQ GTYVCVKVDR HSKSK----- 431

**Spo-eIF3b** DGVQNFEWCP VSDAL----G RDSKEQLLAY WTPEI--TNQ PARVALIS-- -----IPSKS TIRTKNLFNV SDCKLYWQSN GDYLCVKVDR HTKTK----- 431

**Sce-eIF3b** SGIRDFSFAP EGVKLQPFRN GDEPSVLLAY WTPET--NNS ACTATIAE-- -----VPRGR VLKTVNLVQV SNVTLHWQNQ AEFLCFNVER HTKSG----- 461

**Lmj-EIF3B** PGLYKAEWNP VV-------- ----GTQMAY VCELG--PNQ GWKAVVADMV VNEDGEVEQR VLNERNFLVA TRLDMLWHPA GTFLCVRVAA KGPTE----- 417

**Tbr-EIF3B** PGLKVAEWNP AV-------- ----GNQMAI LVQGG--SSE GWKIIIQNLV VKDD-VVRAE VIEQRNFLQA QRLDLLWHPQ GTHLVVKVTK TNSTE----- 413

**Tva-eIF3b** SDADSFSASP AT-------- -----KLVFT FRGQR--EAA PPRNAFYN-- -----TENGQ PVHVVAAFNA TSATATWHPK LALCCVIQNR IVKSC----- 362

**Gdu-eIF3b** ISLQEVLSYP NTTSLLFSPN GHGQDIVAIY QKSSD--KQK PSVCNVLNLN VPSHGRSGVR VLAQRSFF-I TNGRLFWSAN GGCLAIITE- -SQSD----- 448

**WD DOMAIN**

**Hsa-eIF3b** -----QGVVT NFEIFRM-RE KQ------VP VDVVE-M--- KETII--AFA WEPNGSKFAV [ 11 AAs ] FYHVKNN--- [ 5 AAs ] IKMFDK-QQA 611

**Cel-eIF3b** EVKYVGGCQY HVDIFEI-DK KD------VS LMNLP-L--- SEPFI--HFD WDPEGDKFCV [ 11 AAs ] VYKIEAN--- [ 6 AAs ] VSKLDAGVHF 515

**Ath-eIF3b** -----KSTYS GFELFRI-KE RD------IP IEVLE-LDNK NDKII--AFA WEPKGHRFAV [ 11 AAs ] FYSMKTA--- [ 8 AAs ] LATLKA-KQA 512

**Ani-eIF3b** -----KSMAT NLEIFRV-RE KG------VP VEVVDSL--- KDTVI--NFA WEPNGGRFVA [ 19 AAs ] FFAPEKK--- [ 9 AAs ] VRTIEK-KTS 518

**Spo-eIF3b** -----KSTFS NLEIFRI-RE KN------IP VEVVD-L--- KDVVL--NFA WEPKSDRFAI [ 18 AAs ] FYGFEQK--- [ 9 AAs ] IITFDK-KTC 516

**Sce-eIF3b** -----KTQFS NLQICRL-TE RD------IP VEKVE-L--- KDSVF--EFG WEPHGNRFVT [ 19 AAs ] FYAPETK--- [ 11 AAs ] VKEIPK-TFA 549

**Lmj-EIF3B** ---------- -YFLFHV-AE RN------VP ITRLS-I--- KRGYIPTRFA WQTGGDKFAV [ 18 AAS ] IFMIGKQ--- [ 4 AAS ] LHEVA--TSA 492

**Tbr-EIF3B** ---------- -YSIFSV-GV KS------AA AYQLK-V--- ENGLTPGRFA WKPSGPHFAV [ 18 AAS ] IYCIKKQ--- [ 3 AAS ] IGHYP--TSA 487

**Tva-eIF3b** -------DQS SIIIYDLTNE AS------IG SFTEE-I--- KGTVN--SCA WDPSNKHIAC [ 11 AAS ] IYDVDKQ--- [ 3 AAS ] VFQHPC-GGV 433

**Gdu-eIF3b** -----QNLVS TIFVIILQGN SE[26AAs]VK MEQLE-L--- PQNLYIQSAC WAATGSMLAF [ 60 AAs ] IFTLQEKYGL [ 30 AAs ] IKKIPG-ITA 628

**WD DOMAIN**

**Hsa-eIF3b** NTIFWSPQGQ FVVLAGLRSM ---NGALAFV DTS------- --------DC TVMNIAEHYM ASDVEWDPTG RYVVTSVSW- WSHKVDNAYW LWTFQGRLLQ 692

**Cel-eIF3b** NEVQFAPKGG WLAVLAKVSA ---GGNVYFI DTSLS----- --------EA KRTNVIEHPL FNKGYWDPTG RYFVTCSTLG GRAGADLGYR IFTFQGRELC 509

**Ath-eIF3b** NALFWSPTGK YIILAGLKGF ---NGQLEFF NVD------- --------EL ETMATAEHFM ATDIEWDPTG RYVATAVT-- SVHEMENGFT IWSFNGIMLY 592

**Ani-eIF3b** NAIYWSPKGR FVVVATVHSQ T--NFDIDFW DMDFEGEKP- EGEKDLAANL QLMKTVEHYG VTDIDWDPTG RYVVSSASV- WTHSMENGYN IHTFAGQTLA 614

**Spo-eIF3b** NSLFMAPKGR FMVAATLGSS T--QYDLEFY DLDFDTEKK- --EPDALANV QQIGSAEHFG MTELEWDPSG RYVTTSSTI- WRHKLENGYR LCDFRGTLLR 610

**Sce-eIF3b** NTVSWSPAGR FVVVGALVGP NMRRSDLQFY DMDYPGEKNI NDNNDVSASL KDVAHPTYSA ATNITWDPSG RYVTAWSSS- LKHKVEHGYK IFNIAGNLVK 648

**Lmj-EIF3B** THLFWAPRGG RLAAANFD-- ---KSLLHFF VLHDNN---- --------TI TDKNKLSGIS ATNCEWDPTG RYFAVWVSSI HEQTLAPQYR IFDYTGNELF 575

**Tbr-EIF3B** THLFWAPRGS RLVATNYD-- ---KSTLHFY GDUDSG---- --------AC VQLERVTS-P VTDTAWDPTG RFYAAWVSA- LRNSGDNQFR IFDLNGRELM 568

**Tva-eIF3b** SNITFSPAGR FFICDDIKAQ ---QPIVQFW DTE------- --------AG VIASKNNLEG VGRIEWDSSG AFVIISAT-- PSPGGPSWFA IYLLDGNQVC 513

**Gdu-eIF3b** SEVMFSPYND YLVALNRNDR ---KEQALIV INT------- --------ST LEKTSAEIEV VDGCSWDSSG RYLIAHKY-- GGKSSSFGLY IINVTGMVVY 708

**WD DOMAIN**

**Hsa-eIF3b** KNN----KDR FCQLLWRPRP -PTLLSQEQI KQIKKDLKKY SKIFEQKDRL SQSKASKELV ERRRTMMEDF RKYRKMAQEL YMEQKNERLE LR-----GGV 782

**Cel-eIF3b** RKN----LDR LAQFKWRPRP -PVKLSEQKQ REIKKNLKKT AAKFIKQDDD EKCRASQEVV EKRRKIMAAF DIIRSRNREQ LDATRDERIS LR-----NGV 689

**Ath-eIF3b** RIL----KDH FFQLAWRPRP -PSFLTAEKE EEIAKTLKKY SKKYEAEDQD VSLLLSEQDR EKRKALKEEW EKWVMQWKSL HEEEKLVRQN LR-------- 679

**Ani-eIF3b** EHP----TDK FKQFIWRPRP -PTLLSKEEQ KQVRKNLREY SKEFDEEDKY AVDIANTAVV ETRKRVLNEW AAWIRREKEM LAEEKDAYGV PE-------- 701

**Spo-eIF3b** EEM----IGE FKQFIWRPRP -PSPLTKEDM KKIRKKLKDY NRLFDEEDIA EQSSANRELA ARRRQLISEW QKYRDEVIAR VAEERAITGQ PA-------- 697

**Sce-eIF3b** EDI----IAG FKNFAWRPRP -ASILSNAER KKVRKNLREW SAQFEEQDAM EADTAMRDLI LHQRELLKQW TEYREKIGQE MEKSMNFKI- ---------F 733

**Lmj-EIF3B** KKA----VKP LSHFAWRPLP -PTLLTQSDV KKARDMIKTL VRDYEATEMA HKAEEQERID KERKSKEEDY IKRMKMAARY AEEKGMVQTR EE-------- 662

**Tbr-EIF3B** QKS----VRQ LSHFAWRPLA -PPVLTAAEI KHIQDNLREY SQRYQNEVKE QKEREEAELQ SKEREKEEQY KKRMKGIARH HADKGLARTR EE-------- 655

**Tva-eIF3b** KER----VTN VGRVLWRPRA GEALLTKEDL DAIDKEAEEI VEKSTKFGTI DLKAKEDDAK AAKIQNLQNW RRLAPKRTFG ASSNKNDYTT IE-------- 601

**Gdu-eIF3b** QSTHASLVDN LLNTIWRPYD -QELINSCVE RSLESIYKYC DENYDK--LL FDKTNDSSMK ERVEKNKAEW LALYEAETLT ADEQRTLNKN VRGCFVLHGL 805

**WD DOMAIN eIF3i BINDING REGION**

**Hsa-eIF3b** DTDELDSNVD DWEEETIEFF --------VT EEIIPLGNQ- ---------- ---------- -----E 814

**Cel-eIF3b** DTEA-QLDED EFVDEEITIA LSTS----KT QAPLTEEEMR ---------- ---------- -----D 725

**Ath-eIF3b** DGEVSDVEED EYEAKEVEFE DLID----VT EEIVQE---- ---------- ---------- -----S 712

**Ani-eIF3b** DVDSSKQAKD APAVSEDQGE TVVEE---IV EEIIEENEE- ---------- ---VI----- -----G 740

**Spo-eIF3b** --------IT VPAEEEEIIQ ETVEE---VI SEEIEPVE-- ---------- ---------- -----D 725

**Sce-eIF3b** DVQPEDASDD FTTIEE---- --------IV EEVLEETKE- ---------- ---KV----- -----E 763

**Lmj-EIF3B** -----QRANS KWVRYNNNRL KALPDEEHII HEDVTEYHL- ---------- ---VSRRQVG TGVAKK 709

**Tbr-EIF3B** -----LIASS RWSRLWARRM KSLPPEEMIL HEVVTEERI- ---------- ---ERRRPL- -----N 696

**Tva-eIF3b** ---------- ---------- ---------- ---FTDYKE- ---------- ---------- -----F 608

**Gdu-eIF3b** DGNLSGFDEQ GNILDKSLWL ENVQETVYIL EEVLAGSTIP LEHYKTMYGD ADPKSATADQ KPEQVE 871

**eIF3i BINDING REGION**

Rezende et al.– Figure S3

**Hsa-eIF3c** M-SRFFT--- ----TGSDSE S---ESSLSG ---------- ------EELV [ 6 AAs ] YGK----QP LLLSEDEEDT KRV--VRSAK DKRFEELTNL 63

**Cel-eIF3c** M-SRFFH--- ----AKEDSD S---DTSSSE ---------- ------DEVE [ 8 AAs ] FRD----DL DFMAGPEEDE KRV--VRAQK DKKFDELKGI 65

**Ath-eIF3c** MTSRFFTQ-- ----VGSESE D---ESDYEV [ 2 AAs ] ------NEVQ [ 6 AAs ] YLQ-----S GSEDDDDTDT KRV--VKPAK DKRFEEMTYT 66

**Ani-eIF3c** M-SRFFYG-- ----GGSDSE S---SSSEEE [18 AAs ] EESEEEEESS [12 AAs ] FMK-----N VSESEESEDE ERVTIVKSAK DKRLEELENT 95

**Spo-eIF3c** M-SRFFKG-- ----GSSDSD AESVDSSEEN [90 AAs ] SESEEESESE [28 AAs ] FLR---GES SEESSDEEEG RRV--VKSAK DKRYEEFISC 186

**Sce-eIF3c** M-SRFFSSNY EYDVASSSSE EDLLSSSEED [22 AAs ] SESESEADVD [27 AAs ] FLKSSNYDS SDEESDEEDG KKV--VKSAK EKLLDEMQDV 126

**Lmj-EIF3C** M--NFFAI-- ----SSSDSD S---ESEKSL [ 2 AAs ] ------EEVS [ 6 AAs ] WFE------ -WTDEEELEE RQE--VIPKK EKAANSIQAL 62

**Tbr-EIF3C** M-ADFFNV-- ------SDSD E---SIDEVI [ 2 AAs ] ------DDQV [ 9 AAs ] WFE------ -ITDDEAAEE QRV--VLSRR EKSLNEIQTN 64

**Tva-eIF3c** MASRFFVA-- ----SSSDYD E---EEEQQQ [ 2 AAs ] ----EEEELE [20 AAs ] FFD------ ---DSWKSSE KRV--VRSEK QKRWAELEFA 77

**Gdu-eIF3c** M-TDVYHH-- ----DQEESE S--------- ---------- ---------- [ 3 AAs ] YLG------ --SDSEESSE DTS--RATPE EQAHQVINKS 47

**eIF5 BINDING REGION eIF1 BINDING REGION**

**Hsa-eIF3c** IRTIRNAMKI RDVTKCLEEF ELLGKAYGKA KSIV--DKEG VPRFYIRILA DLEDYLNELW -EDKEGKKKM NKNNAKALST LRQKIRKYNR D--FESHITS 158

**Cel-eIF3c** IKQNRDAKSN KDLNRLLTGF DSLAKAYDKS KTVFQRQNVA NPRFYIRSLV EIEDYVNKLW -DDKDAKSAL SKNNAKALPP LRQKLKKYIK DQQLQDLVTD 164

**Ath-eIF3c** VDQMKNAMKI NDWVSLQENF DKVNKQLEKV MRIT--EAVK PPTLYIKTLV MLEDFLNEAL -ANKEAKKKM STSNSKALNS MKQKLKKNNK L--YEDDINK 161

**Ani-eIF3c** VKLIENAEKI SDWAVISTEF DKLNRQIVK- --IV--QSGP TPKIYVKTVA DLEDFVNGTI SKQKSSNKKM NASNAKGFNA VKQRIKKNNK D--YAVQIDK 188

**Spo-eIF3c** METIKNAMSS NNWIVVSNEF DHLNKVSQKC K-----EAGR NPPPYIEFLS ALDQKLES-- -ADKAFIKSL DAANGRAFNA LKQRVRKNNR Q--FQSDIDR 276

**Sce-eIF3c** YNKISQAENS DDWLTISNEF DLISRLLVRA QQ----QNWG TPNIFIKVVA QVEDAVN--- --NTQQADLK NKAVARAYNT TKQRVKKVSR E--NEDSMAK 215

**Lmj-EIF3C** CDTFDYNAGN ESWREALEAF KRMCDEVHTF VRKY--KVA- -PQGLQNCLQ DMPNLAEHL- -EGKGREDFA NRLEFKSLKE LVALVEETEK L--YKKELEE 154

**Tbr-EIF3C** ADVFDFNVDQ GTWVGAEQSF KELREKSDIH KRRF--QST- -PFPFLECLR NTPDLKTHL- ---SERESFA KPEEFRSLKG LIKAVEEAME T--YKDDIER 154

**Tva-eIF3c** INKVLDELTY ANFKTSFEEF QSLMKLHSKS QKAI--KQHG YPNFFIRGAS DIQQQVKDLL ---------- --PDNKDLRR FAQELEKFVV P--FQDKLNE 161

**Gdu-eIF3c** VEAIAESIET GRWDIIYSSF STLSDSFGKY HRML--RGEM -PRNWLLAMH RVLETFAEC- --SSSGASTI ARSNRKYFNA LKTFLEDEK- ---NRPVVSY 137

**eIF1 BINDING REGION**

**Hsa-eIF3c** YKQNPE---- -Q-SADEDAE[160 AAs]EI THAVVIKKL- -NE-ILQARG KKGTDRAAQI ELLQLLVQIA AENNLGEGVI VKIKFNIIAS LYDYNPNLAT 392

**Cel-eIF3c** YRVNPD---- -E-DGYETPE[118 AAs]EV THDVMIKKL- -NE-VMSARG KRTTDRNQHV ANLRKLLEVS EEKELGLGDU VKISFCIISA LFELNAKISD 366

**Ath-eIF3c** YREAPE---- -V-EEEKQPE[60 AAs ]EI TWDWVNKKF- -KE-IVAARG KKGTARFELV DQLTHLTKIA KTPAQK---- LEILFSVISA QFDVNPGLSG 300

**Ani-eIF3c** YRKDKDGFME AK-EEVARPV[23 AAs ]EV LRPLAVAHL- -XS-YVEXH- ---------- ---------- ---------- ---------- ---------- 248

**Spo-eIF3c** YRKDPEGFMK PA-ELNEIPK[31 AAs ]EI TPADIFKYL- -RA-IFEARG KKSTDRSEQI RLLEKLSTIA VTDYQR---- LRVKVALLAV RFDINTGSGQ 391

**Sce-eIF3c** FRNDPESFDK EP-TADLDIS[13 AAs ]QA VQEDFFTRL- -QT-IIDSRG KKTVNQQSLI STLEELLTVA EKPYEF---- IMAYLTLIPS RFDASANLS- 311

**Lmj-EIF3C** LAKGPENDDG AQ-DEDEDAG[ 1 AAs ]EL TEAEYAQIL- -EDISGSREV NLVGKVEKVI R------ACA RKGYTN--LE ISAMGIAVSA VLRRDSR-KL 235

**Tbr-EIF3C** LY---DEEDG DE-GDEEEAE[ 1 AAs ]EL TEDDIVKQL- -RE-SVTCTG KKASKYRKLA N------ECK RKGYKA--LQ ITTCGIVADA LLEEDNR-EV 231

**Tva-eIF3c** FAEHPENFQD EE-EEEDEFQ[69 AAs ]EV ITDQTAREEL DKDKLSRSKG KVITDTER-- --LSILLHRV EDEKLKNDIQ LEICFTIMQG SSEKA----- 312

**Gdu-eIF3c** VRAEYDAYLK KQLEDASEAQ[122 AAs]AL LQQNDNKSLL GQT--YDSTA QEPSLSTPFI TRLLTGLSSL SNLYELKDVI YYIYLSVFIS YYSTNGKLES 349

**Hsa-eIF3c** YMKPEMWGKC LDCINELM-- ---------D ILFANPNIFV [ 18 AAs ] RVRGCILTLV ERMDEEFTKI MQNTDPHSQ- --EYVEHLKD EAQVCAIIER 487

**Cel-eIF3c** HMEYETFMTT LQTVNSLL-- ---------D LLIGTDRVKL [ 18 AAs ] RIQGSILIAV QRLDGELAKI LQNADCHSN- --DYIEKLKA EKDMCSLIEK 460

**Ath-eIF3c** HMPINVWKKC VLNMLTIL-- ---------D ILVKYSNIV- [ 22 AAs ] RVWGNLVAFL ERVDTEFFKS LQCIDPHTR- --EYVERLRD EPMFLALAQN 397

**Ani-eIF3c** ------WMM- ---------- ---------- ---------- ---------- ---------- -----SLTRS LQHIDPHTA- --EYIDRLGD EKQLYANLVR 283

**Spo-eIF3c** YMPIDQWNAA LTELHSIL-- ---------D IFDANPKIVI [ 25 AAs ] QVQGSVVSFL ERLDDEFTRS LQMIDPHTP- --EYIDRLKD ETSLYTLLVR 492

**Sce-eIF3c** YQPIDQWKSS FNDISKLL-- ---------S ILDQTIDTYQ [ 23 AAs ] RILGSIFSFV ERLDDEFMKS LLNIDPHSS- --DYLIRLRD EQSIYNLILR 410

**Lmj-EIF3C** LVSSDTWERA FKWGAKFF-- ---------S RMIAATNVRF [ 12 AAs ] VVPGGIHGFL TYLHTELVNK SKFEEVASQ- --EYLKIITF ENELAVLADR 323

**Tbr-EIF3C** YVSTKTWAKS CDTLEECF-- ---------G LIVENPGIRL [ 13 AAs ] FIKGGLHALL QSLSKHLRRI TQFKDGIPS- --DYIEIVHL ENRLVAIADA 320

**Tva-eIF3c** -IPLTDWRLV LNILPKF--- ---------- ---------- [ 8 AAs ] -----LTPLF ERLSRDFW-- ARSVDPRTTF NPETNQLHQL QAEFNTSLTR 379

**Gdu-eIF3c** AI----FRTV ANYAKNLLRD GCSATVRYGD VLSTCSNASK [ 6 AAs ] ------LTLV PTCSDDVAPV PQSSELLRA- --EFTRTFPE ASKIFTTYLA 432

**Hsa-eIF3c** VQRYLEEK-- ------GTT- [ 7 AAs ] RIYLLRILHT YYKFD--Y-K [ 45 AAs ] RIRTCA-ILC HIYHHALHSR WYQARDLMLM SHLQ-DNIQH 601

**Cel-eIF3c** AEKYVELR-- ------NDSG [ 12 AAs ] KVYMMRIEHA YYKYQ----- [ 25 AAs ] RLRQRA-MLC HVYYLAVHDK WHRARDLLLM SHMQ-AIVDH 558

**Ath-eIF3c** IQDYFERM-- ------GDF- [ 7 AAs ] KVALRRVEAI YYKPQEVY-D [ 62 AAs ] RTKARA-MLC DINHHALMDN FVTARDLLLM SHLQ-DNIQH 531

**Ani-eIF3c** TQVYVEGL-- ------TQSE [ 14 AAs ] RVVMRRLEHI YFKPSQVV-T [ 44 AAs ] IIRARA-MLC QIYFLALHDQ YYRSRDLMLM SHLT-ENIAN 406

**Spo-eIF3c** SQGYLERI-- ------GVVE [ 7 AAs ] RLIMRRLDRV YYKPEQVI-R [ 44 AAs ] LLRTRA-MLC HIYHEALQNR FYKARDMLLM SHLQ-DSVHA 608

**Sce-eIF3c** TQLYFEAT-- ------LKDE [ 12 AAs ] RPFVKRLDHI YYKSENLI-K [ 47 AAs ] AVQKRA-ILY NIYYTALNKD FQTAKDMLLT SQVQ-TNINQ 534

**Lmj-EIF3C** ALGYYQAR-- ------KRI- [ 9 AAs ] ISILFDI--- ---------- [ 46 AAs ] VALSASGVCH VAYQYGLRGL YREGRDYLLR TGVV-NSIAV 432

**Tbr-EIF3C** LFGYYRDN-- ------SRG- [ 7 AAs ] CQILVDI--- ---------- [ 45 AAs ] ESKSLA-LLH LVYQMGLEGK YREGRDLIRR SGGA-EKLCN 425

**Tva-eIF3c** FCDLLEAD-- ------KKY- [ 7 AAs ] RLQIIMLENC YHELAPSFED [ 53 AAs ] SLKVRA-SLY YAIHLAYNGH PRQAQQLV-- -GVL-PDIPP 502

**Gdu-eIF3c** GHVFFRSKYL YLLYVAGTQR [ 18 AAs ] AIPLPEI--- ---TEEVTRK [ 52 AAs ] ELSPYTYDLF MSEQERTVED ARKVRDSRHT DEVSLLALNH 566

**Hsa-eIF3c** ADPPVQILYN RTMVQLGICA FRQGLTKDAH NALLDIQSSG RAKELL-GQG LLLRS-LQER NQEQEKVERR RQ-VPFHLHI NLELLECVYL VSAMLLEIPY 698

**Cel-eIF3c** SDVDTQILYN RTICQLGLCA FRHGFIREAH QGLSEIQNTQ RAKELL-AQA VGTRQ--HEK TAEQEKIDRS RQ-VPYHMHI NVELMECVYL ICSMLLEIPH 654

**Ath-eIF3c** MDISTQILFN RTMAQLGLCA FRAGMITESH SCLSELYSGQ RVRELL-AQG VSQSR-YHEK TPEQERMERR RQ-MPYHMHL NLELLEAVHL ICAMLLEVPN 628

**Ani-eIF3c** FDVSTQILFN RTLVQIGLCA FRAGLIYEAQ TTLSEVCGSG RQKELL-AQG IILQR-YSTV SPEQERLERQ RQ-LPFHMHI NLELLECIYL TSSMFLEVPL 503

**Spo-eIF3c** ADIATQILHN RTMVQIGLCA FRNGMVQETQ YALQDISTTG RVKELL-GQG IQAPK-FGQF TPDQDRLDKQ LV-LPFHMHI NLELLECVYL TCSMLMEIPA 705

**Sce-eIF3c** FDSSLQILFN RVVVQLGLSA FKLCLIEECH QILNDLLSSS HLREIL-GQQ SLHRI-SLNS SNNASADERA RQCLPYHQHI NLDLIDVVFL TCSLLIEIPR 632

**Lmj-EIF3C** SDAPLAILLN RAIAQLGLAA FIAGDIPTAH QLLRTIW-GL RSNQVLIGQS PPPKSVLDDE HA--EMEYRN LL-LPPHMHM PVAQLELASV LSGLLMGVKM 528

**Tbr-EIF3C** SNHN-SVLYN RAVAQLGLAS FIMGDIMQAY ELLSPLWNSW EGPEVLIGQK LPN---LKDE KGDEELRYRD LL-LPPHAHI PYSQLELATM LSTLVVGTVD 520

**Tva-eIF3c** ELPFVRILNN RAFAEIGIAA FMTGDYRTAY NSLKGFTKNP VASNAILGQ- ---------- --------HP RIYAPW-LNI DPAALEAYHY ISAMMLDIPA 582

**Gdu-eIF3c** TD--IDYIFL RLHYCIGIKA FANGRMHQAS THLLLISSLF KGERQT---- ------ISPF LPSVHAFEAE SI-VSW---L DSDLIEAACL LSTLCSKFAL 650

**Hsa-eIF3c** MAAH---ESD ARRRMI---S KQFHHQLRVG ERQPLLGPPE SMREHVVAAS KAMKMGDWKT CHSFIINEKM NGKVWDLFPE ADK-VRTMLV RKIQEESLRT 791

**Cel-eIF3c** MASC---EFE MRRRML---S RSFHYQLKQS EKASLTGPPE NTREHVVAAS KAMLNGDWKK CQDYIVNDKM NQKVWNLFHN AET-VKGMVV RRIQEESLRT 747

**Ath-eIF3c** MAAN---SHD AKRRVI---S KNFRRLLEIS ERQAFTAPPE NVRDHVMAAT RALTKGDFQK AFEVL----N SLEVWRLLKN RDS-ILDMVK DRIKEEALRT 717

**Ani-eIF3c** MAQTSS-SPE MKRRVI---S KTFRRMLDYN ERQVFTGPAE NTRDGVIMSA KFLAAGDWKK AAEML----N SIKIWDLMPQ PDK-VKEMLS QQIQEEGLRT 594

**Spo-eIF3c** MAAASSTASD SRKRVI---S RPFRRMLEYI DRQLFVGPPE NTREYIMQAS KALADGEWRR CEEFI----H AIKIWSLMPD ADK-IKQMLS EKIREEGLRT 797

**Sce-eIF3c** MTAF---YSG IKVKRIPYSP KSIRRSLEHY DKLSFQGPPE TLRDYVLFAA KSMQKGNWRD SVKYL----R EIKSWALLPN MET-VLNSLT ERVQVESLKT 724

**Lmj-EIF3C** EAQN---PYE RNHM-----E RYVYNTVTRT --PDLMGKPF SFKEQVAVAY EHLKAGNYIG AKEQV----E AMTTFDTLPL GKE-TRKRYL QRLKEVALLV 613

**Tbr-EIF3C** EAKK---PYE VTHH-----H RYFYRVINQM QFQPLLGEPI EFREQITAAY TALKLGDYAR SSEVI----K NMKVWDNMPR GTE-ARDTFL QRLKEAALQI 607

**Tva-eIF3c** LTTF---NYD DQVLLI---N GKTHKELQ-- KKVGITACPE SILDKIAVAI ERCKRGEWQL ARETL----- KYEIERYIPD P-----RLFE RDLKLMSLCS 764

**Gdu-eIF3c** LHNR---AHT VANQHLTPLQ RLVLQKILQL VDVGRQVNAT PYEYAIALAF KCLSHNNHDG AISAI----I TCPTWRCLRY YNTHYERCLE EAIKVLSLKF 743

**PCI DOMAIN**

**Hsa-eIF3c** YLFTYSSVYD SISMETLSDM FELD---LPT VHSIISKM-- ---------- ---IINEE-- LMASLDQPTQ TVVMHR-TEP TAQQNLALQL AEKLGSLVE- 873

**Cel-eIF3c** YLLTYSTVYA TVSLKKLADL FELS---KKD VHSIISKM-- ---------- ---IIQEE-- LSATLDEPTD CLIMHR-VEP SRLQMLALNL SDKLQTLAE- 825

**Ath-eIF3c** YLFTYSSSYE SLSLDQLAKM FDVS---EPQ VHSIVSKM-- ---------- ---MINEE-- LHASWDQPTR CIVFHE-VQH SRLQSLAFQL TEKLSILAE- 795

**Ani-eIF3c** YLFTYAPFYD SLSISTLATM FELP---EKK IAAIISRM-- ---------- ---ISHEE-- LAAALDQVND AIVFRKGVEL SRLQSQIVTL ADKSMNLLE- 673

**Spo-eIF3c** YLLAYAAFYD SVSLEFLATT FDLP---VQR VTVIVSRL-- ---------- ---LSKRE-- IHAALDQVHG AIIFER-VEI NKLESLTVSL SEKTAQLNE- 875

**Sce-eIF3c** YFFSFKRFYS SFSVAKLAEL FDLP---ENK VVEVLQSV-- ---------- ---IAELE-- IPAKLNDEKT IFVVEKGDEI TKLEEAMVKL NKEYKIAKER 804

**Lmj-EIF3C** FCYTNRTNFS TMSVVNLAIK FDME---ESD VRRAVNEI-- ---------- ---LSENTT- LSAYWDRDDA YLYLDR-NNA TRLQHLVKGT SESISNLAK- 692

**Tbr-EIF3C** FCYNSRRSFA TISVEIMAKK FDIT---EST VKHVINGI-- ---------- ---ISENNTP LIAVWDRDDQ YLHVDR-SNI SRLQYLVEAT ARSVENIAH- 687

**Tva-eIF3c** YLLTANQYYD AVEIKTLKKQ FDLEYEIDEK TKEKVNSRDV EDCLRMMHQG RSPVVNANIM FQAEFDNDYV KFALNE--KE TILGEYGRTL STKGDML--- 759

**Gdu-eIF3c** WIMNYHAVVS SIYLIDLAKT YGLS---EDK IKTCLRGV-- ---------- ---------- ---------S NIHIKEDIVE FRIQ------ ---------- 793

**PCI DOMAIN**

**Hsa-eIF3c** ----NNERVF DHKQGTYG-- --GYFRDQKD GYR-----KN EGYM--RRGG YRQQQSQTAY ---------- ---------- ---------- ---------- ---------- - 914

**Cel-eIF3c** ----NNEQIL EPRTGRGGYQ GPGSWFPGRN ERQGDKQKGS GGYQGERRGG QGQDGKRGNW GSQGGQQRRH PQKPRAF--- ---------- ---------- ---------- - 898

**Ath-eIF3c** ----SNERAM ESRTGGGGLD LSSRRRDNNQ DYAGAASGGG GYWQDKANYG QGRQGNR--S GYGGGRSSGQ NGQWSGQNRG GGYAGRVGSG NRGMQMDGSS RMVSLNRGVR T 900

**Ani-eIF3c** ----SNEKTL EQRTQGMA-- -NAFQRDQGA GARGGRGPRG G--------G QARGGPRFPG GQQGRRPGGQ QFSGGALGGA IKA------- ---------- ---------- - 741

**Spo-eIF3c** ----ANEKLY EQKTQHTNPQ -ENRRRDKGG SVK------- ------RRNE RTENRNRSDM N--------- ---------- ---------- ---------- ---------- - 918

**Sce-eIF3c** LNPPSNRR-- ---------- ---------- ---------- ---------- ---------- ---------- ---------- ---------- ---------- ---------- - 812

**Lmj-EIF3C** ----HCESRL RANGGR---- --GRGRGGMA GGR-----GG AGVRGAG--G RGRG------ SR-------- ---------- ---------- ---------- ---------- - 731

**Tbr-EIF3C** ----YCEKGG HGNDFR---- -GGRGQGYMR GGRGFGRGGG SDFRGAADYG RGRGRGRARG GQ-------- ---------- ---------- ---------- ---------- - 740

**Tva-eIF3c** ----QNDLIL LEPTSNAY-- ---------- ---------- ---------- ---------- ---------- ---------- ---------- ---------- ---------- - 773

**Gdu-eIF3c** ---------- ---------- ---------- ---------- ---------- ---------- ---------- ---------- ---------- ---------- ---------- - 793

Rezende et al. – Figure S4

**Hsa-eIF3d** MA-------- -KFMTPVIQ- DNPSGWGPC- [ 7 AAs ] D-MPYQPFSK GDRLGKVADW TGA----TYQ [11 AAs ] -----FGGGS QYAYF----- 61

**Cel-eIF3d** MAL------- PKFELLSLA- DNTVGWGPL- [ 7 AAs ] EPVPFQQFNK ADRIGRVADW IGVDRFYRRG [12 AAs ] -----ANAGS QFDYI----- 68

**Ath-eIF3d** MVT------- EAFEFVAVP- FNSDGWGPPD [20 AAs ] N-VPFASFSR SDKLGRVADW TRN----LSN [10 AAs ] -----SDPSA VFDFSAFAID 82

**Ani-eIF3d** MAP------- ISIADIVAAL PAEDTWGPAT [ 7 AAs ] G-VPYAPFSK GDKLGRMADW TAE----SKD [16 AAs ] -QVYGAGSSS LFAVQ----- 69

**Spo-eIF3d** MA-------- TGFKLPELA- PVKSAWGPP- [ 7 AAs ] D-IPYAPFSK GDRLGKIADW SVD----QPK [19 AAs ] YQTYGYGASS IFGYQ----- 67

**Lmj-EIF3D** M--------- -SFELPELYI NPQFSWGPP- [ 7 AAs ] DGAPFELYSK ADALEAV-DW FTYKREADVS [ 8 AAs ] -----VAANK RKAFK----- 65

**Tbr-EIF3D** MFL[27 AAs]MAFVLPQIHI NEQSSWGPP- [ 7 AAs ] ETTVGSLYHK REAVEPF-DW LRVL------ [ 2 AAs ] -----VEGGR QREFT----- 89

**Tva-eIF3d** M--------- ---------- -QQTASGDP- [ 2 AAs ] DVNEFITFFK G--------- ---------- ---------- -----SSHNY GYIYT----- 32

**Hsa-eIF3d** ---------- HEEDESSFQL VDTARTQKTA YQRN---RMR FAQRNLRRDK DRRNMLQFNL QILPKSAKQK ERERI----- -RLQKKFQKQ FGVRQKWD-- 151

**Cel-eIF3d** ---------- HGMDEHNFQL VDTSKPMARN PQRNFRVRQM HLRKMMQKEN EKREMVNQST NLRMKRSIAK EQQRAFKMWQ RRGGNARQGQ RGQGGRFG-- 168

**Ath-eIF3d** EGFGLASSGG NPDEDAAFRL VDGKPPPRPK FGPKWRFNP- ------HHNR NQLPQRRDEE VEAKKRDAEK ERARRDRLYN NNRNNIHHQ- ---------- 174

**Ani-eIF3d** ---------- VAEDESSFSV VDNTRSSAKR TFGRGGGTV- ------FRGR AQRGGAQRGG RAGFQRVGAG RGQGGDRYYD NRSGG--RGN RGRRFGWKDY 166

**Spo-eIF3d** ---------- HSEDESSFSV IDRGSVNRTR TSARNGGTL- ------LKVR GRGQNVQRGG RGGRYGSSGG RG-AGDTVVS RSSGA--GGA RGRRFGWKDY 166

**Lmj-EIF3D** ---------- QVEDNARLNA LRTEREKTVM FNPRNNG--- ---------- ---------- ---PRNAKLA AGKKGGK--D NRNNNQRRN- ---------- 124

**Tbr-EIF3D** ---------- IVEDEKRNKI LKSTRVKERR QVPDWSA--- ---------- ---------- ---PRRHQ-- ---------N SRRNFQNKP- ---------- 133

**Tva-eIF3d** ---------- TEISEDLYRQ VTYHSIYSF- TAPQLTFRL- ------RQTF DDRNYVEPKG PGAKRRTIA- ---------- ---------- ---------- 83

**Hsa-eIF3d** QKSQKPRDSS VEVRSDWEVK EEMDFPQLMK M-RYLEVSEP QDI--ECCGA LEYYDKAFDR ITTRS-EKPL --RSIK-RIF HTVTTTDDPV IRKLAK-TQ- 242

**Cel-eIF3d** GDRPKERLPS VQVRPEWVVL EEMNLSAFSK L-ALPNIPGG DDIGDHQYGS LQYYDKTIDR VSVKN-SIPL --QRCA-GVF YNVTTTEDPV IQELAQGGA- 262

**Ath-eIF3d** RREAAAFKSS VDIQPEWNML EQIPFSTFSK L-SY-TVQEP EDL--LLCGG LEYYNRLFDR ITPKN-ERRL --ERFKNRNF FKVTTSDDPV IRRLAKEDK- 266

**Ani-eIF3d** DKPQRTREPS VNVRPDWNML EEVDFSRLSK L-NL-EAPEG EDV--DSYGF LYHYDRSYDK APVKNAERRL --QSLD-RAA YNVTTTQDPV IQELAEKNE- 258

**Spo-eIF3d** DKHQRLRNAS VTVGDDWQLL DEVEFSHLSK L-NL-AAAAP VTV--DSYGY IYPYDKSFDK IHVKS-EKPL --QALD-RVH YNPTTTEDPV IQKLALNSD- 257

**Lmj-EIF3D** RRKVTELPNT YNASAMAVVQ HVMKQTDMTK L-RMSALPKV IEL--GLYGT PPIYNTGIEA ATCAR-PLPL DESKYDEDYF MRGLTTEDPE LRKIMGETQ- 219

**Tbr-EIF3D** KRNITLPPDT VKVPSDAVIL EQFRQAELAK MPNLTSLPTV SDI--SQHNR PPVYKNEMDK ASCKA-PIPL NEKETK-VDF TRSDSFTDNV LRGILKSEPP 229

**Tva-eIF3d** TASPYDFHGL VEPNVDWQQI HTIEGAALND A-TFNGKITE EDI--KFTGR AKVFNNSLLS TRPHK-KRAL PEANYK---- EESPILEDPV FKEIAQEKTK 175

**Hsa-eIF3d** ---GNVFATD AILATLMSCT RSVYSWDIVV QRVGSKLFFD KRDNSDF--- -DLLTVSETA N---EPP--- ---QDEGNSF NSPRNLAMEA TYINHNFSQQ 326

**Cel-eIF3d** ---GNVFGTD IILATLMTAP RSVYSWDIVA YRVGDKLFFD KRNTRDILNP VETLTVSETS A---EPP--- ---SFDGNGI NNAKDLATEA FYINQNFRRQ 350

**Ath-eIF3d** ---ATVFATD AILAALMCAP RSVYSWDIVI QRVGNKLFFD KRDGSQL--- -DLLSVHETS Q---EPL--- ---PESKDDI NSAHSLGVEA AYINQNFSQQ 350

**Ani-eIF3d** ---ATIFATS DILSMLMCAP RSVYSWDIVI VHQGNKIYFD KREGASL--- -DLVTVNENA A---DAPLEL ADSANKQDAI NTPSALAMEA TFINHNFALQ 348

**Spo-eIF3d** ---ANIFITD SILSLLMCST RSVYPWDIVI THQSGKLFFD KREGGPF--- -DYLTVNENA Y---DSPMD- ---ADNREGV NSPGALSVEA TYINQNFCVQ 343

**Lmj-EIF3D** -RYPLVVVTD EILSLLMVCT RSSYPWHIRV LNYNNIWILV KGEDSNI--- -EKQWVSETA GHEVRPS--- ---EGADNRA ERISSLGEES TKVYDCFARA 308

**Tbr-EIF3D** GTYPIVVATD EVLALLMTCS RSVYSWHLHF YRVGRFYFIS KVDGCNV--- -EKQWVDETA DVSRVPS--- ---ETEVVET DRTSSLEAES SKVNNFFVAQ 319

**Tva-eIF3d** AN-IRVFASA KALAAISVVN RSVCPFELIF KKDGINLWVY QRTE------ -ESPAIFESS FETITIPVNQ ---RRDDTRE EFLRNL-VEA SNIAAQFQAA 263

**Hsa-eIF3d** CLR-----MG KERYNFPNPN PFVEDDMDKN EIASVAYRYR RWKLGDD--- --IDLIVRCE HDGVMTGANG -EVSFINI-K TLNEWDSRHC ---NGVDWRQ 411

**Cel-eIF3d** VVK-----RN DAGFTFKNAR APFEDEETGE --SGTAYKYR KWNLGNGVDG KPVELVCRTE LDGVIHGLGN -ETQTLTI-K AFNEWDSTQS ---GGVDWRT 438

**Ath-eIF3d** VLV-----RD GKKETFDEAN PFAN---EGE EIASVAYRYR RWKLDDN--- --MHLVARCE LQSV-ADLNN -QRSFLTL-N ALNEFDPKYS ----GVDWRQ 430

**Ani-eIF3d** TVV----ESE DSKVSLAHPN PFYNAAEETE PLASKAYKYR RFDLSLERDD EPVNMIVRTE VDAIMKNPVN GEDQQLLV-K ALNEFDSKAQ GSGGALDWRS 443

**Spo-eIF3d** ALR----ETE EEKYKLPHPN PFYNSKEESE PLAAHGYIYR DVDLSLETDE KPVKLMVRTE VDGYVKNPAN -DVQYISI-K ALNEYDPKFT NVTGSVDWRS 437

**Lmj-EIF3D** SCAKSFAQVR TNRSPFSRKQ P--------- ----RMYSYR RYIMHDGTPD R-YDIVVRCE VDALMPRTND ----RVRC-F ALLEQCVVSE ---KDSSWRR 386

**Tbr-EIF3D** SCTAARYQMD CEKSPFPGKH P--------- ----RLYRYR RFVMHADTKD R-YDLIVRCE VDAMQGD--- ---KHIRL-F GLLEHCIKGE ----ENDWRK 394

**Tva-eIF3d** ANS-----GD GPTFTLGDDL EHPGDDDD-- ---SKPYLYR RLVLDN---- --VEFIIRAE IDCLRDPLQP GERPSIAICR TFNNLPTSLR ----QVSYAT 343

**Hsa-eIF3d** K-LDSQRGAV IATELKNNSY KLARWTCCAL LAGSEYLKLG YVS---RYH- ---VKDSSRH VILGTQQFKP NEFASQINLS VENAWGILRC VIDICMKLEE 503

**Cel-eIF3d** K-LDVQKGAV MATEIKNNSA KVAKWTLQAL LAGSDTMKLG YVS---RNN- ---ARSTQNH SILLTQYVKP TEFASNIALN MDNCWGILRC VIDSCMKQKP 530

**Ath-eIF3d** K-LETQRGAV LATELKNNGN KLAKWTAQAL LANADMMKIG FVS---RVH- ---PRDHFNH VILSVLGYKP KDFAGQINLN TSNMWGIVKS IVDLCMKLSE 522

**Ani-eIF3d** K-LWSQRGAV VATEMKNNSV KLARWTTQAI LAKADGMKLG FVS---RAN- ---PRSAAGH VVLGVVGYKP RDLAAQMNLN LGNGWGIVRT IVDRIRALDA 535

**Spo-eIF3d** K-LESQRGAV FATEMKNNSC KLARWTVEAL LAGVDSMKVG FVS---RSN- ---ARDAQHH GILGVVAYKP ADLASQMNLS LSNGWGIVRT IADVCLKMPD 529

**Lmj-EIF3D** EGLLKNAASF LPIEYAHNGC KIARWTALSL LSDAKLMKIG FVTCEEKMEK GQRVFDHKQH EVLSVQTYSP ASLATQFGID VSNMWAIVDH IMRPFIEGHS 486

**Tbr-EIF3D** M-LASQTATC ISEEYRRNAQ KMARWIALCH LSGAH-MKIG FIS---RCRK GAGVFDPLRH EVLATFTNDP SPLAAQLGIK VANMWTVADT IITAFVQSDF 489

**Tva-eIF3d** D-LEKARSSL LLAEVKDNSN IFAKWVAIGR FMDCQQIFLG FVE---RKV- ---STNKQQH VLLASERQAY GRGASIITLP EASMMGQIYQ VFQKMIASDD 435

**Hsa-eIF3d** -------GKY LILKDPNKQV IRVYSLPDGT FSSDEDEEEE EEEEEEEEEE ET-------- ---------- ------ 548

**Cel-eIF3d** -------GKY LLMKDPQSPV IRLYSLPEGT FESERESSDE ENSDDDQ--- ---------- ---------- ------ 570

**Ath-eIF3d** -------GKY VLVKDPSKPQ VRIYEVPPDA FENDYVEEPL PEDEQVQPTE ENTEGAEASV AATKETEEKK ADDAQA 591

**Ani-eIF3d** DEDEDKVTKY VLIKDPNRPV LRLYSVPATT FEEEEEVAAE EQEAAEEEAE E--------- ---------- ------ 586

**Spo-eIF3d** -------GKY VLVKDPNRPI LRLYSVPPNT FEEAAGPSLE ASSTA----- ---------- ---------- ------ 567

**Lmj-EIF3D** ------ICPS ILMKPGDKSE LIVVEEEDDD DESDEDDSEE DGDDGDDKDD E--------- ---------- ------ 531

**Tbr-EIF3D** ------SEAA LVKRSGDTSI LLVEKCEEEF YEEEEDEEDD DEEDDDDDGE EDG------- ---------- ------ 536

**Tva-eIF3d** -------GIY HFQRDSTHRV VKIFKEVKDK VKLDEE---- ---------- ---------- ---------- ------ 464

Rezende et al. – Figure S5

**Hsa-eIF3e** MAE-------- YDLTTRIAHF LDRHLVFPLL EFLSVKEIYN EKELLQGKLD LLSDTNMV-D FAMDVYKNLY ----SDDIPH ALREKRTTVV AQLKQLQAET 88

**Cel-eIF3e** MST-------- FDLTQRMAPF LDLHLIIPLL EFIEPRGIYD EKSLTEMHRQ LLTKTNMI-D SVIETYN--- ----GKPIPA AIEAKKKQII KERDELKSKV 85

**Ath-eIF3e** MEE[4 AAs ] YDLTPLIAPN LDRHLVFPIF EFLQERQLYP DEQILKSKIQ LLNQTNMV-D YAMDIHKSLY H---TEDAPQ EMVERRTEVV ARLKSLEEAA 93

**Ani-eIF3e** MAA[27 AAs] YDLLPKLIPY LDRHLVFPLL EFSSGSQ-ED DKEMIRAKYE LLKHTNMT-D YVANLWKEIN D---SDTIPD EFVKKREEVL AKLQHYQEQS 115

**Spo-eIF3e** MGS[11 AAs] YDLSQKIMQH LDRHLIFPLL EFLSLRQTHD PKELLQAKYD LLKDTNMT-D YVANLWTNLH GGHTDEDMAN AFAEKRRSVL QELSELEEEV 103

**Lmj-EIF3E** M---------- -DMLTKLLPY MDKHLALGLL NHYAQSG--- -EDVQDAMMK LIETTGLNAD GSIKAE---- ----TEEMMA KATAAAQPAL KEFFDESEDD 78

**Tbr-EIF3E** M---------- TEMLSCLVPY LDKHLVLGLL YFYDDQG--- -VDVGDALRF VQATTALTPE GEVSLE---- ----QENKIR ETAERARPAL DMFFEQNVSD 79

**NUCLEAR EXPORT SIGNAL**

**Hsa-eIF3e** EPIVKMFEDP ETTRQMQSTR DGRML----- FDYLADKHGF RQEYLDTLYR YAKFQYECGN YSGAAEYLYF FRVLVPATDR NALSSLWGKL ASEILMQNWD 183

**Cel-eIF3e** DSVVAILEIP EVKEMMDN-- NRERDGNVRI LEHLTQNHNF TVDMVDTLFK YSKFMYECGN YTVASVCLYY YRNLVNQADP NYLNALYGKL ASEILLQEWE 183

**Ath-eIF3e** APLVSFLLNP NAVQELRA-- DKQYN----- LQMLKERYQI GPDQIEALYQ YAKFQFECGN YSGAADYLYQ YRTLCSNLER -SLSALWGKL ASEILMQNWD 185

**Ani-eIF3e** AKITELLQDE DVVGNLRS-- DKVAN----- LKFLEEEHGV TADMVNSLFD YGRFQYSCGS YGNAAELLYQ FRVLSTDNDK -VASATWGKL ASEILTTSWD 207

**Spo-eIF3e** QGILGVLENP DLIAALRQ-- DKGQN----- LQHLQEHYNI TPERIAVLYK FAQFQYNCGN YGGASDLLYH FRAFSKDPEL -NASATWGKF ASEILTVDWD 195

**Lmj-EIF3E** HSTYQFKLTE TEIGERRT-- QGELS----- HEFLSEKKGI TAAVMNALYK LAYLYYDTGA YGDASELLTL CQCVSGYDNI RSDTILWGKL MSDIGAVNWQ 171

**Tbr-EIF3E** NCTYQLCLTE SRIDELRG-- KGELS----- RGFL-EKEGI TPKVMTAVMD LAFLYYDAAR YGDASELLSL LQCVTGYE-L GESKLLWGRL VCDTCSCRWP 170

**Hsa-eIF3e** AAM---EDLT R------LKE TIDNNSVSSP -LQSLQQRTW LIHWSLFVFF NHPKGRDN-- ---IIDLFLY QPQYLNAIQT MCPHILRYLT TAVITNKDVR 268

**Cel-eIF3e** HAR---DDLL K------LRA YIDANPFDTE -WELVTQRAW LMHWALFVYY NYPKGRDE-- ---IIEMFLN QQPYLNAIQV LAPHLLRYLA VAVVTSKSRQ 268

**Ath-eIF3e** IAL---EELN R------LKE IIDSKSFSSP -LNQVQNRIW LMHWGLYIFF NHDNGRTQ-- ---IIDLF-N QDKYLNAIQT SAPHLLRYLA TAFIVNKRR- 268

**Ani-eIF3e** GAM---EEVQ K------VKD SIETRLFNNP -LGQLQNRSW LIHWSLFPFF NYDPARDV-- ---LTDLF-F SPAYINTIQT SCPWILRYLA AAVITNRNRA 291

**Spo-eIF3e** GAM---EELG K------LRE MVDSKSFKDS -AVQLRNRTW LLHWSLFPLF NHANGCDT-- ---LCDLF-F YTPYLNTIQT SCPWLLRYLT VAVVTNQNNA 279

**Lmj-EIF3E** SAMRIAEEIR R------VHN ASEEDLFGAP NTTTVRARVW LLHWVLFPFF KGGLQYSLQL LYFIFDHR-H DQTYRKAVET VCPHYLRYIC AAVLLHRTRY 264

**Tbr-EIF3E** SAIAAAEKLW KQQGADGSEN KSGKTTLRGD NGTSVTERVW LLHWALFPFF KGGNQYSTHL LNIVFDNK-T DSIYQCVVET VCPHYLRYIC AAAILNTHRR 269

**Hsa-eIF3e** KR-------- -RQVLKDLVK VIQQESYTYK DPITEFVECL YVNFDFDGAQ KKLRECESVL VNDFFL---- -------VAC LEDFIENARL FIFETFCRIH 348

**Cel-eIF3e** ---------- -KNSLKDLVK VIDIERHSYK DPVTDFLTCL YIKYDFDEAQ EMLQKCEEVL SNDFFL---- -------TAV LGDFRESARL LIFEMFCRIH 346

**Ath-eIF3e** ---------- -RPQLKEFIK VIQQEHYSYK DPIIEFLACV FVNYDFDGAQ KKMKECEEVI VNDPFLGKRV EDGNFSTVPL RDEFLENARL FVFETYCKIH 357

**Ani-eIF3e** HKN----SNV YQKQLKDLIR VVRQEGYEYS DPITDFVKAL YVDFDFEEAQ KKLGEAEDVL RSDFFL---- -------VSA ADAFVEAARH LISESYCKIH 376

**Spo-eIF3e** NQKPRNPRQS YQRRMRDLVR IISQENYEYS DPVTSFISAL YTEVDFEKAQ HCLRECEEVL KTDFFL---- -------VSL CDHFLEGARK LLAEAYCRIH 368

**Lmj-EIF3E** SN-------- -FVSAAELVE SI----YEYS DPLTQLVS-L IQKASFEDAI ALLPEVRRMI KEDYFL---- -------ADY EDELIENAKR MIFSKYMSLH 339

**Tbr-EIF3E** SA-------- -LRRAAEMVG RI----YEYS DPLTQLVREI TNYRSFEDTL ELLPKVSELA QGDYFL---- -------NLH ADNLVENAKR LIFTQYVVTH 345

**PCI DOMAIN**

**Hsa-eIF3e** QCISINMLAD KLNMTPEEAE RWIVN--------L IRNA-R -------LD-AKIDSK LGHVVMGNNA VSPYQQVIEK TKSLSFRSQM LAMNIEKKLN [18 AAs ] 445

**Cel-eIF3e** QCITIEMLAR RLNMSQEEAE RWIVD--------L IRTY-R -------IEGAKIDSK LGQVVMGVKS VSIHEQVMEN TKRLTLRAQQ IALQLEKGRQ [ 6 AAs ] 432

**Ath-eIF3e** QRIDMGVLAE KLNLNYEEAE RWIVN--------L IRTS-K -------LD-AKIDSE SGTVIMEPTQ PNVHEQLINH TKGLSGRTYK LVNQLLEHTQ [ 5 AAs ] 441

**Ani-eIF3e** QRIDIKDLST RLGLNQDEGE KWIVN--------L IRDT-R -------VD-AKIDYK EGTVIMNHPP QSVYQQVIEK TKGAFFRTQV LSAAVAK--- --------- 452

**Spo-eIF3e** SVISVDVLAN KLEMDSAQLI QLVEN[34 AAs]L ITEA-E[14 AAs]FK-AKLDGE --SIIIEHPT YSAFQQIIDR TKSLSFESQN LEQSLAKSIS [ 7 AAs ] 501

**Lmj-EIF3E** SVVSIPYVAE QLDMSKADAE VWLVN--------L ISESVK -------HR-AKIDSV NEQLNVEPQT RSLESLIYDK LDTVMR---- ---------- --------- 405

**Tbr-EIF3E** SVVSIPYMAE RLEMSAAGAE VWLAD--------L ISET-K -------QR-AKIDAV TGQMFVGSQV RSVHQTVLDR LEPVDHGRR- ---------- --------- 413

**PCI DOMAIN**

Rezende et al. – Figure S6

**eIF3f alignment**

**Hsa-eIF3f** M [99 AAs] VVRLHPVILA SIVDSYERR- -----NEGAA RVIGTLLGTV -DKH-SVEVT NCFSVPHNES EDEVAVDMEF AKNM---YEL HKKVSPNELI 169

**Cel-eIF3f** M [ 4 AAs] TVNVHPGVYM NVVDTHMRRT KSSAKNTGQE KCMGTLMGYY -EKG-SIQVT NCFAIPFNES NDDLEIDDQF NQQM---ISA LKKTSPNEQP 90

**Ath-eIF3f** M [23 AAs] TARIHPLVIF NVCDCFVRR- -----PDSAE RVIGTLLGSI LPDG-TVDIR NSYAVPHNES SDQVAVDIDY HHNM---LAS HLKVNSKETI 106

**Ani-eIF3f** M [27 AAs] NVVIQPQALF SILDHSLRR- -----NADQE RVIGTLLGTR SEDGTEVEIR STFAVGHTET TDQVEVDMEY QKQM---LAL HLKANPKEVL 109

**Spo-eIF3f** M [19 AAs] NIVIEPAVLF SILDHSTRK- -----SENNQ RVIGTLLGTR SEDGREIEIK SCFAVPHNES SEQVEVEMEY HRAM---YHL HLKANPREVV 102

**Lmj-EIF3F** M [ 4 AAs] AIQLTPYPAA FIIEHAKRK- ---------K YAAGYILGTV -RAE-EIVIT D--FIPHTHK DTEVPNTKAH RAELTRRRAA KRRYTT-QDL 80

**Tbr-EIF3F** M -------- ---------- ---------- ---------R NSAGYLFGSY -SDG-QITVT D--YIPCTHE FEGDKFPQWC LDEFKARVDV KKHYNSGVAV 58

**MPN DOMAIN**

**Hsa-eIF3f** LGWYATGHDI TE-HSVLIHE YYS------- --REAP---- -------NPI HLTVDTSLQ- --NGRMSIKA YVSTL----- ---------- ----MGVP-- 224

**Cel-eIF3f** VGWFLTTSDI TS-SCLIYHD YYVRVITEAS ARRESP---- -------PIV VLTIDTTFSG DMSKRMPVRA YLRSK----- ---------- ----AGIP-G 158

**Ath-eIF3f** VGWYSTGAGV NG-GSSLIHD FYA------- --REVP---- -------NPI HLTVDTGFT- --NGEGTIKA FVSSN----- ---------- ----LSLG-- 161

**Ani-eIF3f** VGWYATSSEL NT-FSALIQN FYS------- --GQGD---- --GTWPHPAV HLTVSTEA-- --GKDIETRA YISAP----- ---------- ----VGVT-A 169

**Spo-eIF3f** VGWYATSPDL DA-FSALIQN LYA------- --SPAEPGTA PLGTYPHPCV HLTVNTDV-- --SSPLAIKT YVSSP----- ---------- ----VGIT-- 167

**Lmj-EIF3F** IGWYSAGQPG ADLTEEDYQL WC-------- ---NAPSVIF Q----GRHCL HLHCEMPHED GTTPKVTWTA SVVFEDPADR [ 8 AAs ] ----VTLAPM 159

**Tbr-EIF3F** IGWYVAGAPE PG-GEAVFKR WC-------- ---EAPGVIF ARGGLKSQAL MLLARMPCDG DMTLK--WEA YITNNSIQEE [ 8 AAs ] QKLNVTIA-A 141

**MPN DOMAIN**

**Hsa-eIF3f** -GRTMGVMFT PLTVKYAYY- -----DTERI GVDLIMKTC- --FSPNRVIG LSSDLQQ--- ---VGGASAR IQDALSTVLQ YAEDV-LSGK VSADNTVGRF 307

**Cel-eIF3f** AAGPHCAIFN PLRVELAAF- -----PGELV AMQLIEKAL- --DSRRREAT LESGLEQ--- ---LETSTAQ MIEWLERMLH YVEDVNKNGE KPGDAQIGRQ 243

**Ath-eIF3f** -DRQLVAHFQ EIPVDLRMV- -----DAERV GFDVLKAT-- ------SVDK LPNDLEG--- ---MELTMER LLTLINDVYK YVDSV-VGGQ IAPDNNIGRF 239

**Ani-eIF3f** ERAADSAAFI PVPYEIRYG- -----EAEKS GLEAIGAAR- --DAEERRAN IFTDIEA--- ---LERAIED VLGMIDRVSR YVESV-IDEE APASTALGQY 253

**Spo-eIF3f** ERLADSCAFV PTPFTIRDD- -----EAVRS GLKAVAAPK- --NDPSRLAS LFTDLQQ--- ---LRRSTLE LLSMIERVSD YVQNV-IDGS SPANVAVGRY 251

**Lmj-EIF3F** NNLASDVMLS HITSLVLYNG GRPFPRSKLQ NLDEVAYVA- --SIDHKSST DAVDEEQRR- ---------- ----LERAVA AAQEIVTGGS GGGKEEHE-- 239

**Tbr-EIF3F** ESPSMNVLLA EIASKALYGG GTPHATSRIT SLDLVARGAE MMELEGNSGN NKRDNNRRDG DRPVEEALMR VQNGMMQDIS NAESALAGGN NNKEGNLD-- 239

**Hsa-eIF3f** LMSLVNQVP- KIVPDDFE-- -------TML NSNINDLLMV TYLANLTQSQ IALNEKLVNL --------- 357

**Cel-eIF3f** LMDIVTASSN NMQPEKLD-- -------TLV KNTLRDYVMV SYLAKLTQTQ LQVHERLVSA --------- 294

**Ath-eIF3f** IADAVASLP- KLPPQVFD-- -------NLV NDSLQDQLLL LYLSSITRTQ LSLAEKLNTA A[ 3 AAs] 293

**Ani-eIF3f** LLNTLALAP- KVEPADIE-- -------RDF NNHIQDVLVV SYLANTIRTQ MELSNRLATA Q[42 AAs] 346

**Spo-eIF3f** LMKCFSLIP- CVEGQDFE-- -------KIF SSHLQDVLVV VYLANTLRTQ VDIASRLNLL P-------- 302

**Lmj-EIF3F** MMTAVENFR- AIRDEALKRQ RDQTGRMDFN SQQFKDALMI KCAATILRRE IDQIEHLSTV Y[27 AAs] 326

**Tbr-EIF3F** SAAIVESYK- RILEEKGK-- --QGERGDFI TESYTDALAI KYQMFVLRRV LNDIERNNNP E[24 AAs] 318

**eIF3h alignment**

**Hsa-eIF3h** MASRKEGTGS TATSSSSTA- -GAAGKGKGK GGSGDSAVKQ VQIDGLVVLK IIKHYQEEG- -----QGTEV VQGVLLGLVV --EDRLEITN CFPF------ 84

**Cel-eIF3h** M--------- ---------- -------STA VTITAPSVKH ILLDSLVVMK IVKHVDSELH AGISEVSGDA CAGVLTGLVF LEDSRLEITN CFPT--VRNE 72

**Ath-eIF3h** M--------- -----ATMA- -RSFLQAISK DEAVAPPLRV VQIEGLAVLK IIKHCKE--- -----FSPTL VTGQLLGLDV --GSVLEVTN CFPF------ 68

**Ani-eIF3h** M--------- ---------- ---------- -AEKEVPLTA VKVEALVVMK IIKHGSQ--- -----AFPTT ATGSIVGMDV --DGTLEITN SFPF------ 54

**Spo-eIF3h** M--------- ---------- --SDTTSLNV PELESPPIER VELESLLVMN IIKHCRDS-- -----FPNMG TIGQLVGIDI --DGVLQVSS SFES------ 64

**Lmj-EIF3H** MYAGFPRLNV TEAQETVDLD FELTNEKGCV KVTEFPVPKV VNLSFPAVCA LLYNARTA-- -----TSSRN IFGSVIGLQL --QDTVEVTD VKTN------ 85

**Tbr-EIF3H** MFHGFPQVQV VSDDAPTG-- -ESIFAKDSD SHVELPPAKK VRISFPALTH LGSFSQDA-- -----TCHSA VGGRLCGVQQ --GDTILITS VLPS----LG 84

**Tva-eIF3h** M--------- ---------- ---------- ---ENKTPDS ITISPRAIFE IIKDIQSG-- -----RSGSD AQGYLYGLP- --ENPIEVTS AFPAYSLKAE 58

**MPN DOMAIN**

**Hsa-eIF3h** PQHTEDDADF DE-------- --------VQ YQMEMM---- ----RSLRHV NIDHLHVGWY --QSTYYGSF VTRA-LLDSQ FSYQH-AIEE SVVLIYDPIK 156

**Cel-eIF3h** PVMDDDANAA QQ-------- ---YEEQKQH EMLDML---- ----RKFRTM NIDYEIVGFY --QSHQFGAG FSHD-LVESM FDYQA-MGPE NVVLIYDPIK 149

**Ath-eIF3h** PVRDDDEEIE AD-------- -------GAN YQLEMM---- ----RCLREV NVDNNTVGWY --QSTVLGSY QTVE-LIETF MNYQE-NIKR CVCIIYDPSK 141

**Ani-eIF3h** PVVEVPAESH FDNTAPNPAA AAPRAKANAA YEAEMV---- ----RMMREV NVDANNVGWY --TSANMGNF INMN-VIENQ FFYQKEMNER TVALVHDVSR 143

**Spo-eIF3h** PSVLENEESA VNKSV----- ---SGKARQA HTEAML---- ----NRLQYI GAVTGHVGWY --LGAYVSSF LSSPFFVETQ YAYQK-ANPN SIAFLYDLSQ 145

**Lmj-EIF3H** PSIDIEEDDR MT-------- -EEERMERVR KERKLLDADK AIFENMYNQE NLDTNVIGQF VVSSARFN-P FSTR-TMRKL QELHR-ESLP AILLTYDPFR 173

**Tbr-EIF3H** ASGDAEDERD DT-------- -AESKQQQKM YEK-VK---- ----EMLRKE CLDSYSVGYF IVSSACVNDP YSVV-TADRL ANLAI-DGHP SVLLVYDPFR 164

**Tva-eIF3h** PSETADDIVK EE-------- ---YRNSIKN FQKTHL---- ----EELKAL NCDYEHVGRY --TSRNVGGR IHFR-DLNRQ WEEQVETSSD LFTLVVTING 136

**MPN DOMAIN**

**Hsa-eIF3h** TA-QGSLSLK AYRLTPKLME VCKEKDFS-- ---------- PEALKKANIT FEYMFEEVPI VIKNSHLINV LMWELE---- ---------- ---------K 220

**Cel-eIF3h** TR-QGQLSLR AWRLSTAALD LASKNDWR-- ---------- PELVKAAGLT YQNMFEELPI IIKSSYLNNV LMSELSL--- ---------- ---------A 214

**Ath-eIF3h** AD-LGVLALK ALKLSDSFME LYRGGNFT-- ---------- GEKLREKNFS WMDIFEEIPI KVSNSALVSA FMTELE---- ---------- ---------T 205

**Ani-eIF3h** SA-QGSLSLR AFRLSPKFMA AFKENKFT-- ---------- SEELQKSNLR YQDILVELPV EIHNSHLITS FIHQLQTPTQ ATPSDLPPSL AALESSQYAK 230

**Spo-eIF3h** SS-NGTLYMR AYQLTPEFMA AHEEKTWT-- ---------- ASSLNSHNLT PSNVIRELPI VIHNSHLATC LLHSLSE--P PTPASTLTAE AALEDCE--S 228

**Lmj-EIF3H** TGLLGRPHIR AYTPTAAYYK YDSLVSTERG YQRRRSL--- AQYAKESGIT KEGVLHEIPV KLEVDAFHK- -LNLMNV--- ---------- ---------P 246

**Tbr-EIF3H** TGLMGKLYLR AFVPTDAFLD FSRKNKKTKK TDKKSGMQEE ARLLRACSVP KQGVLREVKV EVEVDAYQLF CLNGIDI--- ---------- ---------A 242

**Tva-eIF3h** SS----LSTR AFRVSEEACE FMKEHDIN-- ---------- EIGYIDPVFF YDRFVQELQV TFALTKLDEA LIQEMLS--- ---------- ---------R 198

**Hsa-eIF3h** KSAVAD-KHE L--LSLASSN HLGKNLQLLM DRVDEMSQDI VKYNTYMRNT SKQQQQKHQY QQRRQQENMQ RQSRGEPPLP EEDLSK-LFK PPQPPAR--- 313

**Cel-eIF3h** KSCSSD-KYS TRHFDLGSKK SLEKSVRAMM ANVDELNKSI QSLTKYTIDK QRHDNMVFSL TQKRQQENES RVARGDPTIP MDDIKR--IK APQLQTRNGL 311

**Ath-eIF3h** DTPVSQGDYD R--LHSSTTP FLENNMEFLI KCMDDLSMEQ QKFQYYYRNL SRQQAQQQAW LQKRRTENMA RKSAGEEPLP EEDPSNPIFK AIPEPSR--- 300

**Ani-eIF3h** SSVLAP-NFD N--LSLSIDP FLEKNCDLLL DSIEVHHTET NNFQYYQRSL AREQAKITAW QNKRKTENAS RAALKQPLLP EDEWQR-LFK LPQEPSR--- 323

**Spo-eIF3h** NLPLTE-TFS N--FEVSLGT RYRKNIELLL ESTDEFHYEQ GNLGFHQRQL AREQAKIQQW IAKRKAENAN RAAENLQPLP LDDWKR-IFK LPAEPRL--- 321

**Lmj-EIF3H** PTAVGDS--- ---FKAIQSM AVSNYIEALL SSIR------ NNTDQLKQKL DYEG------ -NVKDNGNV- ---------- ---------- ---PVGQD-- 301

**Tbr-EIF3H** PLPSNST--- ---VHHSDSM A--DYKVALL ESVQ------ RNLSDLEGKL SRETSR---- -NQRDENNV- ---------- ---------- ---PPARG-- 297

**Tva-eIF3h** FNLISDV--- ---FILRDMN AMQDQMIQIY DSLDKIKDEV TKATADKFKI EENRAARKQW VDERKEKNLS RIAHKLEPLP LEDVDKEIPK LDQSRKR--- 289

**Hsa-eIF3h** MDSLLIAGQI NTYCQNIKEF TAQNLGKLFM AQAL------ ---------Q EYNN 352

**Cel-eIF3h** LDELLASFDT NALADFSKTV TSENITKMFI AEAVAEEKVA GTKDRTLSSV SSTR 365

**Ath-eIF3h** LESFLITNQV SNFCGQINGV AGQNFSRLYL TKAL------ ---------- -HDN 337

**Ani-eIF3h** LDSMLNSRQV EQYARQVDSF VSATTGKMFA VKGN------ ------LLPG ETAK 365

**Spo-eIF3h** LDSLLISSQI MK-STQIDEQ SSAFLSKLAG VRNA------ ---------- -YAS 357

**Lmj-EIF3H** AETMLLMKHV RDQTDHLDAL CDSALLYSSL LRDL------ ---------- ---- 335

**Tbr-EIF3H** VDTMLALVQL REQAQHLEAL CDGTLLISSL LRDL------ ---------- ---- 331

**Tva-eIF3h** ---------- ----DAIDNI YDFKARADAI LAELKDEEKK IDTLSSLSAQ TEKK 329

Rezende et al.– Figure S7


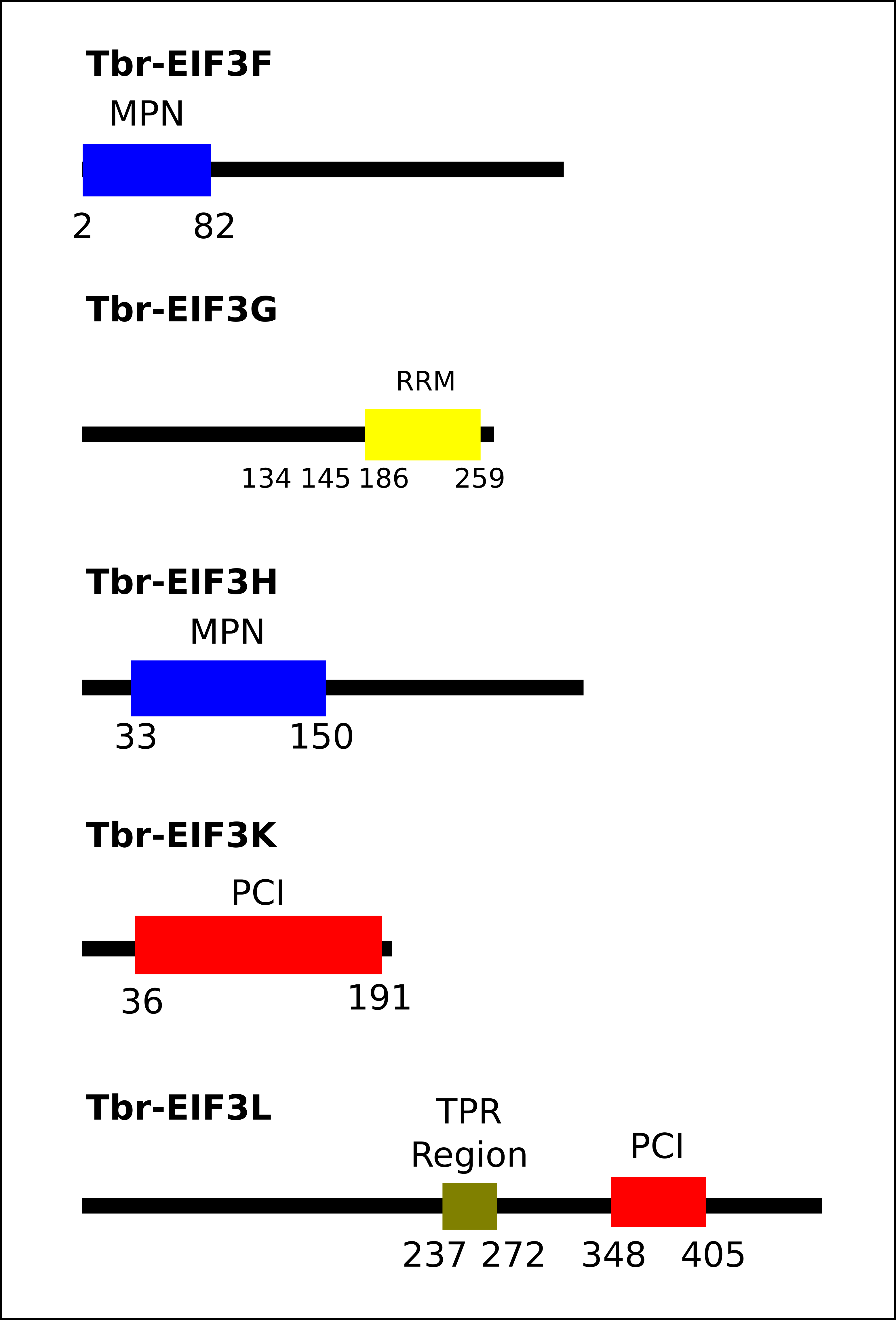


Rezende et al. – Figure S8

**Hsa-eIF3g** MP--TGDFDS KPSWADQVEE E-------GE DDKCVTSELL KGIPLATGDT SPEPELLPGA PLPPPKEVI- -------NGN IKTVTEYKID EDGKKFKIVR 83

**Cel-eIF3g** MA----PAPE VVSWAEAVE- ---------- ---------- ---------- ---------- -QDNAPHIQ- -----EGADG TRTETAFT-E VDGVRWKVVT 47

**Ath-eIF3g** MTIDSQQKTS KFRWGEM--- --------DE DEDLDF---- ---------- ---------- -LLPPKQVI- ----GPDENG LKTTIEYKFN DEENKVKITT 59

**Ani-eIF3g** MS----KLGN RADWAD---- --------DE EFDDPS---- ---------- ---------- -ALPPQQIT- -----TNKDG TKTIVSYRFN DEGKKVKVTR 53

**Spo-eIF3g** MS-----SSK SLDWAD---- --------DE DYG--T---- ---------- ---------- -GLPSIQTF- -----DNPDG TKTMIEFRID DNGKKVKVTR 50

**Sce-eIF3g** MS-------- ---------- ---------- ---------- ---------- ---------- -EVAPEEII- -----ENADG SRSIITYKIE D-GVKYKITQ 34

**Lmj-EIF3G** MS-------- --NWADAAQD EEQYQAAYGS DDN-DS---- ---------- ---------- -YSEHEEPK- -----PSWDT AKTEITFEVD ADGNRFEVMR 58

**Tbr-EIF3G** MA-------- --TWADDME- ----PIALGE DFG-GN---- ---------- ---------- -QLTPQEAKA LADKEAAWKN AKVVTETITD AENKQYEIVK 59

**Hsa-eIF3g** TFR---IETR KASKAVARRK NWKKFGNSE- -FDPPGPNVA TTTVSDDVSM TFITS----K EDLNCQEEED PMN------K LKGQKIVSCR IC--KG-DHW 165

**Cel-eIF3g** QFK---VINK RVPKVVADRK KWVKFGSCK- -GEPAGPQVA TTYVAEEVDM QFTRN----R AGEQILDVQE DKQ------T AKTTSREHCR HC--KGNDHW 130

**Ath-eIF3g** RTRVRKLASA RLNKRAMERR NWPKFGDAA- -NEEAGSHLT MVSTEEILLE RPRA--PGTK A-----DESK ATGDGLSQLG KGGAVLMVCR ICHKKG-DHW 149

**Ani-eIF3g** RIK-TTVVRE HVNPQVAERR SWAKFGLEK- -GNAPGPSFD TTSVGENIVF RPSVNWKLQA A-----EAEK NGGEKGSVKD QLKDKKVKCR IC--SG-EHF 142

**Spo-eIF3g** VIR-KTVITE RVQHAVAERK KWKKFGKEA- -GKNSGVDAR TTSVGENVQL RLQLGWTTTK E-----EEQD EAALAAAKVK AKGSSVVRCR AC--KG-NHF 139

**Sce-eIF3g** KVK-EVKVLE KVHKSVAERK NWHKYGSEK- -GSPAGPSAV TARLGEEVEL RLSRNWK--Q A-----EEER IQKEKASLTK TG----LQCR LC--GN-DHM 117

**Lmj-EIF3G** KVR-SYHVDR PIT-MADIRA KLAHFGKGK- -GDQS----- -TLVSAEPPL ALEMGSVDQF E-----RESR AEVKRMIHEA SGIDVAV--- ----KD-EHL 135

**Tbr-EIF3G** RV-LQYRVDR EAT-PVDVRA KWKRFGRATN PADQK----- -DLVSRDPPI VLELGEVDPF E-----RMAR EEVMRLMNEV ERYTVEV--- ----KD-VHL 138

**ZINC FINGER MOTIF**

**Hsa-eIF3g** TTRCPYKDTL GP----MQKE LAEQLG--LS TGEKEKLPGE LEPVQATQNK TGKYVPPSLR DGASR----- RGESMQPNRR ADDNATIRVT NLSEDTRETD 254

**Cel-eIF3g** STHCPYKVMY Q-----LDEE ADA-----DK DTEKDRMAMG MRP------- ---------- ---------- --DGRQIDRN RSDENTCRVT NLPQEMNEDE 191

**Ath-eIF3g** TSKCPYKDLA APTDVFIDKP PTG------- --ESSTMSAA PGT------G KAAYVPPSMR AGADR----- SAVGSDM-RR RNDENSVRVT NLSEDTREPD 228

**Ani-eIF3g** TARCPFKDTM AP----VDEP TAGG----EA GDEDSPAAGA LGA------G TSSYVPPHLR KGAAG----- GGERMAGKYE KDDLATLRVT NVSELAEEGE 223

**Spo-eIF3g** TAQCPYKSII GP----VDEP PL------DA SPVSSRASGA LGE------- KGRYIAPHLR AGSGR----- ESGDSMFKRE RDDSATLRVT NLSDDTREEE 217

**Sce-eIF3g** TMNCPFKTIL SELSA-LEDP ATNEGGVEAA SEEKAGQVGG AGS------I PGQYVPPSRR AGARDP---- SSDAYRDSRE RDDMCTLKIM QVNENADENS 206

**Lmj-EIF3G** ----RVVREM EE-------- ---------- ---------- ---------- KAKKDPRVVR SGAGSTWGAI ASKST--DNR EQSTTGIRIR NLSDDITQEN 191

**Tbr-EIF3G** ARYAKVKEEQ ER-------- ---------- ---------- ---------- AAKEAAAPDD QGKERTWAAA RGDKTSVQHK EDTDRRLRIT NISDDISREE 200

**ZINC FINGER MOTIF RRM DOMAIN**

**Hsa-eIF3g** LQ-ELFRPFG -SISRIYLAK DKTTGQSKGF AFISFHRRED AARAIAGVSG -FGYDHLILN VEWA-----K PSTN- 320

**Cel-eIF3g** LR-DLFGKIG -RVIRIFIAR DKVTGLPKGF AFVTFESRDD AARAIAELND -IRMYHMVLK VEWT-----R PSN-- 256

**Ath-eIF3g** LM-ELFHPFG -AVTRVYVAI DQKTGVSRGF GFVNFVSRED AQRAINKLNG -YGYDNLILR VEWA-----T PRPT- 294

**Ani-eIF3g** LR-DLFERFG -RVTRVFLAR DRETQRAKGF AFISFADRSD AARACEKMDG -FGYRHLILR VEFA-----K RAT-- 288

**Spo-eIF3g** LR-DLFRRFG -GIQRVYLAK DKETGRAKGF AFVSYYDRDC AIKARDRLDG -YGWNNLILR CEFS-----K PRD-- 282

**Sce-eIF3g** LREELLFPFA -PIPRVSVVR NKETGKSRGL AFVTFSSEEV AEQALRFLDG -RGYMNLILR VEWS-----K PKVKE 274

**Lmj-EIF3G** LS-RIFEGRG -WITKNVRIP RGDNNQTRGF AFVIFEEAWM ADAAIK--EG KFHFKNVVLD VSRA-----E TRT-- 255

**Tbr-EIF3G** LY-NIFNTDE YRIDKLFLPT DGKTSNYRGF AFITFETPEQ AERCLSRTKG VARFKNTVMH IVRALPEGAK QRN-- 272

**RRM DOMAIN**

Rezende et al. – Figure S9

**Hsa-eIF3i ---------** MKPILLQGHER SITQIKYNRE G--------- -DLLFTVAKD [ 4 AAs ] V--WYSVNGE RLGTYM---- [ 4 AA ] --AVWCVDAD 61

**Cel-eIF3i** **---------** MRPLSLKGHER ALTRVRFNRE G--------- -DLTFSCAKD [ 4 AAs ] V--WYTENGE RIGSYD---- [ 4 AA ] --AVWDIDVS 61

**Ath-eIF3i** **---------** MRPILMKGHER PLTFLRYNRE G--------- -DLLFSCAKD [ 4 AAs ] L--WFADNGE RLGTYR---- [ 4 AA ] --AVWCCDVS 61

**Ani-eIF3i** **---------** MRPILLSGHER SLNQIKFNRD G--------- -DLIFSVAKD [ 4 AAs ] A--WWSANGE RLGTYN---- [ 4 AA ] --AIWTVDVS 61

**Spo-eIF3i** **---------** MRPIILQGHER PLTQIKYNHD G--------- -DLLFSCAKD [ 4 AAs ] V--WFSHNGE RLGTYE---- [ 4 AA ] --AIWTCDIN 61

**Sce-eIF3i** **---------** MKAIKLTGHER PLTQVKYNKE G--------- -DLLFSCSKD [ 4 AAs ] V--WYSLNGE RLGTLD---- [ 4 AA ] --TIWSIDVD 61

**Lmj-EIF3I** [ 69 AAs] LEGVALHGHMK GVTMLKFNRD G--------- -DLLFSSAKD [ 4 AAs ] ACCWQVKTGK LFGSYT---- [ 12 AA ] --AMVALDVN 140

**Tbr-EIF3I** [ 2 AAs] IQGMALHGHMK PVTMIKFNRE G--------- -DLLFSTAKE [ 4 AAs ] V--WYTKTGE RLGTYD---- [ 3 AA ] --AISACDVN 62

**Tva-eIF3i** [ 24 AAs] TESVLFTPGER ACTHACLDST G--------- -HLAF-VVSD [ 4 AAs ] G--YFIPFGT NFKIYS---- [ 4 AA ] --AITDIDIE 84

**Gdu-eIF3i** [ 9 AAs] AHVFSLHGHTR PITKLRYTPD G--------- -DYIITGSTD [ 4 AAs ] M--WTSTNGQ FVQTFGPI-- [ 13 AA ] GIAVSDFTVS 83

**Mja-eIF3i** [ 2 AAS] MRMVFVENKIN GVENIRQEID N[ 69 AAs] WDLSIKDIKD [ 60 AA ] I--WESKVGE RMWGFSLKDN [ 89 AA ] WGCVYALDTN 270

**Hsa-eIF3i** WDTK------ [ 4 AAs ] GSADNSCRLW DCETGKQ-LA LLKTN--SAV RT-CGFDFGG NIIMFS-TDK QMGYQCFVSF FDLR----DP S-----QIDN 135

**Cel-eIF3i** WDTT------ [ 4 AAs ] ASGDLTVKIW DAELGNC-LY TINHQ--TPM KS-CGFSYSG NLVCFT-TQK MTKNLSTFQV RDLR----DS S-----QMVE 135

**Ath-eIF3i** RDSS------ [ 4 AAs ] GSADQTAKLW DVKSGKE-LF TFKFN--APT RS-VDFAVGD RLAVIT-TDH FVDRTAAIHV KRIA----ED P-----EEQD 135

**Ani-eIF3i** PNTQ------ [ 4 AAs ] GSADNTVRLW NVKTGEC-IK VWDFP--TAV NV-WGF---- ---------- ----LGTIAV LDI------- NYGENLTEQA 120

**Spo-eIF3i** KSST------ [ 4 AAs ] GAADNTMRLW DVKTGKQ-LY KWEFP--TAV KR-VEFNEDD TRILAV-TEE RMGYAGTVTV FRVPI---SE S-----DAAA 136

**Sce-eIF3i** CFTK------ [ 4 AAs ] GSADYSIKLW DVSNGQC-VA TWKSP--VPV KR-VEFSPCG NYFLAI-LDN VMKNPGSINI YEIE----RD SATHELTKVS 140

**Lmj-EIF3I** REST------ [ 4 AAs ] ASAGEEVLLW SVESGAL-LG SVSRS--LSS GASVGFSHDD TLMMVA-TKG RSSTNSAIQV YNVPFTVPKA GEDIAPVKTP 224

**Tbr-EIF3I** NYST------ [ 4 AAs ] GGMDFKAKLW CVETGEE-LA NIMLR--TPA RA-VGFSHDD NLLMVS-TSR KMGQKSAVQL YNLPFLPPKD GYSIHPVNTV 145

**Tva-eIF3i** PENK------ [ 4 AAs ] VGNSMHIIFH EVETGNI-LL DELTD--KMH AA-CCFGPRN MHFFATVTSK QMKQEIVLTG YHFVEKRGKD D-----YPID 162

**Gdu-eIF3i** SDGE------ [ 4 AAs ] SHVLDFIALY KVTSDRAYIR TYKFPEITAY SA-IQYSRDG RRLFVG-GRL IMKSKAGILV LDAS--DPKD V--------- 158

**Mja-eIF3i** LYSKIQRVRP [ 220 AAs] GCVSGHVYAI DIKTGKK-LW EFKAEDTV-- ---WGLSIKD DIVVLG-CGN IFESIVMLKN GKIL----EE GYAYALDINT 579

**Hsa-eIF3i** NEPYMKIPCN D--SKITSAV WGPLGECIIA GHESGELNQY SAKSG---EV LVNVKEHS-- -RQINDIQLS RDMTMFVTAS KDNTAKLF-- ---DSTTLEH 222

**Cel-eIF3i** GGESFFYSQF D--VNATTAL FTQMDDLVTI GFESGLLQQY DLRNP---DT PIHTNESV-H RYSVQDLQLS PRGDFLISAS RDKTAALL-- ---DVNDLKK 224

**Ath-eIF3i** AESVLVLHCP DGKKRINRAV WGPLNQTIVS GGEDKVIRIW DAETG---KL LKQSDEEVGH KKDITSLCKA ADDSHFLTGS LDKTAKLW-- ---DMRTLTL 227

**Ani-eIF3i** EEPSLRITCT E--SKATVAG WSYMGKYIIA GHEDGSVSQY DGKVRRNACQ LENVQAHE-F DHQINDIQFS ADRTYFITAS KDKSAKLM-- ---STRNLAI 212

**Spo-eIF3i** ETPLYVITTR E--SKATVAG WSYLSKFLFT GHEDGSVSRY DAITG---EF VESKQVHN-S GSTITDLQFY PDRTYFITSC KDTTAKAI-- ---DVDSFEV 225

**Sce-eIF3i** EEPIHKIITH EGLDAATVAG WSTKGKYIIA GHKDGKISKY DVSNN--YEY VDSIDLHE-- -KSISDMQFS PDLTYFITSS RDTNSFLV-- ---DVSTLQV 230

**Lmj-EIF3I** FTTFSTF-ET P--DTITWAA WGPTNETIYY SEG-GYMNIL DVEAN---KV IRSRQIHEDE NEVINRFSWD PNYLALATAS TDKTSHLI-- ---DFRDLAT 312

**Tbr-EIF3I** FNPCTEFVSE N--DDVTFAI WGPTNDTIYY STSDGSVAIL DVETM---ST VCTHKPHE-- -ETINRISFD SNYYTLITAS KDKTARLL-- ---DSRDLSV 232

**Tva-eIF3i** MSNVMFKYKF D--CVVNSIR W-PKADMILA GDVQGKVHVL TGLNN-DVPN VQIIDAHR-- -GPINAITMS FDNNFFATAS ADTTARLWKI PQKEIDQFEL 256

**Gdu-eIF3i** --KCTKIKCM S--EDVTCIA LSPLDDYLVV GFASGVVQLF DAETL--EPY SKMKDPIVVG NGQIMSIQLY NN-SMFTICC SDKKIVIL-- ---GAKTLTV 246

**Mja-eIF3i** GREIWRSKIK H-----DVRS LSIKDDIVVL GCKKGYILAL DINAG---NM LWEFKAKS-- GKSIRNLSIK NDILLF---G CDNYLYAL-- ---DIDTGRE 661

**eIF3b BINDING REGION**

**Hsa-eIF3i** QKTFRTERPV NSAALSPNYD H-VVLG--GG QEAMDVTTTS TRIGKFEA-- [ 1 AAs ] FFHLAFEEEF GRVKGHFGPI NSVAFHPDGK SYSSGGEDGY 308

**Cel-eIF3i** LKQYKSERPV NSACISPNRD H-ICLG--GG EDAMQVTQTS VSAGHFEA-- [ 1 AAs ] IYHMVFEEEF ARFKGHFGPI NTMAWHPSGT IIATGGEDGY 310

**Ath-eIF3i** LKTYTTVVPV NAVSLSPLLN H-VVLG--GG QDASAVTTTD HRAGKFEA-- [ 1 AAs ] FYDKILQEEI GGVKGHFGPI NALAFNPDGK SFSSGGEDGY 313

**Ani-eIF3i** LKTYVADTPL NSATITPKKD Y-VILG--GG QAAMDVTTTS ARQGKFEA-- [ 1 AAs ] FYHKVFEDEI GRVRGHFGPL NTVHIHPAGT AYASGGEDGY 298

**Spo-eIF3i** IKTYLTDTPL NTSSFTPVQD F-VILG--GG QEARDVTTTA ARQGKFEA-- [ 1 AAs ] FYHAILEEEL GRVKGHFGPI NTIAVHPKGT GYASGGEDGY 311

**Sce-eIF3i** LKKYETDCPL NTAVITPLKE F-IILG--GG QEAKDVTTTS ANEGKFEA-- [ 1 AAs ] FYHKIFEEEI GRVQGHFGPL NTVAISPQGT SYASGGEDGF 316

**Lmj-EIF3I** IQVYRSDVPV NDVSISPNAD H-VILG--GG MDAASVTTQG G-QSIFEV-- [ 1 AAs ] FFHKVHGHQL GQLRCHFGTI NAMSFHPDGR GFASASYDGL 397

**Tbr-EIF3I** VQTYTSDVPV NDASISPRGD H-VIIG--GG MDAQDVTTQG G-QTTFEV-- [ 1 AAs ] FYHKVHEKQL GQVRCHFGTI NSVCFFPDGR GFASGAFDGL 317

**Tva-eIF3i** IGTYNHSFQI SCAAISPKEP V-IVLA--ST ADHSEVARIN SGSTDFTI-- [ 1 AAs ] FFHTIFQEEF ASMKVHKSPI NWVAFTSDGF SLITTSAEGT 342

**Gdu-eIF3i** LKKVSTEYPV HCCAIHPNIP NLMVYA--GG MDSKIVTQTT HIENTFKL-- [ 1 AAs ] FIDIAQDVKL GSIQAHVGPV HDLAFNPNGE DLVSCSEDST 333

**Mja-eIF3i** LWRFKAEGEV KSLSI--KKD N-VLLGCRGG YVYLLDINTG EKMERFKVVG [ 60 AAs ] ALDINTGEEI WKFKTESAVL D---LSIKDN IVISGCKRGH 805

**eIF3b BINDING REGION**

**Hsa-eIF3i** VRIHYFD--P QYFEFEFEA- ---------- --- 325

**Cel-eIF3i** IRIQEFD--E DYLGFTYDF- ---------- --- 327

**Ath-eIF3i** VRLHHFD--S DYFNIKI--- ---------- --- 328

**Ani-eIF3i** VRVHHFD--K PYFDFMYEVE REQLRK---- --- 322

**Spo-eIF3i** VRVHFFD--K NYFDFKYTL- ---------- --- 328

**Sce-eIF3i** IRLHHFE--K SYFDFKYDVE KAAEAKEHMQ EAN 347

**Lmj-EIF3I** IKMYRFG--D SYDST----- -PGAQPLWT- --L 419

**Tbr-EIF3I** VKLHRFD--D KYSST----- -PGAIPLWTP EGI 342

**Tva-eIF3i** FQVIHLC--G DYAESIIEQR RKMKEIEAIT D-- 371

**Gdu-eIF3i** TFLIKVG--K DFKNYNFIK- ---------- --- 350

**Mja-eIF3i** VYALDFNIIK NY-------- ---SIIQKIK QVL 827

Rezende et al. – Figure S10

**Hsa-eIF3j** M [39 AAs] EGEDEDEDVK DNWDDDDDEK KEEA--EVKP EVKISEKKKI AEKIKEKERQ QKKRQEEI-K KRLEEPEEPK VLTPEEQLAD KL---RLKKL 123

**Cel-eIF3j** M--------- ---------- SDWEDDDFEP EVSTFKRAEP APEPVKIVDA PPPPQKA--- ---------A PAAPKTLKAA PTFAMESLGR ELTSAEKEAI 69

**Ath-eIF3j** M [32 AAs] DEDVDENEIK DSWEDDDDEP AQPP--VINP APEKAPKKAA PKTVEKK--- ---------G KAVEVPKEAP KEKPLDPIAE KL---RQQRL 96

**Ani-eIF3j** M [22 AAs] FDDEEEEDVL DSWDAAEDSE VERE--KAAK AAEAKAKAEA EAAANKKSKA QRIQEKKA-Q RKADADAEDS DDSDEDEAER RA---RLRKT 107

**Spo-eIF3j** M [37 AAs] EEDEDEEENK ESLQNDSHSV SQKS------ ----SSSSQN DQGSNKMTRI QQKIQERNFE KAIKASEAAA KEESLESSKE AM-----RQA 113

**Sce-eIF3j** M [24 AAs] AEIGDDEPVM QSWDAEEEEK K--------- -PAPKPKKEQ PKKVKK---- ---------G KESSADRALL DIDTLDEKTR KE---LIKKA 88

**Lmj-EIF3J** M [13 AAs] YEDDEYDDAA DDWEAEAAEE EQRA--VAEE AAR------Q ARLEKKL--- ---------A TRAPVKKEEE EEAVPEDVER AM---ADMR- 80

**Tbr-EIF3J** M [1 AAs ] EDYHEYDDAA DDWEVEAQQG E-----AAEE EALKRKLREQ QALLEKERQR ---------R RARASARTEE EELLPADVAR AL---EDMKS 73

**Gdu-eIF3j** M [4 AAs ] FGSSSEGSEP EAWESNAMKL SKQN--EEKE IAEQKRRAEE EAARNREKIR ---------K RERDSFTTGA PELSETAKAR LD---EKRR- 80

**Hsa-eIF3j** QEESDLELAK ETFGVNNAV- ---------- ----YGIDAM NPSSRDDFTE FGKLLKDKIT QYEKSL---Y YASFLEVLVR DVCISLEIDD --------LK 197

**Cel-eIF3j** QKKNDLALAR DLFGDDDSAE [ 9 AAs ] ---------- NIMSKADFEY WGERVGGFLA SRSKAS---C YGDMIGKLLT SVTDEMTPAE --------IL 147

**Ath-eIF3j** VEEADYRATA ELFGVKDDD- ---------- ----KNLDMF IPKSESDFLE YAEMISHRIK PYEKSY---H YIALLKTIMR LSLTNMKAAD --------VK 170

**Ani-eIF3j** EKDADLKHAE DLFGDIDLNR [ 32 AAs ] --DLSAMPLF KPATKDQFTR VTTTLIPLLT TQSKKP---Q YALWAQDFIK QLVKDLPSGD --------VK 206

**Spo-eIF3j** EIDSDLANAM DLFDIVDKNS [ 6 AAs ] ----KQADQR QLKTKADYAA FQADILKKVK NCQTTA---E YNNFVQDLIP LLLTGLNATN --------LK 194

**Sce-eIF3j** EMESDLNNAA DLFAGLGVAE [ 26 AAs ] --PIETHPLF NAETKREYQD LRKALTAAIT PMNKKSPLNY SSSLAIDLIR DVAKPMSIES --------IR 194

**Lmj-EIF3J** QVANDIASGS ALLSGGDSE- ---------- ----ELIGNH KLASDADVER VGTMIADRLT SFSDSG---H YDKLIVEVFQ RLTNHLTSTH LLNEEADRVH 162

**Tbr-EIF3J** TASS---AGE ALNVVKDSEN [ 2 AAs ] ----KLIADM PVNTAAEAEA LGVVLADRLL SFSESP---H FNSILSVVFR DIAREFKSGN ALDTLRT-IK 156

**Gdu-eIF3j** -YEQNIVAAS NMLRDNDSSD [ 40 AAs ] SSGPQSIEEM IPASIEQFDA FADAIVKKLV STYKNDNDVL YMHCLKRIVE GALSPLYHED --------AK 201

**Hsa-eIF3j** KITNSLTVLC SEKQKQEKQ- ---------S K-----AKKK KKGVVPGG-G LKATM----- ------KDDL ADYGGYDGGY VQDYED-F-- M 258

**Cel-eIF3j** KMVTFLQQIS AAKKTAEKTK AKATTAAKPA A-----AANK KNAKATLKVT ---------- ------KGND SMYDDYG-DD QDDYDD---- Y 212

**Ath-eIF3j** DVASSITTIA NEKLKAEKE- ---------A A-----AGKK KGG------K KKQLIVDK-- ------ANDD LVAGPYD--- AMDDFD-F-- M 226

**Ani-eIF3j** KIASSLTTLS NEKMKEERA- ---------A D-----KGNK KTKAAK---- TKVSLVTS-- ------RENK IETNSYD-DD GLDDDD-F-- M 266

**Spo-eIF3j** AVQKSVNKLV VNKEQQEKTQ SKRGAAAPAA KPVSTAAPSK KGGKPTVNVN SKKTVADKS- ------AYED YIEDEYD-DY ADDFDD-F-- M 274

**Sce-eIF3j** QTVATLNVLI KDKEREERQ- ---------A R-----LARV RGGTATGG-A GKKKVKGKTN LGGAFKKDQD FDLDGPD-DF EFGDDD-F-- M 265

**Lmj-EIF3J** RLREELKRAA KAKLKPVEKA PVTQSG---- ---------- ---------- ---------- ------LDLD NFEDRGG-AA VADNED-ETG W 211

**Tbr-EIF3J** DKVSNASKVC EGKMKQTKGD KREQPG---K E-----GASA KGGSSAGDA- ---------- ------LGLD TIGDEGGAAN VPDGDDNF-- M 220

**Gdu-eIF3j** ELSDVCARIS VEMYRKEAEK KK-------K K-----GKAQ KGGSHATG-K SQVYV----- ------GGRD AKYQKVD--- -DDYED-F-- I 261

Rezende et al. – Figure S11

**eIF3k alignment**

**Hsa-eIF3k** M-----AMFE QMRANVGKLL KGIDRYNPEN LATLERYVET QAKENAYDLE ANLAVLKLYQ FNPAFFQTTV TAQILLKALT NLPHTDFTLC KCMIDQAHQE 95

**Cel-eIF3k** M-----S-FE KLQKELHEAI EGVNRYNPEN VADLAACVQA MVNENKYDKD IVLTILKLYQ LNPEKYDEAV VRQVLLKTLM VLPSSDFALA KCLIDTNRLG 94

**Ath-eIF3k** MGVEIQSPQE QSSYTVEQLV -ALNPFNPEI LPDLENYVN- -VTSQTYSLE VNLCLLRLYQ FEPERMNTHI VARILVKALM AMPTPDFSLC LFLIPERVQM 97

**Ani-eIF3k** MGVTFDK-CE TRPANIDAIL NGLDRYNPET TTVFQDYVAQ QCEDRTFDCY ANLALLKLYQ FNPHLLQAET VTNVLVKALT VFPSPAFSLC LALLPAHTQP 99

**Lmj-EIF3K** M--------- ---------- ---------- TRDDGTQVAQ LLSEGKYNLP MFLRYLKASS MEGDQPDKSL LLGILLQSLA RFQTSDFTAC MCLVPSHVQD 71

**Tbr-EIF3K** M--------- ---------- ---------- NQDLDKELRE ALQQDKYDLQ LFLRFLKSYV VSGSQPEKQL LLGILLQALP RFHTSDFSAC ISLISSHVQD 71

**PCI DOMAIN**

**Hsa-eIF3k** ---------- -------ERP IRQILYLGDL LETCHFQAFW QALD------ ---------- -ENMDLLEGI TGFEDSVRKF ICHVVGITYQ HIDRWLLAE- 160

**Cel-eIF3k** ---------- -------SQE LRRIFDLGAV LESCNFAVFW KLVKGAYKPT TNPNEPFKVP GEVPKMIKPM VGFEDAVKHY ACRVISVTFQ KIEKKMLSR- 176

**Ath-eIF3k** ---------- -------EEQ FKSLIVLSHY LETGRFQQFW DEAA------ ---------- -KNRHILEAV PGFEQAIQAY ASHLLSLSYQ KVPRSVLAEA 163

**Ani-eIF3k** FQASDAEAQA AAQTSDFVES IQKLARLSTL LESAQYTQFW STLN------ --------SD DLYADLVADV AGFEELVRIR IAVEVGKTFR EIPAEVLEQW 185

**Lmj-EIF3K** ---------- ---SPSVEKE LNYIYGLENL LSCGLFARFW TQWS------ ---------- -SVKEHLPES FHFEARVRTS ILETICITME SIPTEKLATY 141

**Tbr-EIF3K** ---------- ---ASYIEKD IILIYDLENY LSSGNFPQFW QVWR------ ---------- -ESEGTLPAR PSFEPNMRAA ILTVIGCTLG HIQTKDLSVY 141

**PCI DOMAIN**

**Hsa-eIF3k** --MLG-DLSD SQLKVWMSKY GWS----ADE SGQI-FICSQ E---ESIKPK NIVEKIDFDS VSSIM----- -ASSQ----- ---------- ----- 218

**Cel-eIF3k** --LLG-GASD KEVTALAQSF GWE----AKE NGDVFFVANH E---GTIKTR NIDEKIQFPH VADLL----- -TSIQP---- ---------- -PLTL 240

**Ath-eIF3k** VNMDG-ASLD KFIEQQVTNS GWI----VEK EGGSIVLPQN EFNHPELK-K NTGENVPLEH IARIF----- ---------- ---------- -PILG 226

**Ani-eIF3k** LDLRSREALE KFVAEVCS-- -WE----VDK SGANTVIKVP TNKENEARSE VKSERVGVDM FGRVI----- -RRGSSRLHE RIDKRYPHRT QPFSF 267

**Lmj-EIF3K** LAV-SPDQVQ KVVHNAMKDS EDRDMKVMAY DSGSVVFHRN RFNYPQAGAA Q--DAIRFTD VSSVIHNDVP RRGAAVAADE TRDVRARTWA RMADE 233

**Tbr-EIF3K** LGLKSGEKLE EVLREAARIA GDA-VQLVGA DEQAVVFQKS VFNAPESDSN Q--ELKRFSD TVTIV----- ---------- ---------- ---SL 205

**PCI DOMAIN**

**eIF3l alignment**

**Hsa-eIF3l** ---------- M SYPADDYES- ----EAAYD- [ 30 AAs ]VIPEVIKNF IQYFHKTVSD [20 AAs] QKVYEIQDIY ENSWTKLTER F-------FK 108

**Ath-eIF3l** ---------- M A-SSNEYEEG PGERESGYD- [ 1 AAs ]MVPDSVKSF VSHMYRHIR- [1 AAs ] KNVYEIHQMC ETSFQSISER L-------FK 63

**Ani-eIF3l** ---------- M S-----YEE- ----RANAH- [ 11 AAs ]------EAL VNDYREQVN- [1 AAs ] DGMSELDRTT SLGGGSQTQD L-------QA 58

**Cel-eIF3l** [20 AAs] L AREKELRKEA ERQLKVLLDK [ 50 AAs ]KATDIVLEY LAHFSKMIA- [1 AAs ] NNLLEIQNCY YDRLETVQKQ L-------PP 134

**Lmj-EIF3L** [89 AAs] M A--------- -------TN- [ 4 AAs ]DIPRDVYQF FRGLNRAVE- [1 AAs ] RDAAAMHDLY ESQFDALTKN YYMAGHGQFR 147

**Tbr-EIF3L** ---------- M A--------- -------SN- [ 4 AAs ]HIPDEMFNF FNSLNTAVD- [1 AAs ] GDISSFHNLY ENIFPSHLSK YYAAEQGEFR 58

**Hsa-eIF3l** NTPWPE---- AEAIAPQVGN DAVFLILYKE LYYRHIYAKV SGGPSLEQRF ESYYNYCNLF NYILNADGPA PLELPN-QWL WDIIDEFIYQ FQSFSQYRCK 203

**Ath-eIF3l** DTPWPS---- VEAIAPYVDN DHVFCLLYRE MWFRHLYARL S--PTLKQRI DSYDNYCSLF QVVLH--GVV NMQLPN-QWL WDMVDEFVYQ FQSFCQFRAK 154

**Ani-eIF3l** QL-------- AAAATP---- -----LEYQ- ---------- ---ATLETKF ASYDNYCSLF HYILNSDGPV ELEVPSYYWA WDVIDEFIYQ FESFCRYRNR 127

**Cel-eIF3l** SFTWPDEITV RKSISP---E NQDFMVLYKE LKYRRLN--- ---AEARQNK DMLENYQSIF SIL-----PG KLNLPD-YWI LDIMEEYVFQ IQT------- 212

**Lmj-EIF3L** SWPALR---- LEQVSSCFRG NHMAELVYSF LFYKHLFMDN RS-VRAPDAE GAWKTYSELL PML------K SYELPN-WML WDIFDEFLYQ MTVVYQKVFV 236

**Tbr-EIF3L** PLPVLQ---- RMEVAECF-G NDTAGKLYSF LCFKHLFTD- RD-VTAEDAK VSWRTFCDLF VSL-----PG SCDIPN-WFL WDIFDEFLFQ MTVVYQKRFA 146

**Hsa-eIF3l** [ 18 AAs ] WNVHSVLNVL HSLVDKSNIN RQLE------ --VYTSGGDP ESVAGEYGRH SLYKMLGYFS LVGLLRLHSL LGDYYQAIKV LENIELNKKS 303

**Ath-eIF3l** [ 18 AAs ] WNVYGVLNFL QALVEKSCII QILEHDKDGL EFTETDGYDF S------GGS NVLKVLGYFS MVGLLRVHCL LGDYHTALKW LQPIDITQPG 256

**Ani-eIF3l** [ 20 AAs ] WGCYSVLNVL YSLIQRSQIN EQLA------ --AIKRGEDP LAFAGEYGSR PLYKMLGYFS IIGLLRVHCL LGDFSLALKT LDDIEMNKKA 229

**Cel-eIF3l** [ 8 AAs ] SNKDAALNTL ISLASQPEAN LKTL------ ---------- ---------- ---------S LLGLIRMHIF LQDHQSTSQI LDQMPPEIQG 275

**Lmj-EIF3L** [ 4 AAs ] WAVPEVSRLL NEVIDNSRLL ELVK------ ------QPDF LEDIHRGPNT GA--LSGFYA IVTKSKLNVL LGDYYSALTD LEPLDVYNKG 315

**Tbr-EIF3L** [ 4 AAs ] WSVTEAVQMM EKVISESG-I EEVM------ ------ESDK ADDITKSGEN HVRWMSGFFG IITVAKINVL LGDYNSALSV LKPLDIYGRG 226

**Hsa-eIF3l** --MYSRVPEC QVTTYYYVGF AYLMMRRYQD AIRVFANILL YIQRTKSMFQ RTTYKYEMIN KQNEQMHALL AIALTMYPMR --ID--ESIH LQLREKYGDK 397

**Ath-eIF3l** --VYTSVIGC HIATIYHYGF ATLMLRRYVD AVREFNKILL YIFKTKQYHQ KSP-QYEQLL KKNEQMYALL ALSLSLCPQT KLVD--ESVN SQLRDKYGEK 351

**Ani-eIF3l** --MFARVMAA HFTTYYYVGF SYMMMRRYAD AIRMFSHILV YVSRTKN-FQ KGGNSYDAIA KKNDQMYALI AICVALHPTR --LD--DTIH SALREKYGEQ 322

**Cel-eIF3l** TETMIRVT-- -----YQIGF AYLIIGRFAE SVDRFLKVLS SSVEQAEKFK ---------- -----INALP CIAYCISNSS KELEIPSEIS KHVESTYPNQ 353

**Lmj-EIF3L** RAVLTRVSPC AVSVFYHIGF SYLMIHRFED ASNAFRRCVT VKLNGRRFSE R-------VQ QDAAYMYVCA RVLGGMPINN --LT--SYLD SRKVAAFEDD 404

**Tbr-EIF3L** KKILAEVAPA NVSLMYYVGF SYLMLRRYAD ASRVFRQSLS AKVSSRKFSE R-------VR LDCAFMHVVS CILCGTQPDN --LS--WLMD SRKLQVLEDD 315

**TPR REGION**

**Hsa-eIF3l** MLRMQKGDPQ ---VYEELFS YSCPKFLSPV V-PNYDNVHP NYHKEPFLQQ LKVFSDEVQQ QAQLSTIRSF LKLYTT-MPV AKLAGFLDLT EQEFRIQLLV 493

**Ath-eIF3l** MMRMLRSDDE AFGIYDELFS YACPKFITPS APPTFEEPLV NYNQDAYRLQ LKMFLYEVKQ QQLLSGVRTF LKVYSS-ISL AKLANYMEVD EPTLRTILLT 450

**Ani-eIF3l** LNRLQHGGPE ALPLFEELFR SACPKFISPT -PPDFENPAL NV--DPVDHH TAIFMDEVKN TLYNPTIRSY LKLYTT-MDL KKLAGFLEVE PEKLRSWLLI 418

**Cel-eIF3l** MKDWKEAKIE ---TFLSFFA ECS-SFI--- -PQLPRNNKL SLHWKSQNLK SMVF------ ---------Y LQRVTTWKPL RNLKGLLKI- -----CPVLP 425

**Lmj-EIF3L** RESLRTGDEE ---RFRDVFD RCSPKFLT-- VPPSDGSPVK GS--EGRELQ ARMFRRAVQQ QQDIIKLRGY FKVYQN-TKM NLLETLLEVD DG--YAPLFA 494

**Tbr-EIF3L** KELLATGDEE ---RFRDVFD RCSPKFLA-- VPPVPPTVCK GM--EGKELQ ARLFLRAVKQ QQDTIKLRVY LGVYQT-TTT ELVKTVLDVN DG--LVPLFA 405

**PCI DOMAIN**

**Hsa-eIF3l** FKHKMKNLVW TSGI--SALD GEFQSASEVD FYIDKD---- -MIHIADTKV ARRYGDF--- -----FIRQI HKFEELNRTL [7 AAs] 564

**Ath-eIF3l** YKHK------ THSV--DS-D GRIISNADID FFINND---- -MIYVVESKP AKRYGDF--- -----FLRQI AKLEGVINDM [6 AAs ] 514

**Ani-eIF3l** NKQRSRQVRW VEG---GLLE GEPVNANDLD YALEND---- -LIHVSETKA GRRLVDW--- -----YLRNL A--------- [3 AAs ] 476

**Cel-eIF3l** LEARATSLL- ---------- -PVSTSSDID YFCQYDSFLN RLVTVKPSKL TKIFQDFPDA NRQKTLVFLI MKYHKILQTV [66 AAs] 558

**Lmj-EIF3L** LKMRSRQLV- HDGVTADLLQ GVFTSSSEFD CIVQDD---- -NVEVVPSMT FGGIEQK--- -----LLNKI KNTQRDIEVA [73 AAs] 633

**Tbr-EIF3L** MKLTSRQLV- HDGVSADLHS GTYVVRAALD CTVEGD---- -NICVVQKSS YRTIESK--- -----YF--- ---------- [30 AAS] 488
