# Supplementary material for: The translation initiation complex eIF3 in trypanosomatids and other pathogenic excavates – identification of conserved and divergent features based on orthologue analysis
Source: BMC Genomics. 2014 Dec 23;15(1):1175. doi: 10.1186/1471-2164-15-1175 (PMC4320536; doi:10.1186/1471-2164-15-1175)
Supplement: Supplementary file 2 — Additional file 2: Table S1: Accession numbers for the various protein sequences used. (DOCX 27 KB) [file 12864_2014_6982_MOESM2_ESM.docx]

| **TABLE S1: ACESSION NUMBERS FOR PROTEINS INCLUDED IN THE FIGURES** | | | |
| --- | --- | --- | --- |
| **Protein** | **Accession** | **Data Base** | **Organism** |
| **eIF3a** | | | |
| Hsa-eIF3a | gi_4503509 | Gene Bank | *Homo sapiens* |
| Cel-eIF3a | gi_17552890 | Gene Bank | *Caenorhabditis elegans* |
| Ath-eIF3a | gi_15233360 | Gene Bank | *Arabidopsis thaliana* |
| Ani-eIF3a | gi_145229123 | Gene Bank | *Aspergillus niger* |
| Spo-eIF3a | gi_19113171 | Gene Bank | *Schizosaccharomyces pombe* |
| Sce-eIF3a | gi_6319553 | Gene Bank | *Saccharomyces cerevisae* |
| Lmj-EIF3A | LmjF.17.0010 | TriTrypDB | *Leishmania major* |
| Tbr-EIF3A | Tb927.7.6090 | TriTrypDB | *Trypanosoma brucei* |
| **eIF3b** | | | |
| Hsa-eIF3b | gi_33239445 | Gene Bank | *Homo sapiens* |
| Cel-eIF3b | gi_71998426 | Gene Bank | *Caenorhabditis elegans* |
| Ath-eIF3b | gi_18421140 | Gene Bank | *Arabidopsis thaliana* |
| Ani-eIF3b | gi_145229477 | Gene Bank | *Aspergillus niger* |
| Spo-eIF3b | gi_19115440 | Gene Bank | *Schizosaccharomyces pombe* |
| Sce-eIF3b | gi_398366379 | Gene Bank | *Saccharomyces cerevisae* |
| Lmj-EIF3B | LmjF.17.1290 | TriTrypDB | *Leishmania major* |
| Tbr-EIF3B | Tb927.5.2570 | TriTrypDB | *Trypanosoma brucei* |
| Tva-eIF3b | TVAG_333940 | TrichDB | *Trichomonas vaginalis* |
| Gdu-eIF3b | GL50803_15495 | GiardiaDB | *Giardia duodenalis* |
| **eIF3c** | | | |
| Hsa-eIF3c | gi_153791492 | Gene Bank | *Homo sapiens* |
| Cel-eIF3c | gi_17509197 | Gene Bank | *Caenorhabditis elegans* |
| Ath-eIF3c | gi_15228840 | Gene Bank | *Arabidopsis thaliana* |
| Ani-eIF3c | gi_317034913 | Gene Bank | *Aspergillus niger* |
| Spo-eIF3c | gi_63054571 | Gene Bank | *Schizosaccharomyces pombe* |
| Sce-eIF3c | gi_6323969 | Gene Bank | *Saccharomyces cerevisae* |
| Lmj-EIF3C | LmjF.36.6980 | TriTrypDB | *Leishmania major* |
| Tbr-EIF3C | Tb927.10.8270 Tb927.10.8290 | TriTrypDB | *Trypanosoma brucei* |
| Tva-eIF3c | TVAG_184380 | TrichDB | *Trichomonas vaginalis* |
| Gdu-eIF3c | GL50803_24279 | GiardiaDB | *Giardia duodenalis* |
| **eIF3d** | | | |
| Hsa-eIF3d | gi_4503523 | Gene Bank | *Homo sapiens* |
| Cel-eIF3d | gi_17554522 | Gene Bank | *Caenorhabditis elegans* |
| Ath-eIF3d | gi_15233469 | Gene Bank | *Arabidopsis thaliana* |
| Ani-eIF3d | gi_145250805 | Gene Bank | *Aspergillus niger* |
| Spo-eIF3d | gi_19115537 | Gene Bank | *Schizosaccharomyces pombe* |
| Lmj-EIF3D | LmjF.30.3040 | TriTrypDB | *Leishmania major* |
| Tbr-EIF3D | Tb927.6.4370 | TriTrypDB | *Trypanosoma brucei* |
| Tva-eIF3d | TVAG_062640 | TrichDB | *Trichomonas vaginalis* |
| **eIF3e** | | | |
| Hsa-eIF3e | gi_4503521 | Gene Bank | *Homo sapiens* |
| Cel-eIF3e | gi_17505378 | Gene Bank | *Caenorhabditis elegans* |
| Ath-eIF3e | gi_18410687 | Gene Bank | *Arabidopsis thaliana* |
| Ani-eIF3e | gi_145232947 | Gene Bank | *Aspergillus niger* |
| Spo-eIF3e | gi_19112159 | Gene Bank | *Schizosaccharomyces pombe* |
| Lmj-EIF3E | LmjF.28.2310 | TriTrypDB | *Leishmania major* |
| Tbr-EIF3E | Tb11.01.3420 | TriTrypDB | *Trypanosoma brucei* |

| **eIF3f, eIF3h and MPN containing proteins** | | | |
| --- | --- | --- | --- |
| Hsa-eIF3f | gi_4503519 | Gene Bank | *Homo sapiens* |
| Hsa-RPN7 | gi_25777615 | Gene Bank | *Homo sapiens* |
| Hsa-CSN6 | gi_34147637 | Gene Bank | *Homo sapiens* |
| Cel-eIF3f | gi_17532683 | Gene Bank | *Caenorhabditis elegans* |
| Cel-RPN8 | gi_17508685 | Gene Bank | *Caenorhabditis elegans* |
| Cel-CSN6 | gi_392901514 | Gene Bank | *Caenorhabditis elegans* |
| Ath-eIF3f | gi_15225611 | Gene Bank | *Arabidopsis thaliana* |
| Ath-RPN8 | gi_334185250 | Gene Bank | *Arabidopsis thaliana* |
| Ath-CSN6 | gi_18423847 | Gene Bank | *Arabidopsis thaliana* |
| Ani-eIF3f | gi_317030245 | Gene Bank | *Aspergillus niger* |
| Ani-RPN8 | gi_145238878 | Gene Bank | *Aspergillus niger* |
| Ani-CSN6 | gi_317036895 | Gene Bank | *Aspergillus niger* |
| Spo-eIF3f | gi_19113090 | Gene Bank | *Schizosaccharomyces pombe* |
| Spo-RPN8 | gi_19075303 | Gene Bank | *Schizosaccharomyces pombe* |
| Sce-RPN8 | gi_398365985 | Gene Bank | *Saccharomyces cerevisae* |
| Lmj-EIF3F | LmjF.25.1610 | TriTrypDB | *Leishmania major* |
| Lmj-RPN8 | LmjF.32.0390 | TriTrypDB | *Leishmania major* |
| Tbr-EIF3F | Tb927.3.1680 | TriTrypDB | *Trypanosoma brucei* |
| Tbr-RPN8 | Tb927.10.14530 | TriTrypDB | *Trypanosoma brucei* |
| Tva-M67 | TVAG_124160 | TrichDB | *Trichomonas vaginalis* |
| Gdu-RPN7 | GL50803_7896 | GiardiaDB | *Giardia duodenalis* |
| Hsa-eIF3h | gi_4503515 | Gene Bank | *Homo sapiens* |
| Hsa-RPN11 | gi_5031981 | Gene Bank | *Homo sapiens* |
| Hsa-CSN5 | gi_38027923 | Gene Bank | *Homo sapiens* |
| Cel-eIF3h | gi_17505955 | Gene Bank | *Caenorhabditis elegans* |
| Cel-RPN11 | gi_17535703 | Gene Bank | *Caenorhabditis elegans* |
| Cel-CSN5 | gi_17538322 | Gene Bank | *Caenorhabditis elegans* |
| Ath-eIF3h | gi_18391211 | Gene Bank | *Arabidopsis thaliana* |
| Ath-RPN11 | gi_15237785 | Gene Bank | *Arabidopsis thaliana* |
| Ath-CSN5 | gi_42571599 | Gene Bank | *Arabidopsis thaliana* |
| Ani-eIF3h | gi_145239303 | Gene Bank | *Aspergillus niger* |
| Ani-RPN11 | gi_145238428 | Gene Bank | *Aspergillus niger* |
| Ani-CSN5 | gi_317035506 | Gene Bank | *Aspergillus niger* |
| Spo-eIF3h | gi_19114070 | Gene Bank | *Schizosaccharomyces pombe* |
| Spo-RPN11 | gi_19114926 | Gene Bank | *Schizosaccharomyces pombe* |
| Spo-CSN5 | gi_19114043 | Gene Bank | *Schizosaccharomyces pombe* |
| Sce-RPN11 | gi_14318526 | Gene Bank | *Saccharomyces cerevisae* |
| Lmj-EIF3H | LmjF.07.0640 | TriTrypDB | *Leishmania major* |
| Lmj-RPN11 | LmjF.34.0650 | TriTrypDB | *Leishmania major* |
| Tbr-EIF3H | Tb927.8.1170 Tb927.8.1190 | TriTrypDB | *Trypanosoma brucei* |
| Tbr-RPN11 | Tb927.10.2980 | TriTrypDB | *Trypanosoma brucei* |
| Tva-eIF3h | TVAG_105990 | TrichDB | *Trichomonas vaginalis* |
| Tva-M67 | TVAG_159580 | TrichDB | *Trichomonas vaginalis* |
| Gdu-RPN11 | GL50803_16823 | GiardiaDB | *Giardia duodenalis* |

| **eIF3g** | | | |
| --- | --- | --- | --- |
| Hsa-eIF3g | gi_49472822 | Gene Bank | *Homo sapiens* |
| Cel-eIF3g | gi_17533393 | Gene Bank | *Caenorhabditis elegans* |
| Ath-eIF3g | gi_15229743 | Gene Bank | *Arabidopsis thaliana* |
| Ani-eIF3g | gi_145252642 | Gene Bank | *Aspergillus niger* |
| Spo-eIF3g | gi_19112519 | Gene Bank | *Schizosaccharomyces pombe* |
| Sce-eIF3g | gi_6320637 | Gene Bank | *Saccharomyces cerevisae* |
| Lmj-EIF3G | LmjF.34.2700 | TriTrypDB | *Leishmania major* |
| Tbr-EIF3G | Tb927.4.1930 | TriTrypDB | *Trypanosoma brucei* |
| **eIF3i and related proteins** | | | |
| Hsa-eIF3i | gi_4503513 | Gene Bank | *Homo sapiens* |
| Hsa-WDR | gi_5803221 | Gene Bank | *Homo sapiens* |
| Cel-eIF3i | gi_25143635 | Gene Bank | *Caenorhabditis elegans* |
| Cel-WDR5 | gi_17568701 | Gene Bank | *Caenorhabditis elegans* |
| Ath-eIF3i | gi_15225954 | Gene Bank | *Arabidopsis thaliana* |
| Ath-WD40 | gi_15229187 | Gene Bank | *Arabidopsis thaliana* |
| Ani-eIF3i | gi_317027968 | Gene Bank | *Aspergillus niger* |
| Ani-WD | gi_317025385 | Gene Bank | *Aspergillus niger* |
| Spo-eIF3i | gi_19115870 | Gene Bank | *Schizosaccharomyces pombe* |
| Spo-WDPop3 | gi_19112474 | Gene Bank | *Schizosaccharomyces pombe* |
| Sce-eIF3i | gi_6323795 | Gene Bank | *Saccharomyces cerevisae* |
| SceDip2p | gi_6323158 | Gene Bank | *Saccharomyces cerevisae* |
| Lmj-EIF3I | LmjF.36.3880 | TriTrypDB | *Leishmania major* |
| Lmj-WD40 | LmjF.30.0410 | TriTrypDB | *Leishmania major* |
| Tbr-EIF3I | Tb11.01.1370 | TriTrypDB | *Trypanosoma brucei* |
| Tva-eIF3i | TVAG_114460 | TrichDB | *Trichomonas vaginalis* |
| Tva-WD | TVAG_374220 | TrichDB | *Trichomonas vaginalis* |
| Gdu-eIF3i | GL50803_13661 | GiardiaDB | *Giardia duodenalis* |
| Gdu-WD40 | GL50803_33762 | GiardiaDB | *Giardia duodenalis* |
| Mja-eIF3i | gi_10954512 | Gene Bank | *Methanocaldococcus jannaschii* |
| **eIF3j** | | | |
| Hsa-eIF3j | gi_83281438 | Gene Bank | *Homo sapiens* |
| Cel-eIF3j | gi_17509953 | Gene Bank | *Caenorhabditis elegans* |
| Ath-eIF3j | gi_18408426 | Gene Bank | *Arabidopsis thaliana* |
| Ani-eIF3j | gi_145255756 | Gene Bank | *Aspergillus niger* |
| Spo-eIF3j | gi_19114251 | Gene Bank | *Schizosaccharomyces pombe* |
| Sce-eIF3j | gi_6323221 | Gene Bank | *Saccharomyces cerevisae* |
| Lmj-EIF3J | LmjF.25.2120 | TriTrypDB | *Leishmania major* |
| Tbr-EIF3J | Tb927.3.2220 | TriTrypDB | *Trypanosoma brucei* |
| Gdu-eIF3j | GL50803_15546 | GiardiaDB | *Giardia duodenalis* |
| **eIF3k** | | | |
| Hsa-eIF3k | gi_10801345 | Gene Bank | *Homo sapiens* |
| Cel-eIF3k | gi_17564366 | Gene Bank | *Caenorhabditis elegans* |
| Ath-eIF3k | gi_15234123 | Gene Bank | *Arabidopsis thaliana* |
| Ani-eIF3k | gi_145252186 | Gene Bank | *Aspergillus niger* |
| Lmj-EIF3K | LmjF.32.2180 | TriTrypDB | *Leishmania major* |
| Tbr-EIF3K | Tb11.01.7070 | TriTrypDB | *Trypanosoma brucei* |

| **eIF3l** | | | |
| --- | --- | --- | --- |
| Hsa-eIF3l | gi_7705433 | Gene Bank | *Homo sapiens* |
| Cel-eIF3l | gi_17531469 | Gene Bank | *Caenorhabditis elegans* |
| Ath-eIF3l | gi_22327068 | Gene Bank | *Arabidopsis thaliana* |
| Ani-eIF3l | gi_145252512 | Gene Bank | *Aspergillus niger* |
| Lmj-EIF3L | LmjF.36.0250 | TriTrypDB | *Leishmania major* |
| Tbr-EIF3L | Tb927.10.4640 | TriTrypDB | *Trypanosoma brucei* |
